# Noncovalent Interaction Origins of Substrate Discrimination in LinB: A Combined QM/MM and SAPT Analysis

**DOI:** 10.1021/acs.jpcb.6c04481

**Published:** 2026-09-16

**Authors:** Agata Sowińska, Michał Rostkowski, Agnieszka Dybala-Defratyka, Agnieszka Krzemińska

**Affiliations:** † Institute of Applied Radiation Chemistry, Faculty of Chemistry, 49584 Lodz University of Technology, Zeromskiego 114, Lodz 90-543, Poland; ‡ Institute of Physics, Lodz University of Technology, Wolczanska 217/221, Lodz 93-005, Poland

## Abstract

Noncovalent interactions (NCIs) play a fundamental role in ligand organization within enzyme active sites and are increasingly recognized as contributors to selective substrate recognition. The haloalkane dehalogenase LinB provides a suitable system for probing the physicochemical origins of selectivity, as structurally similar substrates exhibit markedly different reactivity. In this work, we investigated LinB-catalyzed dehalogenation pathways for 1,2-dibromopropane (DBP) and the closely related 1,2-dichloropropane (DCP), considering both C1 and C2 nucleophilic attack for each enantiomer. While minimum free-energy pathway calculations reproduced the experimentally observed DBP preference, they did not fully resolve the molecular origin of these selectivity trends within a reaction-coordinate framework. To address this limitation, we applied symmetry-adapted perturbation theory (SAPT) to ensembles of representative structures sampled along the reaction pathways. This interaction-level approach revealed that substrate discrimination arises from a finely balanced interplay of electrostatic attraction, dispersion stabilization, and exchange repulsion, rather than a single dominant factor. Active-site residues preorganize the binding pocket to favor DBP stabilization, whereas DCP experiences weaker stabilizing interactions and increased repulsion, resulting in higher barriers and reduced reactivity. Overall, this study demonstrates the utility of SAPT-based analysis of NCIs for elucidating nonmechanistic contributions to enzyme selectivity beyond conventional pathway approaches.

## Graphical abstract

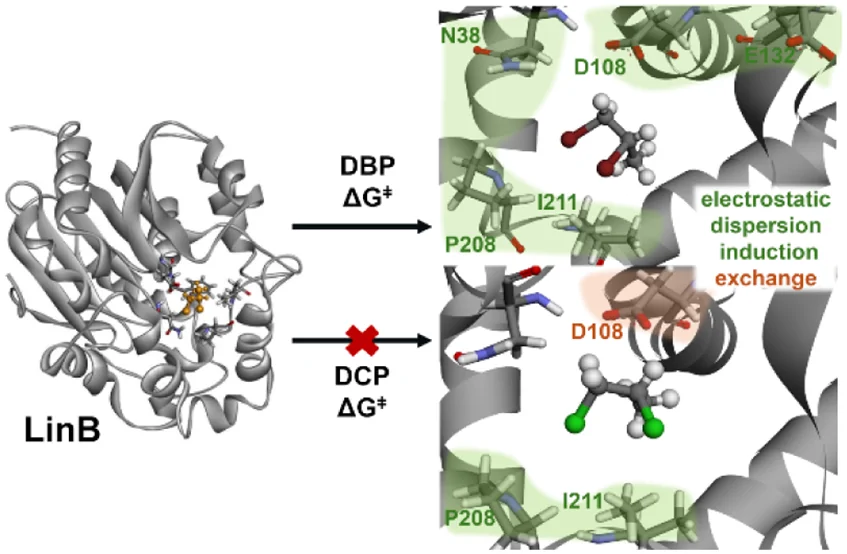

## 1 Introduction

The high specificity and selectivity of enzymatic function arise from the precise spatial organization and properties of active-site residues, which generate a unique network of noncovalent interactions (NCIs) that governs catalytic pathways.^1^ Although individual NCIs are relatively weak, their collective effects are essential for maintaining protein structure, stabilizing nucleic acid assemblies,^2^ and controlling substrate recognition,^3,4^ binding affinity,^5^ and catalytic efficiency.^6,7^ In particular, hydrogen bonding, electrostatic interactions, van der Waals contacts, and hydrophobic effects stabilize enzyme–substrate complexes and transition states, thereby lowering the activation barrier of biochemical reactions.^8,9^ Because these interactions are reversible, enzymes achieve both a high catalytic turnover and substrate specificity.

Accurate characterization of key NCIs within enzyme active sites enables mechanistic elucidation of catalysis,^10^ rationalization of ligand discrimination,^11^ and the accelerated design of protein-targeted drug candidates,^2^ as well as the development of strategies for enzyme engineering.^12^ However, NCIs are orders of magnitude weaker than total system energies, and their accurate description requires careful methodological treatment. While molecular dynamics and QM/MM approaches provide valuable information about enzyme structure, dynamics, and reaction energetics, they do not directly reveal the physicochemical origin of individual enzyme-substrate interactions. Energy decomposition methods partially address this issue by partitioning interaction energies into physically meaningful components.^13–18^ However, many decomposition schemes rely on post hoc partitioning procedures and may be influenced by basis-set effects or error cancellation.^19^

Symmetry-adapted perturbation theory (SAPT) provides an attractive alternative because it directly decomposes interaction energies into electrostatic, exchange, induction, and dispersion contributions while avoiding basis-set superposition error.^20–22^ Such decomposition offers a physically transparent description of ligand-protein interactions and has been increasingly applied to biomolecular systems.^23–25^

In the SAPT framework, the Hamiltonian *Ĥ* of the full system is partitioned into the Hamiltonians of two noncovalently interacting subsystems, $\left({Ĥ}_{A}\right)$ and $\left({Ĥ}_{B}\right)$ , and the intermolecular interaction operator, (V), as shown in eq 1.

1 $$Ĥ={Ĥ}_{A}+{Ĥ}_{B}+V$$

The operator *V* describes all Coulomb interactions between the two subsystems, including electron-electron, nucleus–nucleus, and electron-nucleus contributions. The intermolecular interaction is treated as a perturbation of the isolated monomers. At zeroth order, the SAPT wavefunction (*Ψ* ^(0)^) is represented as the product of the monomer wavefunctions (eq 2):

2 $$\Psi^{\left(0\right)}=\Psi_{A}\Psi_{B}$$

Because this product does not satisfy the required permutation symmetry, an appropriate antisymmetrization procedure is applied. When the intermolecular interaction is treated to second order and Hartree-Fock wavefunctions are used for the isolated monomers, the resulting method is referred to as SAPT0. In this framework, the total interaction energy, *E_int_*, is decomposed into physically meaningful components (eq 3).^21,26^

3 $$E_{int}=E_{elst}+E_{exch}+E_{ind}+E_{exch‐ind}+E_{disp}+E_{exch‐disp}+\delta_{HF}$$

The electrostatic term (*E_elst_*) describes Coulomb interactions between the charge distributions of the monomers. The exchange term (*E_exch_*) originates from wavefunction overlap and the Pauli exclusion principle. Induction contributions (*E*_*ind*_ + *E*_*exch*‑*ind*_) account for polarization and charge-transfer effects induced by one fragment on the other. Dispersion contributions (*E_disp_* + *E_exch_*_‑_ *_disp_*) arise from correlated electron-density fluctuations between the interacting fragments. Finally, the *δ_HF_* correction accounts for higher-order induction effects commonly recovered from the Hartree-Fock interaction energy.

Compared with SAPT0, SAPT2+ introduces correlated corrections that account for intramonomer correlation and extend the perturbation expansion (eq 4), resulting in improved accuracy, particularly for the induction and dispersion components.^27^

4 $$E_{SAPT2+}=E_{SAPT0}+E_{elst}^{\left(12\right)}+E_{exch}^{\left(11\right)}+E_{exch}^{\left(12\right)}+E_{disp}^{\left(21\right)}+E_{disp}^{\left(22\right)}+E_{ind}^{\left(22\right)}+E_{exch‐ind}^{\left(22\right)}$$

Here, $E_{elst}^{\left(12\right)}$ , $E_{exch}^{\left(11\right)}$ , $E_{exch}^{\left(12\right)}$ , $E_{disp}^{\left(21\right)}$ , $E_{disp}^{\left(22\right)}$ , and $E_{ind}^{\left(22\right)}$ represent higher-order corrections to electrostatic, exchange, dispersion, and induction interactions, with the latter accompanied by its corresponding exchange-induction correction, $E_{exch‐ind}^{\left(22\right)}$ . As a result, SAPT2+ yields more reliable absolute interaction energies, especially for systems in which dispersion or polarization plays a substantial role.

The usefulness of SAPT per-residue interaction energy analysis for studying ligand binding has been well established, and its popularity continues to grow due to ongoing methodological advances and increasing computational efficiency.^28–30^ However, despite substantial progress toward extending SAPT applicability to large molecular systems,^22–25,31^ direct SAPT calculations on full protein–ligand complexes remain computationally demanding.

Given these limitations, in this work, we propose a pragmatic, systematic approach to analyzing SAPT interaction energy components. To this end, we conducted extensive population analysis for each investigated reaction pathway to ensure statistically significant sampling. SAPT2+ calculations with the aug-cc-pVDZ basis set were then performed for each obtained representative model to evaluate interactions between the ligand and selected active-site amino acids. The analysis was carried out for both the reactant complexes [RC] and the first transition-state structures [TS1], considering all possible combination of ligand configuration (*R* and *S*) and position of nucleophilic attack (C1 or C2 carbon). In total, 56 complexes were investigated, with 14 ligand–amino acid pairs analyzed for each system. This enabled us to dissect ligand-induced electronic perturbations that modulate catalysis, map active-site polarization patterns, and rationalize LinB’s differential processing of the brominated substrate and its chlorinated analogue. The SAPT energy decomposition affords residue-level quantification of ligand binding contributions, enhancing our mechanistic understanding beyond what is accessible through kinetic or electrostatic analyses alone.

Haloalkane dehalogenases play a key role in the efficient degradation of persistent organic pollutants (POPs) by catalyzing the hydrolytic cleavage of carbon–halogen bonds in halogenated aliphatic compounds, thereby facilitating detoxification and environmental remediation. Among these enzymes, the haloalkane dehalogenase LinB from *Sphingomonas paucimobilis* is distinguished by its broad substrate specificity and enantioselectivity.^32,33^ LinB’s well-established role in the biodegradation of environmentally persistent halogenated pollutants, combined with its well-resolved structural framework and pronounced sensitivity to subtle variations in substrate structure, reflected in the differential stabilization and processing of closely related compounds, makes it a particularly suitable model system for studying the physicochemical determinants of enzyme-substrate recognition and selectivity, including the contribution of noncovalent interactions.^34–37^

LinB exhibits broad substrate specificity, acting on various halogenated compounds, including monochloroalkanes, dichloroalkanes, bromoalkanes, chlorinated aliphatic alcohols, and haloalkanes of varying chain lengths.^38^ Similar to other enzymes in the haloalkane dehalogenase family, LinB catalyses the hydrolytic transformation of haloalkanes into inorganic halide ions and alcohols. The conversion occurs through a two-step mechanism involving the catalytic triad D108, E132, and H272 [see Figure 1]. Although the catalytic mechanism of LinB has been extensively characterized by structural, kinetic, mutagenesis, and computational studies, the rate-determining step has been shown to depend strongly on substrate structure.^39–44^

**Figure 1:**
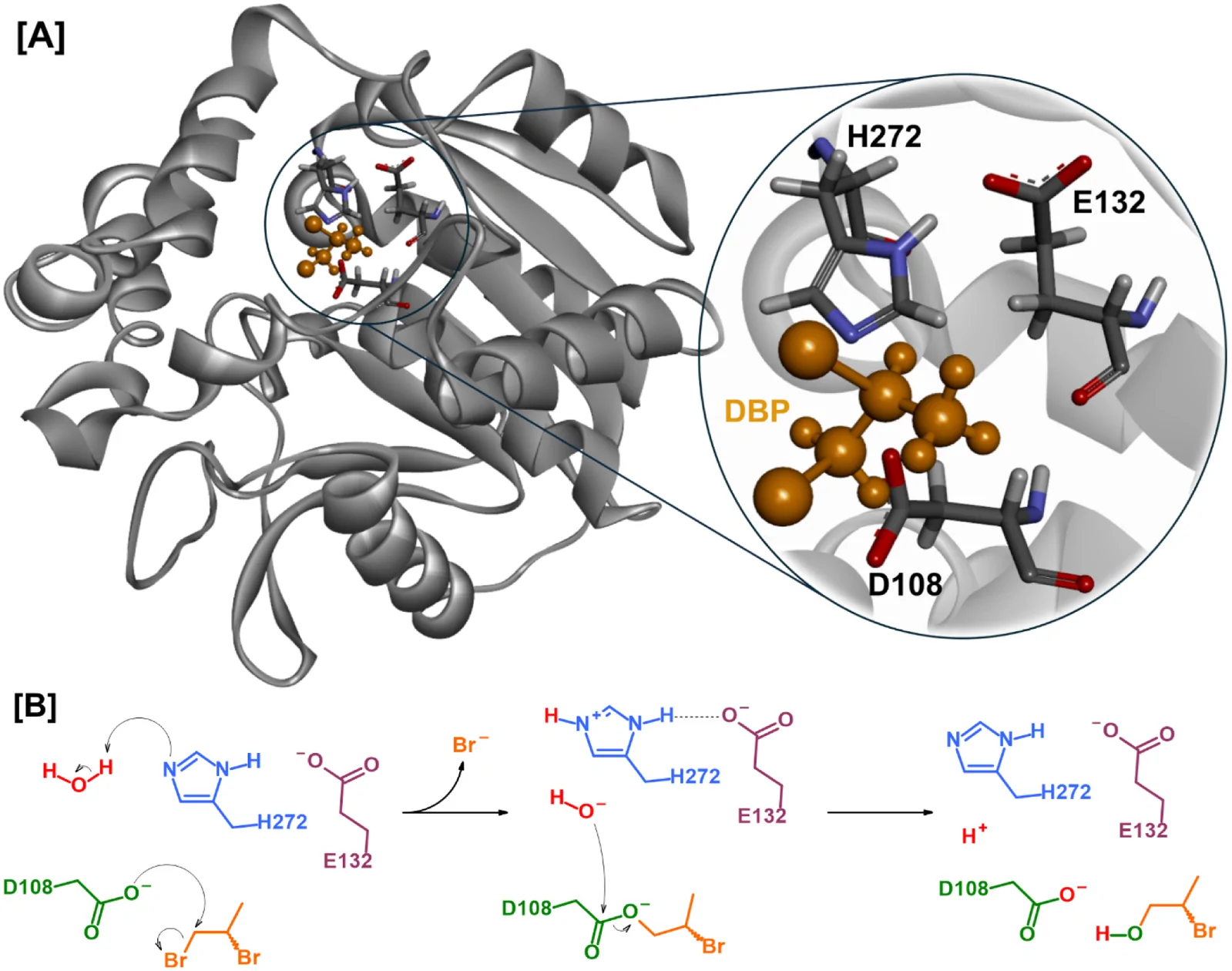
[A] Three-dimensional structure of LinB in complex with (*S*)-DBP with the catalytic triad highlighted. [B] Schematic representation of the LinB-catalyzed dehalogenation mechanism, illustrated by the DBP pathway with nucleophilic attack at the C1 position.

Kmunícek *et al.* investigated the substrate specificity of LinB isolated from strain UT26 by evaluating its steady-state kinetic parameters (K_m_ and *k*_cat_) with halogenated aliphatic and cyclic hydrocarbons.^45^ Their study provided several important insights into the enzyme’s catalytic behavior, including the observation that LinB displays a broad yet nonuniform substrate specificity across structurally diverse compounds. Although numerous chlorinated substrates – such as 1-chlorobutane, 1-chlorohexane, 1,3-dichloropropane, and 1,5-dichloropentane – are efficiently metabolized by LinB with measurable kinetic parameters, the enzyme exhibits distinct metabolic profiles toward closely related dihalopropane derivatives. Despite their close structural similarity, 1,2-dibromopropane (DBP) and 1,2-dichloropropane (DCP) are processed differently. Specifically, steady-state kinetics for LinB with DCP could not be determined due to nonlinear kinetic behavior, whereas DBP exhibited well-defined kinetic parameters indicative of strong substrate binding and efficient catalysis. While it is well established that −R–CH_2_–Cl bonds are intrinsically less susceptible to nucleophilic substitution than −R–CH_2_–Br bonds, even in the absence of an enzyme, owing in part to higher bond dissociation energies (around 80 kcal·mol^–1^ for C–Cl compared with approximately 65 kcal·mol^–1^ for C–Br),^46–49^ this intrinsic reactivity difference alone cannot account for the observed behavior. In particular, the efficient turnover of other chlorinated substrates by LinB indicates that the presence of a chlorine substituent does not, by itself, preclude catalysis. This observation agrees with broader evidence that LinB substrate specificity is governed by a combination of steric, geometric, and energetic factors rather than by halogen identity alone.^45,50,51^ Although stabilization of the leaving halide within the halogen-binding pocket and its subsequent release from the buried active site contribute to catalytic efficiency, activity does not simply follow the leaving-group trend I > Br > Cl. This likely reflects a trade-off between transition-state stabilization and product release, whereby stronger halide stabilization can impede dissociation from the active site.^52^ Consequently, favorable leaving-group properties do not necessarily translate into efficient substrate turnover. Current evidence suggests that LinB primarily selects substrates whose size, shape, and NCIs enable productive accommodation within its U-shaped binding cavity.^33,40,42,51,53^ Therefore, the mechanistic basis for the differential processing of DBP and DCP remains unresolved; however, characterization of the key NCIs governing substrate recognition and binding may provide a molecular-level explanation for the observed selectivity.

To resolve the aforementioned discrepancies and investigate the specificity of LinB-catalyzed reactions, we performed a comprehensive analysis using computational methodologies. Central to our approach was the comparative analysis of reaction pathways for both compounds. While 1,2-DBP has been partially examined in this context in our previous study,^44^ the present work considerably extends this analysis by considering not only substituent effects but also the selectivity of the nucleophilic attack (at the C1 or C2 position) and stereoisomerism of each ligand (*R* and *S* enantiomers). Contrary to the conventional assumption that nucleophilic attack preferentially occurs at the primary C1 position in the LinB-mediated transformation of DBP, experimental evidence indicates that enzymatic conditions favor attack at the secondary C2 position, yielding predominantly 1-bromo-2-propanol as the major product. Although 1,2-propanediol can also be formed, it requires extended reaction times and elevated enzyme concentrations.^33^ This observed regioselectivity prompts further investigation into the molecular determinants governing substrate specificity. In contrast, the regioselectivity of DCP conversion has not been systematically explored. Prior computational studies on the LinB-catalyzed reaction with DCP proposed a reactivity pattern divergent from that of DBP,^54^ however, these studies did not consider the C2 reaction pathway and reported conclusions inconsistent with available experimental data. The alternative sites of nucleophilic attack in DCP thus remain a compelling subject of inquiry, particularly in light of LinB’s demonstrated ability to metabolize its positional isomer, 1,3-dichloropropane.^45^

Taken together, previous experimental and computational investigations have established that LinB processes DBP and DCP differently despite their close structural similarity and have characterized selected reaction pathways and associated free-energy profiles. However, the residue-level interaction patterns underlying substrate discrimination remain unknown, and a systematic comparison of substrate identity, stereochemistry, and reaction position within a unified theoretical framework has not yet been reported. The present work addresses these outstanding questions. To this end, we conducted a comprehensive pathway analysis including all possible combinations: reactions of DBP/DCP with attacks on C1/C2 positions, and considering both *R* and *S* enantiomers for each ligand. We modeled eight Minimum Free Energy Paths (MFEPs) and analyzed them using mean reaction force analysis (MRFA), extending our previous mechanistic analysis of LinB-catalyzed reactions.^44^ This analysis provided a detailed decomposition of the catalytic process and yielded insights consistent with experimentally observed selectivity patterns. However, despite this thorough mechanistic description, the origins of substrate discrimination could not be fully rationalized within a purely reaction-coordinate framework. This limitation highlighted the need to expand the methodological toolbox beyond traditional mechanistic approaches. In this context, the present study demonstrates the utility of SAPT for the analysis of enzyme-ligand interactions, enabling quantitative decomposition of NCIs that govern substrate recognition. By focusing on interaction energy components rather than reaction barriers alone, SAPT provides access to nonmechanistic contributions to selectivity, offering a complementary perspective on enzyme function. Additionally, an important feature of the present approach is that SAPT calculations are not performed on single static structures. Instead, representative conformations are extracted from QM/MM-derived free-energy ensembles and selected through clustering procedure. This strategy enables residue-resolved analysis of noncovalent interactions while accounting for conformational heterogeneity sampled along the reaction pathway. Moreover, comparison of SAPT results between reactant complexes and transition states provides direct insight into how individual interaction components evolve during catalysis.

More broadly, this approach illustrates the potential of SAPT-based NCI analysis as a powerful tool for investigating the physicochemical origins of substrate discrimination in enzymatic systems. Such an interaction-focused framework not only refines the interpretation of enzyme selectivity but also establishes a transferable strategy for studying structure-activity relationships in complex catalytic environments.

## 2 Methods

### 2.1 Model Preparation

Initial coordinates of LinB were taken from Protein Data Bank (PDB code 1MJ5).^42^ Because this structure does not contain a bound substrate, ligands [(*S/R*)-DBP, and (*S/R*)-DCP] positions were determined based on coordinates of the ligands bound within known crystal structures. The DCP model was based on the LinB–1,2-dichloropropane complex (PDB code 1G42),^32^ whereas the DBP model was constructed using structural information from the LinB–1-bromo-2-propanol complex (PDB code 1K6E).^33^ These experimentally determined ligand positions were used as templates for substrate placement within the active site during model preparation. The protonation states of titratable amino acids were determined by the PROPKA 3.1 program^55^ at pH 7. The AM1-BCC charges^56,57^ for the ligands were computed using the Antechamber module of the AMBER package.^58^ ff14SB^59^ and GAFF^60^ force fields were used to describe the enzyme and the ligands, respectively. 12 sodium ions were added to neutralize each system. The enzyme-ligand complexes were solvated with water molecules using the TIP3P water model.^61^ The buffering distance between the edges of the box and the protein was 10 Å. The resulting systems consisted of approximately 35000–40000 atoms. Initial structure energy minimization, heating to 300 K, equilibration, and 100 ns production runs were carried out following the previously described protocol.^44,62^ The simulations were performed using the NPT ensemble with a time step of 1 fs with a Langevin thermostat at 300 K and velocity Verlet integrator.^63,64^ Periodic boundary conditions with the particle mesh Ewald method^65^ and 12 Å cutoff distance for nonbonding interactions were applied throughout the simulations.

### 2.2 Quantum Mechanics/Molecular Mechanics Free Energy Simulations

The reactions of LinB with (*S/R*)-DBP and (*S/R*)-DCP were modeled using a hybrid QM/MM scheme^66^ with the PM3 Hamiltonian to treat the QM part of each model as implemented in the AMBER package as previously described.^44^ The PM3 Hamiltonian was selected based on previous QM/MM investigations of LinB, in which it was shown to accurately reproduce transition-state geometries and kinetic isotope effects, while maintaining computational efficiency required for extensive pathway sampling.^44^ Its limitations in describing absolute reaction energies are mitigated here by focusing on relative trends and complementary interaction energy analysis. The QM region of each complex comprised the ligand molecule, the side chains of D108, H272, E132 residues, and one water molecule (37 atoms in total). The QM region carried a total charge of −2 and was treated as a closed-shell singlet state (multiplicity = 1). To obtain the Minimum Free Energy Paths (MFEPs), the adaptive string method^67^ was used.^44^ The MFEP for the (S)-1,2-DBP-C2 pathway was previously reported,^44^ and is reused here to ensure consistency and direct comparability across all analyzed reaction variants, while all remaining pathways were generated in the present work using the same computational protocol. Replica exchange was applied during both the string optimization and the umbrella sampling stages with exchange attempts performed every 100 fs. A set of four collective variables (CVs) was defined to monitor the dehalogenation and hydrolysis steps of the reaction: (1) the carbon–halogen bond distance at the reactive position (C1 or C2), (2) the distance between the substrate carbon atom (C1 or C2) and the nucleophilic oxygen atom (OD1), (3) the OD1-CG distance within the catalytic nucleophile, and (4) the distance between CG and the oxygen atom of the catalytic water molecule (O_w_). Together, these variables capture the key structural changes associated with both reaction stages. During dehalogenation, CV1 monitors cleavage of the carbon–halogen bond, whereas CV2 describes formation of the covalent bond between the substrate and D108. During hydrolysis, CV3 follows cleavage of the nucleophile-substrate bond associated with transfer of the oxygen atom to the ligand, while CV4 monitors regeneration of the catalytic residue. 50 string nodes were used to follow the reaction progress. The same setup was used consistently for all investigated pathways to ensure methodological uniformity and direct comparability of the resulting free-energy profiles. The string was propagated at the QM/MM level until the root-mean-square deviation (RMSD) of the CVs was approximately 0.1 amu^1/2^ Å for 1.5 to 2 ps. The simulations then enter the umbrella sampling stage along the path CV coordinate to obtain the sampling required to compute the potential of mean force (PMF) profile. Rather than using a predefined simulation length, umbrella sampling was continued until the statistical uncertainty of the dehalogenation free-energy barrier decreased below 1 kcal·mol^–1^. Depending on the reaction pathway, this convergence criterion was typically reached after 25–45 ps of sampling per node. Throughout this work, the reported activation barriers (Δ*G* ^‡^) correspond to free-energy differences between the reactant minimum and the corresponding transition state region identified on the PMF profile.

### 2.3 Mean Reaction Force (MRF) Analysis

MRF methodology introduces the mean reaction force as the negative derivative of the free energy along a predefined reaction path, providing additional insight into the influence of the environment on the reaction mechanism. Moreover, it enables the differentiation between structural and electronic contributions to the free energy barrier in enzymatic reactions.^68,69^ MRF divides the PMF profile along the reaction coordinate into four distinct regions. The initial region, spanning from the reactant state to the minimum of the MRF, primarily reflects structural changes associated with the free energy alteration, denoted as W1 (such as modifications in bond equilibrium length and changes in reactant angles). W2 is more indicative of electronic changes, occurring as bond rupture and formation near the transition state (TS) with minimal structural adjustments. Finally, W3 and W4 delineate the electronic and structural relaxations from the TS to the product states. A comparative analysis of the free energy contributions within each of these regions across different environments offers a comprehensive understanding of the enzymatic influence on the reaction mechanism. The MRF values were calculated by mathematically processing the raw data derived from the computed PMF profiles using OriginPro software.^70^

### 2.4 Selection of the Structures for Analyses of Noncovalent Interactions

Populations of structures representing the designated steps along the reaction pathway were selected using all four CVs defined for the free energy simulations, applying a ±0.05 Å window centered on the values corresponding to each stationary point [see Table S1]. The selections were performed using pytraj,^71^ a Python package binding to CPPTRAJ program^72^ available in AMBER. Each resulting population was subjected to hierarchical agglomerative (bottom-up) clustering, using stopping criteria defined by either a minimum inter-cluster distance threshold, ε, of 1.0 or termination once the number of remaining clusters reached 10. Average inter-cluster distances were calculated based on RMSD value for the heavy atoms of the ligand, the selected first-shell residues previously identified by Kmunícek *et al.* as directly involved in substrate binding and recognition in LinB (N38, D108, W109, E132, I134, F143, F151, F169, V173, W207, P208, I211, L248, H272),^45^ and the catalytic water molecule. For each examined stationary point (RC, TS1), a single representative structure was extracted from every cluster comprising at least 5% of the corresponding population [see Tables S2–S5]. Clustering and calculation of selected parameters and distances for selected structures were performed using the CPPTRAJ module^72^ provided in AMBER. Visualization of analyzed complexes was performed using VMD^73^ and the Discovery Studio BIOVIA Software.^74^ The Cartesian coordinates of the relevant amino acids in complex with the ligand, shown for the representative structures of the largest clusters for each studied reaction pathway, are reported in Tables S6–S9.

Finally, the ligand together with the selected first-shell residues and the catalytic water molecule were extracted from the obtained representative models, and missing connectivities were capped with hydrogen atoms. The resulting structures were used for further studies. In contrast to our previous work, where interaction analyses were performed on structures derived from QM/MM MD simulations,^44^ the present selection is based on PMF-derived ensembles, enabling the investigation of noncovalent interactions along the reaction coordinate, including both reactant and transition-state regions.

### 2.5 Symmetry-Adapted Perturbation Theory Calculations

The high-order SAPT (SAPT2+) with correlation-consistent augmented double Dunning basis (aug-cc-pVDZ)^27^ in the PSI4 software package was used.^75^ When referring to the electrostatic, exchange, induction, and dispersion terms, we group all SAPT2+ contributions belonging to each category, e.g., accounting for dispersion and exchange-dispersion effects, to obtain a single numerical descriptor for each term. The analysis was performed using models of the ligands and the above-mentioned first-shell residues extracted from the structures obtained through clustering. To capture the structural variability within each stationary point, the results were computed as the mean values of the data obtained for the representative structures of all considered clusters. This approach provides a more comprehensive description of the system by accounting for both molecular flexibility and the conformational diversity present across the corresponding structural ensembles. The total interaction energy was then calculated as the sum of the mean contributions from all key interactions within the complex.

### 2.6 Visual Study of Noncovalent Interactions

Visual inspection of NCIs between the ligand, the selected first-shell residues, and the catalytic water molecule extracted from the largest clusters’ representative structures was performed using gradient isosurfaces generated with Multiwfn^76,77^ program employing the independent gradient model based on Hirshfeld partition of molecular density (IGHM).^78–80^ The required wave functions were obtained from single-point calculations at the B3LYP/6-31+G­(d,p) theory level using Gaussian package.^81^ The resulting weak noncovalent interactions surfaces (NCI maps) were visualized in VMD, where the surfaces were color-coded according to sign­(λ_2_)­ρ on an RGB scale to distinguish attractive (blue), repulsive (red), and weak dispersive (green) regions. Visualizations were performed with an isovalue of 0.01 a.u. and a color range of −0.05 a.u. < sign­(λ_2_)­ρ < 0.05 a.u. A schematic representation of the overall workflow adopted in this study is provided in Figure 2.

**Figure 2:**
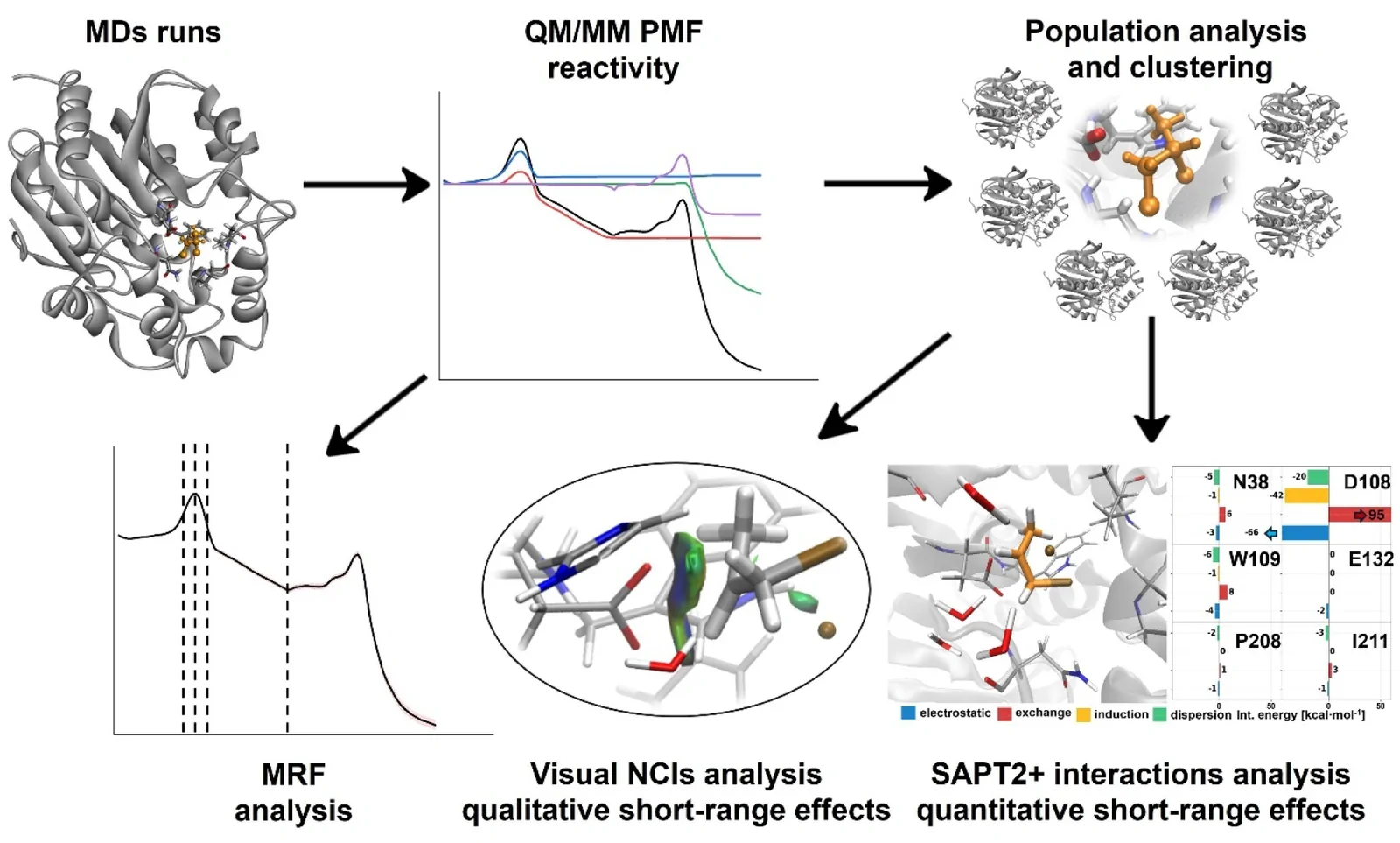
A schematic representation of the overall workflow adopted in the present study.

## 3 Results and Discussion

### 3.1 Free Energy Barriers of LinB-Catalyzed Dehalogenation of DBP and DCP

To investigate all the reactions under consideration, we computed the Minimum Free Energy Pathway for the LinB-catalyzed reaction involving (*S/R*)-DBP and (*S/R*)-DCP. The calculated activation barriers for the dehalogenation step of the reactions under study are presented in Table 1 [for a graphical representation of the complete PMF profiles and evolution of the main CVs along the MFEP see Figures S1–S8]. The (*S*)-1,2-DBP-C2 pathway, reported previously,^44^ is included here as a reference to enable direct comparison across all studied reaction variants.

**Table 1:** The Activation Barriers (Δ*G* ^‡^) Associated with the Dehalogenation Step of the LinB-Catalyzed Reactions with DBP and DCP Depending on the Ligand Configuration and the Position of Nucleophilic Attack [kcal·mol^–1^]

	Ligand			
	DBP		DCP	
Attack position	*S*	*R*	*S*	*R*
C1	15.2	21.4	25.8	32.6
C2	19.5	28.7	35.2	31.5

Based on the obtained data, it is evident that DBP generally has lower energy barriers for the initial reaction step compared to DCP. The lowest energy barrier observed for DCP is comparable to the highest found in DBP. In case of DBP, nucleophilic attack at the C1 atom in the *S* configuration is the most favored. The energy barriers for the first step of *S*-C2 and *R*-C1 reaction variations are higher by approximately 4 and 6 kcal·mol^–1^, respectively. The highest barrier calculated is for the *R*-C2 reaction channel, with the energy of 28.7 kcal·mol^–1^. Similarly, analysis of the first step in the DCP reaction shows that the *S*-C1 pathway is preferred. However, the lowest energy barrier for DCP is approximately 10 kcal·mol^–1^ higher than for its corresponding DBP reaction pathway, with calculated values of 25.8 kcal·mol^–1^ and 15.2 kcal·mol^–1^ for (*S*)-DCP and (*S*)-DBP, respectively. The least favorable nucleophilic attack for DCP occurs at the position C2 of *S* enantiomer with an energy barrier of 35.2 kcal·mol^–1^. These results do not support the previously reported barriers by Tang *et al.*,^54^ likely because – as the authors themselves noted – the reaction barrier is highly sensitive to the specific conformational arrangement of the ligand’s environment. By employing substantially broader conformational sampling, our approach captures these effects more effectively, yielding a more accurate and comprehensive description of the reaction landscape. Collectively, our findings strongly suggest that LinB is unlikely to catalyze the dechlorination of DCP efficiently, regardless of substrate configuration or nucleophilic attack position. The obtained results are in good agreement with previous experimental findings^45^ and clearly indicate that the first step of the enzyme-catalyzed reaction is the key determinant of substrate selectivity in LinB. Nevertheless, these results do not elucidate the molecular basis of the observed differences, as neither the reaction position nor the substrate configuration appear to play a decisive role. Taking into consideration that, as previously mentioned, experimental data reported for 1,3-DCP suggest that the effect cannot be solely attributed to substituent variation, investigating the molecular basis for LinB substrate discrimination was necessary. To gain deeper insight into the observed free energy profiles differences, the structural (W1) and electronic (W2) contributions to the activation barriers associated with the dehalogenation step of the studied reaction pathways were analyzed. The corresponding results are summarized in Table 2.

**Table 2:** The Structural (W1) and Electronic (W2) Contributions to the Activation Barriers Associated with the Dehalogenation Step of the LinB Reactions with DBP and DCP Depending on the Ligand Configuration and the Position of Nucleophilic Attack [kcal·mol^–1^]

	Ligand							
	DBP				DCP			
	*S*		*R*		*S*		*R*	
Attack position	W1	W2	W1	W2	W1	W2	W1	W2
C1	8.9	6.3	14.4	7.0	17.7	8.1	11.0	21.6
C2	11.3	8.2	14.9	13.8	19.6	15.6	22.7	8.8

Decomposition of the activation barriers associated with the dehalogenation step did not reveal a single consistent pattern that could clearly explain the mechanism underlying DCP discrimination by LinB. Although the elevated barriers observed for (*R*)-DBP-C2, (*S*)-DCP-C2, and (*R*)-DCP-C1 are strongly influenced by electronic contributions, substantial structural effects are also apparent in the cases of (*S*)-DCP-C2 and (*R*)-DCP-C2. Notably, the relative contributions of these factors vary significantly across the analyzed pathways, precluding the identification of a unifying descriptor that could rationalize the observed selectivity trends. This variability indicates that a reaction-coordinate-based description, even when refined by mean reaction force analysis, remains insufficient to account for the origins of substrate discrimination. In particular, the interplay between structural and electronic contributions did not yield a consistent relationship between activation barriers and enzyme selectivity, highlighting the limitations of interpreting reactivity solely in terms of energetic profiles. To overcome this limitation, a more detailed examination of the enzyme active site was undertaken, shifting the focus from barrier decomposition to direct analysis of enzyme-ligand interactions.

### 3.2 Noncovalent Interaction Contributions to Substrate Discrimination in LinB

To rationalize the selectivity of LinB toward DBP and DCP observed in the reaction energy barriers, we aimed to uncover the noncovalent interaction-related factors governing its catalytic power. To this end, short-range interaction energies were analyzed within the SAPT framework. It should be noted, however, that the obtained results are to be interpreted comparatively, i.e., by examining relative trends rather than treating the interaction energies as absolute quantities. This limitation arises from the pragmatic choices made in our computational setup. Including the entire LinB protein in the calculations, as implemented in F-SAPT^22^ or EE-SAPT,^25^ would likely provide a more balanced description of short- and long-range interactions and yield substantially lower interaction energies, offering a more complete picture of the noncovalent contributions to substrate binding. However, such calculations remain computationally prohibitive. In our approach, we prioritized generating a large, statistically meaningful dataset using the representative structures selected via cluster analysis. Consequently, the resulting interaction patterns capture the dominant conformational states sampled along the reaction pathway rather than the characteristics of a single selected geometry. SAPT2+ calculations were subsequently performed on the representative structures of the selected stationary points, enabling us to trace residue-specific contributions to their stabilization and to move beyond a purely barrier-based or distance-based description of the reaction mechanism. A particular strength of the adopted approach lies in the direct comparison of SAPT interaction components between the reactant complex and transition state ensembles. Such analysis enables the identification of residues whose energetic contributions change along the reaction coordinate, thereby revealing interaction patterns that govern catalytic stabilization and substrate discrimination. Consequently, SAPT complements conventional free-energy analysis by linking the observed energy barriers to their underlying physicochemical origins.

To elucidate how short-range noncovalent interactions within the active site influence the catalytic activity of LinB, a systematic SAPT analysis was performed to identify key interactions between the ligand and first-shell residues N38, D108, W109, E132, I134, F143, F151, F169, V173, W207, P208, I211, L248, and H272. Based on the total SAPT interaction energies, comprising the sum of electrostatic, exchange, induction, and dispersion components, as well as the individual components themselves, we can ensure an appropriate selection of the quantum region and the subsequent nonpolarizable MM region extending to the full scale of the protein. This selection is guided not merely by spatial proximity (e.g., residues within ∼10 Å), systematic QM region enlargement, or prior experience, but rather by a quantitative measure of each residue’s energetic importance from SAPT. Notably, cases may arise where the total interaction energy between a residue and the substrate is small; however, SAPT decomposition reveals that such weak net interactions result from compensating attractive (electrostatic, induction, and dispersion) and repulsive (exchange) contributions. While our discussion here focuses on the selected key residues: N38, D108, W109, E132, P208, and I211, the complete analysis is provided in the Supporting Information [see Tables S10–S11]. It is worth noting that although H272 forms the LinB catalytic triad together with D108 and E132, its primary role is to stabilize these two residues and to help maintain water accessibility required for catalysis.^39^ Consequently, its contribution to DBP and DCP stabilization is minimal due to its greater distance from the ligands, resulting in lack of H272 influence on ligand discrimination, as supported by SAPT analysis.

The magnitudes of the noncovalent interactions between the selected residues and DBP in both the reactant complexes and the corresponding transition states are shown in Figures 3 *–* [Fig fig4], and structural representations of the active sites are provided in Figures S9–S10. The active-site residues of LinB preferentially stabilize the (*S*)-enantiomer of DBP over the (*R*)-enantiomer. This preference arises mainly from electrostatic and dispersion interactions, with a moderate induction contribution approximately half as strong as the former two. These stabilizing effects originate primarily from residues N38, D108, W109, E132, P208, and I211. The increasingly attractive interactions are counterbalanced by a corresponding rise in the repulsive exchange term. The strongest exchange repulsion, 20–57% stronger than in the other cases and accounting for 38% of the total magnitude of all components of interaction energy between the key residues and the substrate, was observed for the (*R*)-DBP-C2 pathway. This effect arises from its closer interaction with D108 and the absence of compensating dispersion and induction contributions from N38, D108, and P208. For D108 to adopt a favorable position relative to the substrate, its electrostatic stabilization must outweigh the exchange repulsion. This balance is reversed for the (*R*)-DBP-C2 complex, where the exchange term exceeds the electrostatic contribution by more than 10%, leading to an unfavorable energy barrier. Although the overall exchange component is the smallest in the (*R*)-DBP substrate in the C1 pathway compared to the other DBP reactants, it also exhibits the weakest electrostatic contributions, resulting in the lowest overall stabilization among the DBP reactant complexes. For the (*R*)-DBP-C1 complex, the interaction with D108 is 17% and 12% weaker than in the (*S*)-DBP-C1 and (*S*)-DBP-C2 pathways, respectively, where stronger electrostatic and induction contributions from D108 are observed. A further distinction for (*R*)-DBP relative to both (*S*)-DBP pathways is the lack of electrostatic stabilization by E132. An additional disadvantage of the LinB active-site organization for the reaction pathways of the (*R*)-enantiomer is a greater distance between the water molecules and the departing bromide anion, which hinders stabilization of the leaving group.

**Figure 3:**
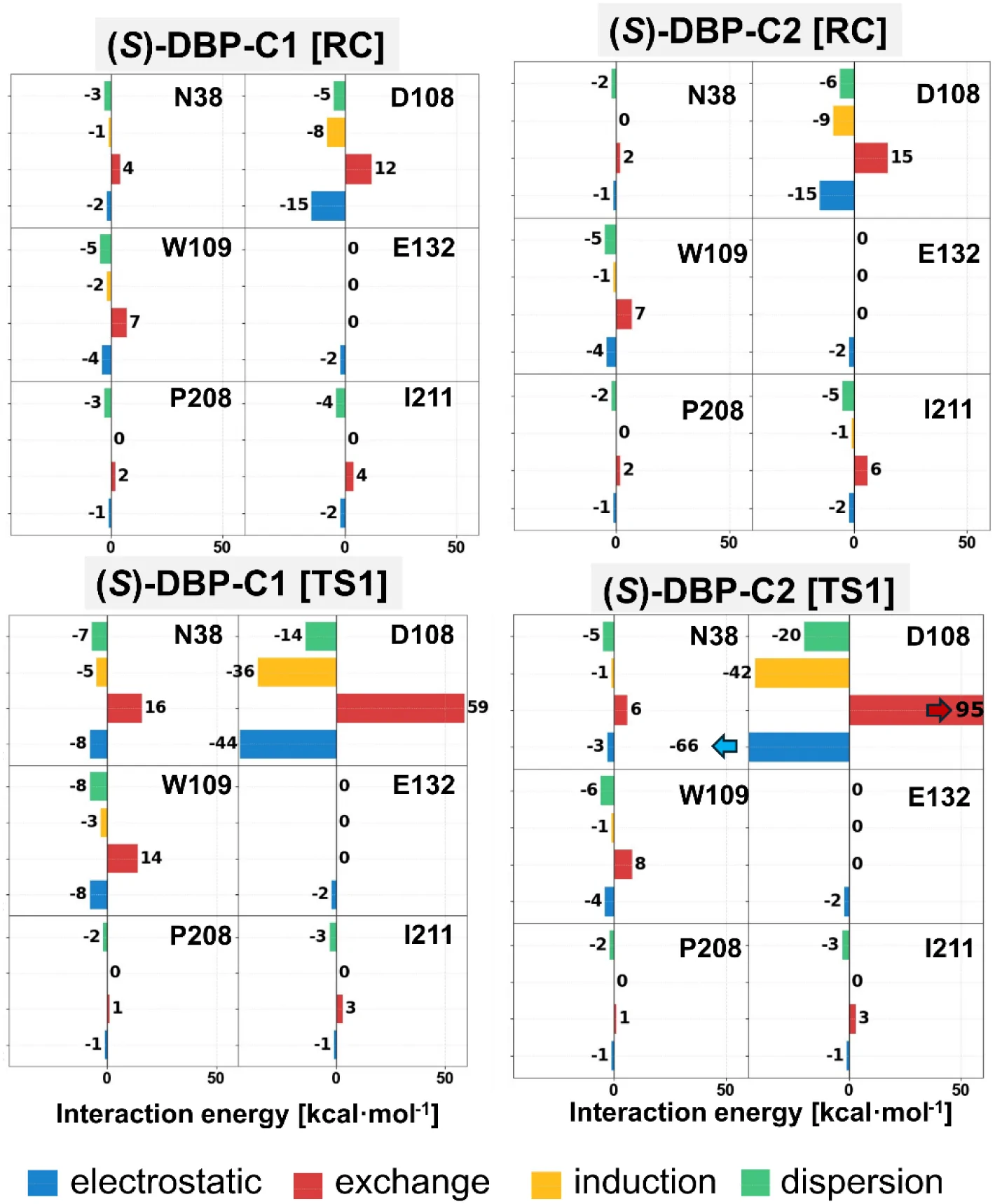
Averaged SAPT noncovalent interactions of DBP in the (S)-C1 and (S)-C2 reactant complexes [RC] and their corresponding transition states [TS1] with key LinB residues. SAPT interaction energies are reported in kcal·mol^–1^.

**Figure 4:**
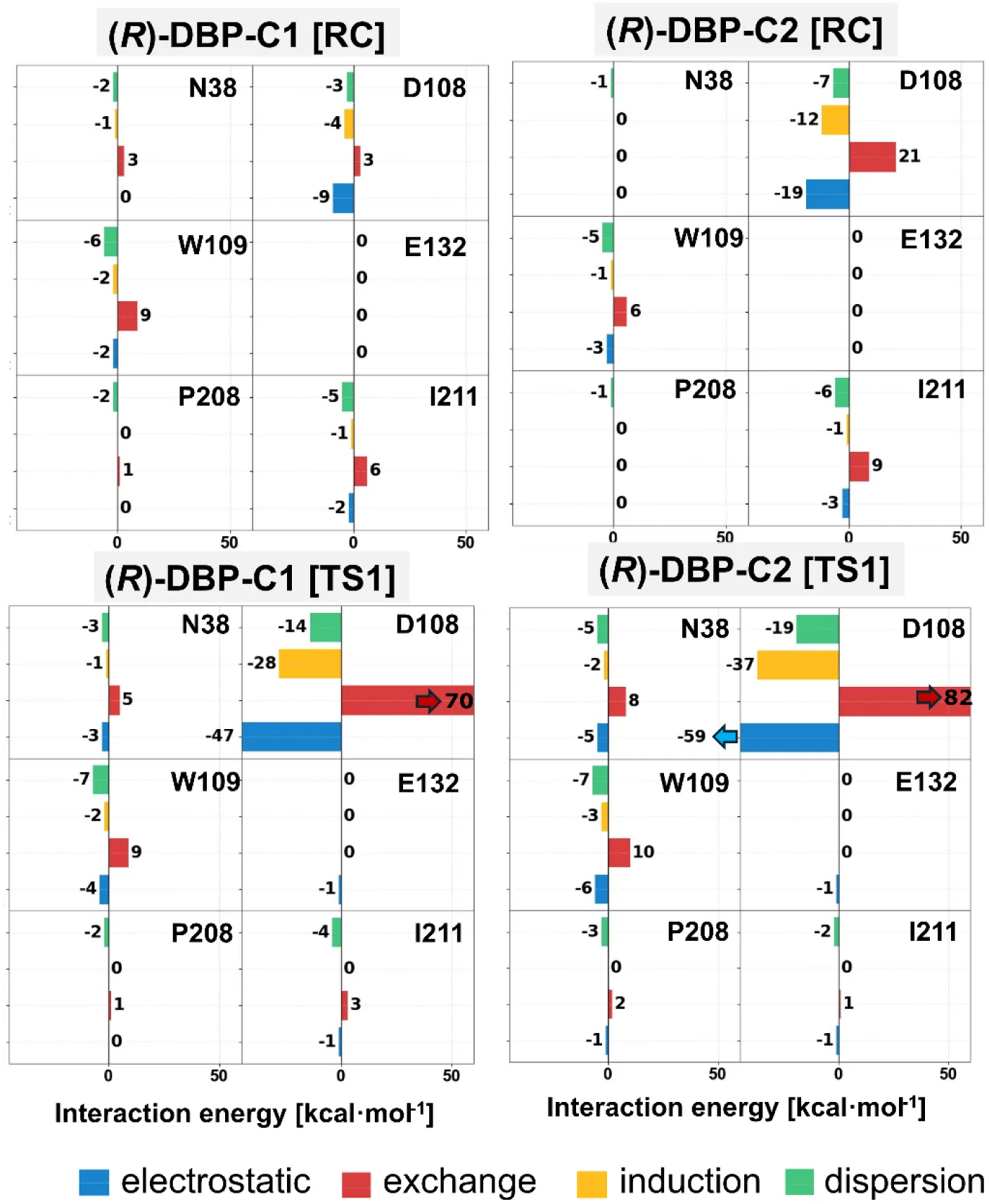
Averaged SAPT noncovalent interactions of DBP in the (R)-C1 and (R)-C2 reactant complexes [RC] and their corresponding transition states [TS1] with key LinB residues. SAPT interaction energies are reported in kcal·mol^–1^.

Proper substrate arrangement within the active site is one of the essential steps for enabling the upcoming reaction, particularly for achieving a favorable distribution of permanent and induced charges, as can be tracked using SAPT electrostatic and induction component analyses.^82^ As residue N38 plays a key role in the transition state, it must also contribute to substrate stabilization, as observed for the (*S*)-DBP substrate of the C1 pathway, where the total attractive interaction with N38 doubles from the reactant to the transition state. Following this line of reasoning, residue P208 should also contribute to the stabilization of the reactants, since its involvement in the transition state is necessary to stabilize the departing bromide anion. This can be observed for (*S*)-DBP-C1. In this case, the total interaction with P208 accounts for approximately 8% of the reactant complex stabilization and 4% of the transition state stabilization. For the (*S*)-DBP substrate of the C2 pathway, both N38 and P208 contribute to stabilization, although with slightly lower magnitudes due to less favorable relative orientations [see Table S12]. On the other hand, in the (*S*)-DBP-C2 transition state, a much stronger (36%) attraction of D108 is observed due to the greater geometric accessibility of the C2 carbon relative to C1. This interaction increases the polarization of the active site (reflected in the combined electrostatic and induction SAPT components), thereby lowering the energetic cost of the subsequent reaction step. Although this substantial rise in electrostatics is naturally accompanied by a proportional increase in exchange, the resulting enhancement of active-site polarization can stabilize the ionic leaving group and strengthen electrostatic preorganization, one of the primary catalytic strategies employed by enzymes.^83^

The magnitudes of the noncovalent interactions between the selected residues and DCP in reactant complexes, as well as their corresponding transition states, are shown in Figures 5–6, and structural representations of the active sites are provided in Figures S11–S12. The positioning of the DCP molecule relative to D108 is generally unfavorable at the reactant stage, as indicated by SAPT exchange terms exceeding the corresponding electrostatic contributions by 9–20%. This imbalance becomes even more pronounced in the transition states (20–36%). Such unfavorable interactions contribute to the high energy barriers observed for DCP. The only case in which the exchange term does not dominate over the electrostatic contribution is the (*S*)-DCP-C1 reactant complex, where the electrostatic component is also the weakest. In this variant, the substrate is positioned slightly farther from the LinB active-site residues, a geometry imposed by directing the nucleophilic attack of D108 toward C1 and the associated rotation of the ligand [see Table S12]. The resulting reduction in close-contact exchange repulsion aligns with (*S*)-DCP-C1 being the least energetically unfavorable among all examined DCP pathways.

**Figure 5:**
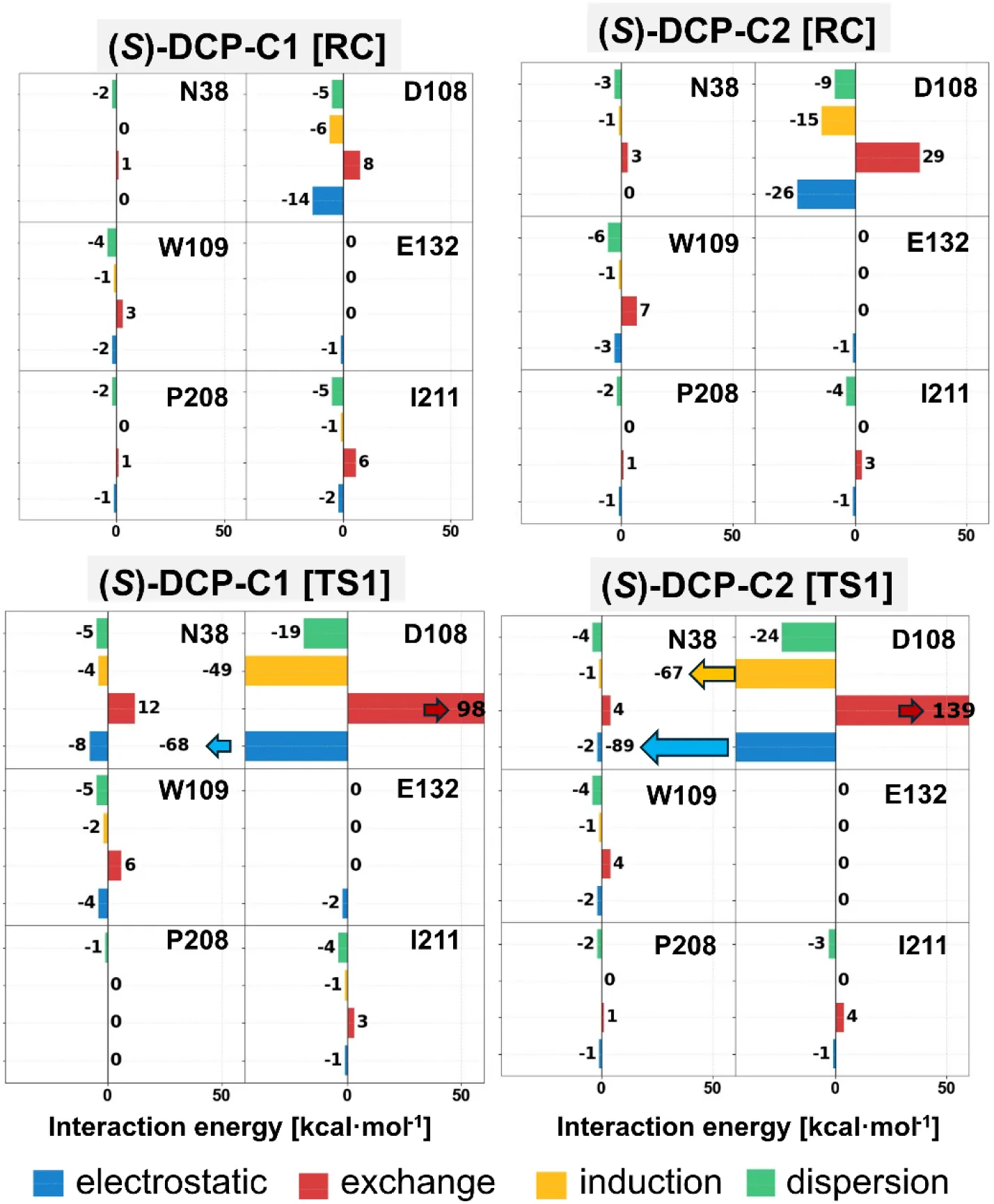
Averaged SAPT noncovalent interactions of DCP in the (S)-C1 and (S)-C2 reactant complexes [RC] and their corresponding transition states [TS1] with key LinB residues. SAPT interaction energies are reported in kcal·mol^–1^.

**Figure 6:**
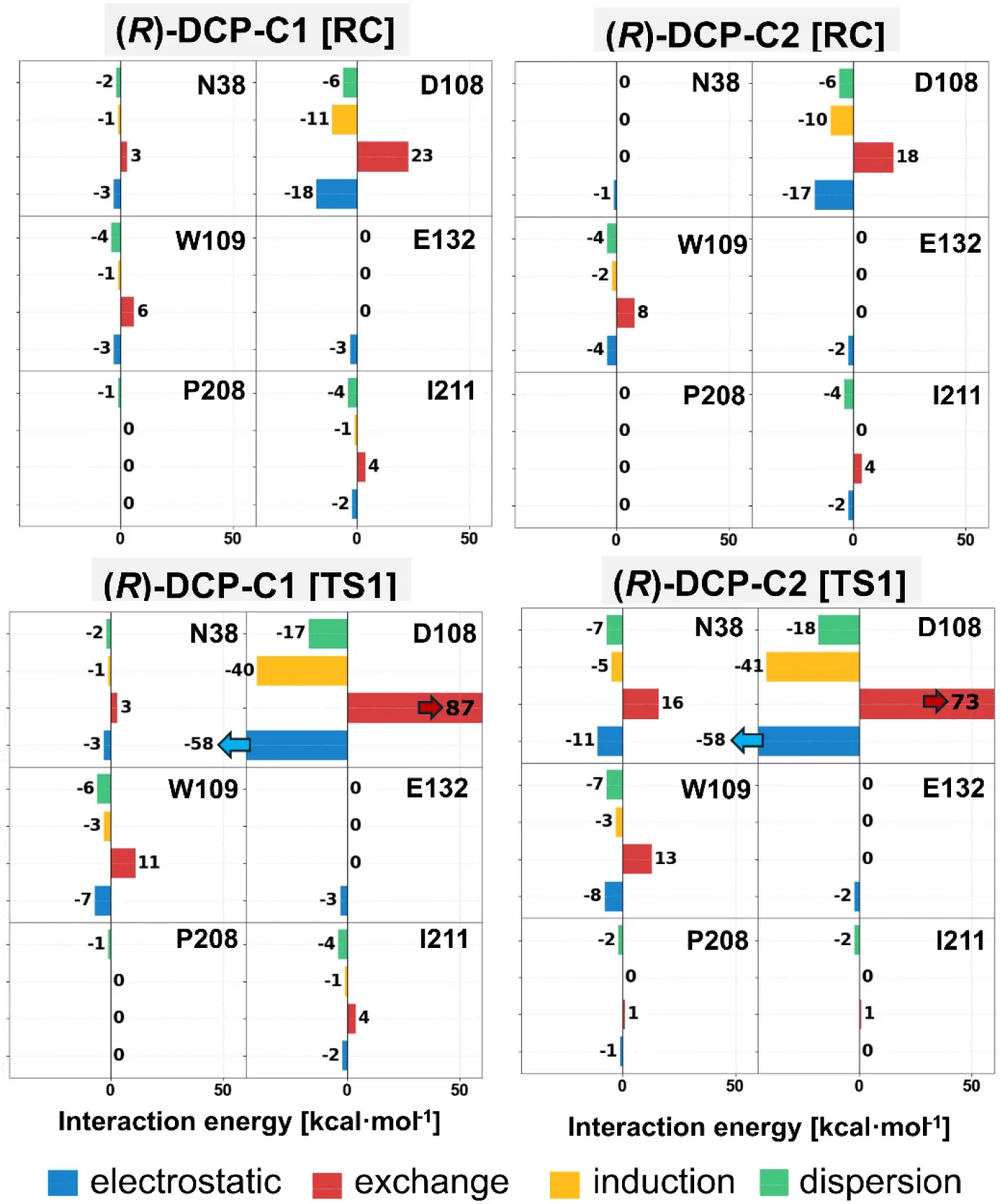
Averaged SAPT noncovalent interactions of DCP in the (R)-C1 and (R)-C2 reactant complexes [RC] and their corresponding transition states [TS1] with key LinB residues. SAPT interaction energies are reported in kcal·mol^–1^.

As in the case of the bromo derivative in the *R* configuration, the absence of N38 attraction toward the DCP substrates leads to an increased energy barriers. This observation confirms the crucial role of N38 in the first step of the LinB-catalyzed dehalogenation, consistent with previous reports.^62,84^ The residue contributes to transition-state stabilization by interacting with the leaving group and must therefore be properly positioned in the substrate prior to catalysis. Although P208 is generally slightly better positioned relative to the DCP substrates than to their brominated counterparts, this is not reflected in its interaction energies, and the residue exerts only a negligible effect on stabilizing the departing chloride anion.

Residues W109, E132, and I211 stabilize the DCP substrates and transition states with interaction energy values similar to those obtained for DBP, rendering the two ligands indistinguishable in this respect.

The final important conclusion for DCP arises from a careful examination of the pronounced exchange contribution in the transition states. The substantial increase in exchange observed in those complexes is likely overestimated, since the monomer partition can cut through regions of partial covalent character, unfavorable for SAPT, such as the halogen bond present here. In such cases, the density overlap is interpreted as “forbidden” rather than “shared”, leading to an artificial enhancement of exchange repulsion.^85^ However, the same effect is also present for DBP, indicating that the absolute values are less meaningful than their relative trends. Nonetheless, in DCP, the smaller substrate can penetrate more deeply into the active site and approach residues such as D108 more closely, increasing frontier occupied–occupied orbital overlap and thereby amplifying the exchange repulsion.

The environment of the leaving group in the LinB active site, comprising residues N38, P208, and I211, along with nearby water molecules, strongly influences the reaction mechanism. The bromide anion, being a weaker base than chloride, serves as a better leaving group in a protic environment. Although the larger atomic radius of bromine makes it less susceptible to S_N_2 substitution, it allows the DBP substrate to adopt a more favorable orientation within the active site. The substrate is positioned far enough from D108 to prevent electron-density overlap, unlike in the DCP–D108 complex, while the greater size of bromine also enables interaction with N38, effectively stabilizing the leaving group. The presence and accessibility of water molecules near the leaving-group departure site are also crucial, and the more central positioning of DBP within the active site facilitates such stabilization.

Alongside the SAPT quantitative analysis, a qualitative weak intermolecular interaction analysis was performed to provide an additional perspective on noncovalent interactions and electronic evolution during the LinB-catalyzed reaction. The obtained NCI maps for selected stationary structures are presented in Figures 7–8, using an isovalue of 0.01 a.u. and a color range of −0.05 a.u. < sign­(λ_2_)­ρ < 0.05 a.u. to distinguish attractive (blue), repulsive (red), and weak dispersive (green) regions. The structures shown in the figures correspond to the reaction pathways exhibiting the lowest and highest energy barriers for each ligand [see Table 1], while the respective visualizations for the remaining stationary points are provided in Figure S13.

**Figure 7:**
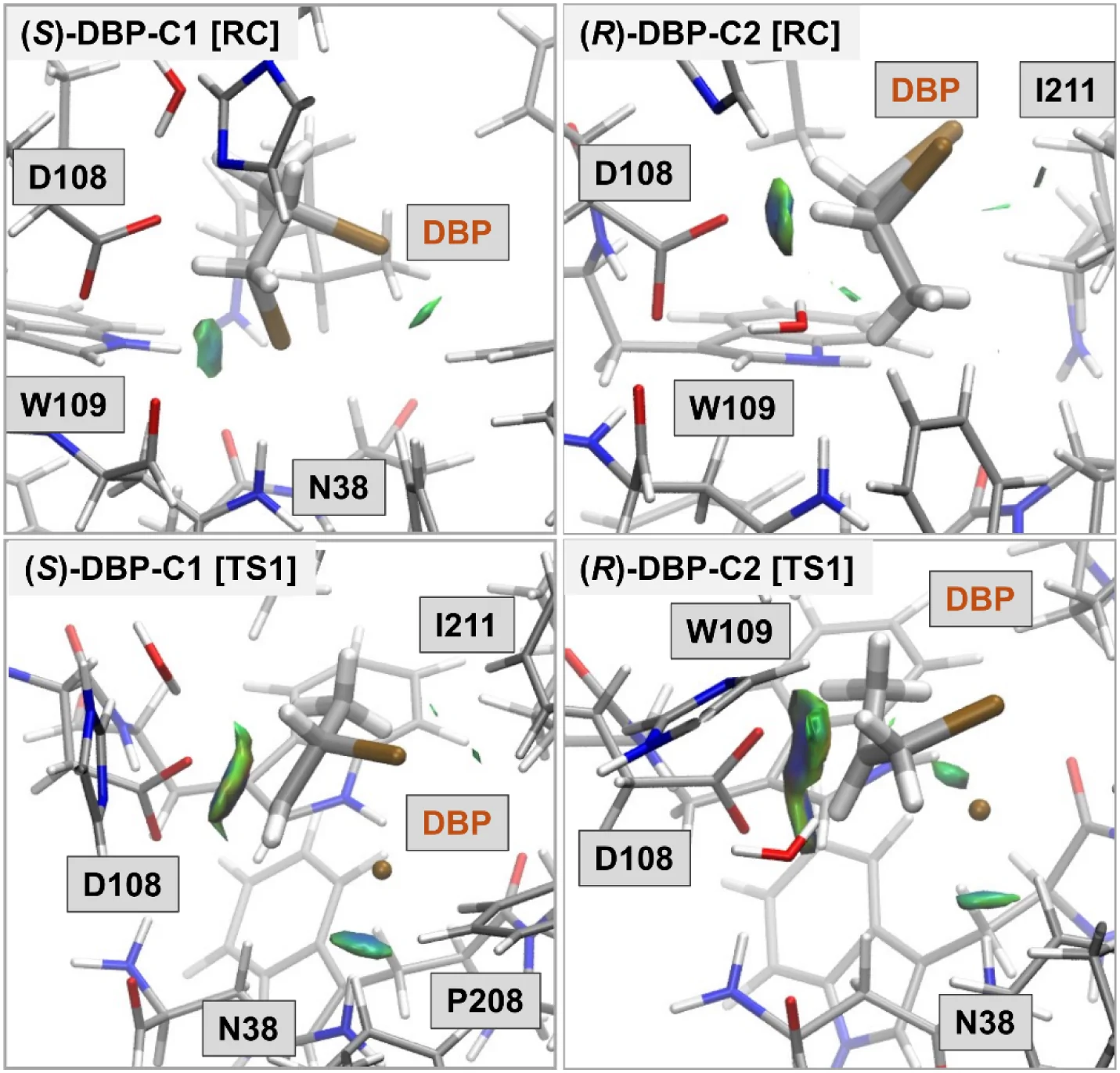
Weak intermolecular interaction maps for selected stationary structures of DBP in the LinB active site. The surfaces are color-coded according to sign­(λ_2_)­ρ on an RGB scale to distinguish attractive (blue), repulsive (red), and weak dispersive (green) regions. Visualization was performed with an isovalue of 0.01 a.u. and a color range of −0.05 a.u. < sign­(λ_2_)­ρ < 0.05 a.u.

**Figure 8:**
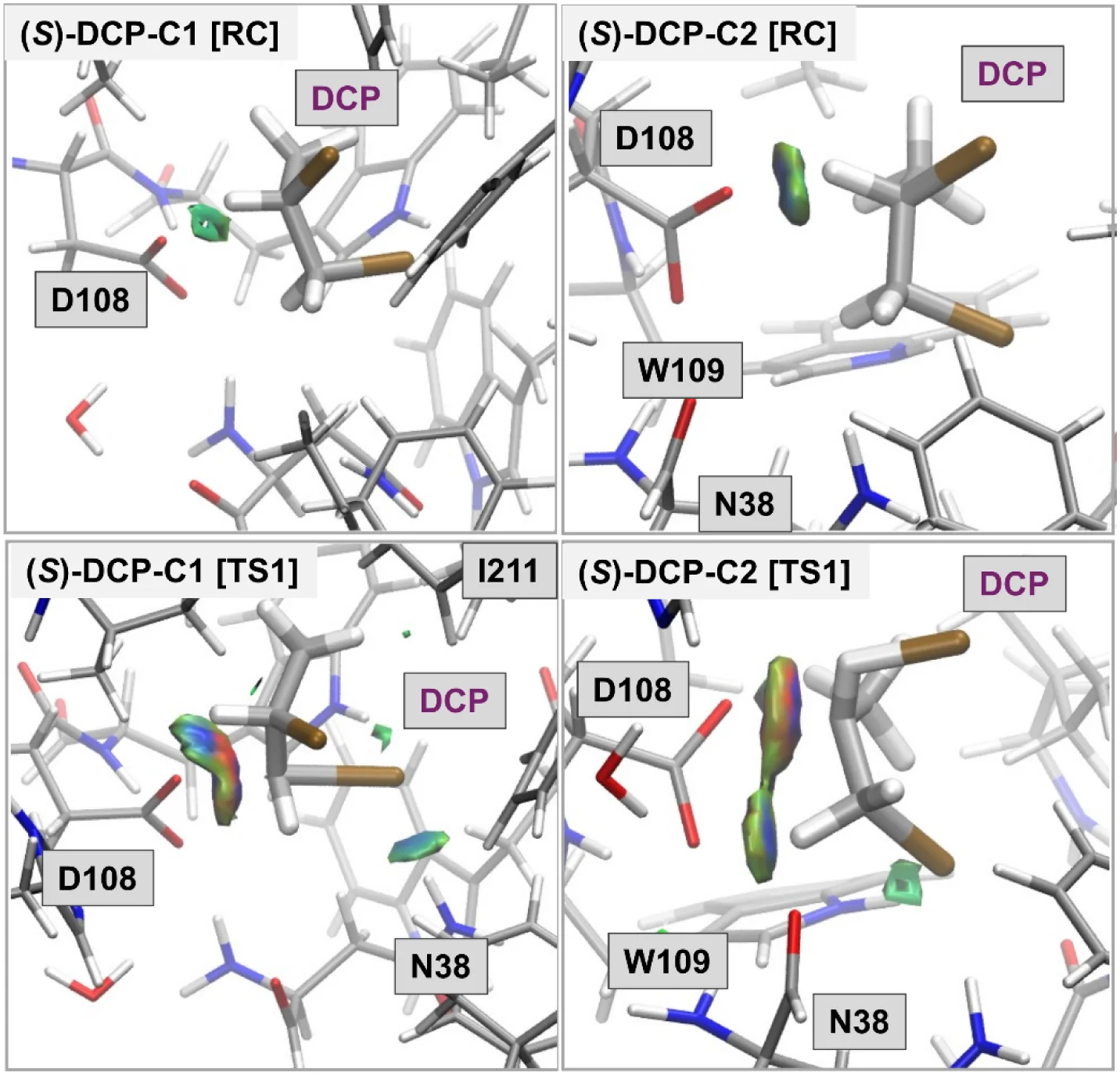
Weak intermolecular interaction maps for selected stationary structures of DCP in the LinB active site. The surfaces are color-coded according to sign­(λ_2_)­ρ on an RGB scale to distinguish attractive (blue), repulsive (red), and weak dispersive (green) regions. Visualization was performed with an isovalue of 0.01 a.u. and a color range of −0.05 a.u. < sign­(λ_2_)­ρ < 0.05 a.u.

For DBP, the conclusions drawn from the noncovalent interaction maps are consistent with the SAPT results, confirming a lower repulsive exchange contribution in the transition states, particularly for the (*S*)-DBP substrate, and stabilization arising from polarization (electrostatic and induction) interactions between (*S*)-DBP and D108. Visual analysis also indicates stabilization of the leaving group already at the reactant stage, which persists in the transition state.

For the (*S*)-DCP reactant complexes, the interaction maps reveal an attractive interaction with D108. However, there is a clear absence of other noncovalent interactions – most notably, no preorganized interactions supporting the stabilization of the leaving group are observed. This finding is further supported by the SAPT analysis, which also indicates a lack of, or only very weak, interactions between N38, P208 and the DCP substrate. Moreover, the SAPT results for the DCP transition states reveal a pronounced increase in the exchange component. This effect is also evident in the NCI map, where the sign­(λ_2_)­ρ values between D108 and DCP display red and yellow regions corresponding to enhanced repulsive interactions. In line with the SAPT results, the NCI maps for the DCP transition states reveal the emergence of an interaction with N38, supporting the leaving group and appearing only in the transition states but absent in the reactant complexes.

Overall, NCI results support the SAPT findings, showing that substrate-dependent noncovalent interactions control activation and leaving-group stabilization in the LinB-catalyzed reaction.

### 3.3 Regioselectivity of LinB–DBP Reaction

Based on the computed free energy barriers, the first step of the LinB–DBP reaction appears energetically accessible for three of the examined pathways (*S*–C1, *S*–C2, and *R*–C1), with the lowest activation barrier predicted for the (S)-DBP-C1 pathway. At first glance, this result seems inconsistent with the experimentally observed product distribution, in which 1-bromo-2-propanol, formed via nucleophilic attack at the C2 position, is the predominant product. This apparent discrepancy suggests that regioselectivity cannot be inferred solely from the energetics of the dehalogenation step and instead requires consideration of the complete catalytic sequence, including the hydrolysis of the alkyl–enzyme intermediate. The calculated activation barriers (*E*_a_) associated with the hydrolysis step of the reaction pathways in question and their structural (W1) and electronic (W2) contributions are presented in Table 3. The (*S*)-1,2-DBP-C2 pathway, described above and reported previously,^44^ is included here as a reference to enable direct comparison across all studied reaction variants.

**Table 3:** The Activation Barriers (Δ*G* ^‡^) and Their Structural (W1) and Electronic (W2) Contributions Associated with the Hydrolysis Step of the LinB Reaction with DBP Depending on the Ligand Configuration and the Position of Nucleophilic Attack [kcal·mol^–1^]

	Attack position		
Ligand DBP	ΔG^⧧^	W1	W2
	C1		
*S*	26.0	16.9	9.1
*R*	29.2	26.2	3.0
	C2		
*S*	15.8	12.8	3.0

It is important to note that, unlike the dehalogenation step, which can be effectively analyzed in terms of pairwise interactions between the substrate and key active-site residues, the hydrolysis step involves a more complex, concerted mechanism, including nucleophilic attack by a water molecule and reorganization of the catalytic triad. As a result, this stage of the reaction is governed by inherently many-body effects arising from the cooperative interaction of multiple fragments within the active site. In this context, the applicability of SAPT is limited, as the method is fundamentally formulated for two-body interaction problems and its straightforward extension to multi-component systems remains nontrivial and computationally demanding.^21,26,27^ Consequently, while SAPT provides a powerful framework for analyzing noncovalent interactions in the dehalogenation step, its use in describing the hydrolysis step is less direct, and the interpretation of this stage relies on analysis of free energy profiles and their structural and electronic decomposition

Extending the investigation to the second step of the enzymatic reaction revealed that, although nucleophilic attack on DBP most likely occurs at the C1 position of *S* enantiomer, the second reaction step is energetically unfavorable for that position (regardless the ligand configuration). The MRF analysis of the potential LinB–DBP reaction pathways revealed pronounced differences in the relative contributions of structural (W1) and electronic (W2) factors to the overall activation barrier. While the W2 results correlate with the previous SAPT observations associating a high electrostatic component in the transition state with a favorable energetic cost of the subsequent reaction step, the hydrolysis step is governed primarily by structural effects. The elevated W1 values observed for this step largely account for the higher energy barriers associated with the *S*–C1 and *R*–C1 pathways, rendering *S*–C2 the only energetically feasible one. Further analysis of the PMF profiles derived from the string method enabled evaluation of the individual contributions of each collective coordinate (CV) to the total activation barrier [see Table 4]. The corresponding decomposed profiles are presented in Figure S14. Minor discrepancies (±0.1) between the total values in Table 3 and the sums of the individual contributions in Table 4 arise from rounding of the individual components.

**Table 4:** The Individual Contributions of Each Collective Coordinate to the Total Activation Barrier Associated with the Hydrolysis Step of the LinB Reaction with DBP Depending on the Ligand Configuration and the Position of Nucleophilic Attack [kcal·mol^–1^]

	Attack position			
	C1			
Ligand DBP	C1-Br1	C1-OD1	OD1-CG	CG-O_w_
*S*	–0.3	0.0	5.1	21.3
*R*	0.1	0.0	2.8	26.2

	C2			
	C2-Br2	C2-OD1	OD1-CG	CG-O_w_
*S*	–0.1	0.0	0.5	15.3

Analysis of the free energy profiles for individual CVs revealed that the energy change associated with the CG-O_w_ distance is the key contributor to the activation barrier in the hydrolysis step of the LinB reaction with DBP. The substantial increase in the energy barrier related to hydroxide attack, together with the lack of stabilization of the forming CG-O_w_ bond, result in unfavorable energetic profile for the C1 pathways. This observation aligns with previously discussed results emphasizing the dominant role of structural rearrangements during this stage of the enzymatic reaction as the CG-O_w_ coordinate corresponds to the regeneration of D108 – a process involving extensive structural reorganization within the active site, including repositioning of the water molecule, adjustment of the side chains of participating and neighboring residues, and displacement of the departing alcohol. The significance of the structural contributions also explains why this step ultimately dictates the regioselectivity of LinB-catalyzed DBP transformation. Accordingly, the hydrolysis barrier analysis indicates that the reaction can proceed efficiently only via the *S*-C2 pathway, in agreement with experimental observation identifying 1-bromo-2-propanol as the major product in the reaction.^45^ These findings demonstrate that, despite the kinetic preference for nucleophilic attack at C1 during the dehalogenation step, the hydrolysis step emerges as an important contributor to the experimentally observed predominance of the C2-derived product, highlighting that the overall selectivity of the LinB-catalyzed DBP transformation cannot be inferred from the dehalogenation step alone.

## 4 Conclusions

LinB exhibits broad substrate specificity yet processes 1,2-dibromopropane (DBP) and 1,2-dichloropropane (DCP) differently, despite their close structural similarity. Previous experimental studies established the existence of substrate discrimination and regioselectivity in LinB, while earlier QM/MM investigations characterized selected reaction pathways and their free-energy profiles. The present work extends these findings by providing a unified analysis of all combinations of substrate identity (DBP/DCP), stereochemistry (R/S), and nucleophilic attack position (C1/C2), and by linking the resulting reactivity patterns to specific residue-level interaction motifs within the active site.

To identify the factors governing preferential processing of DBP over DCP, we adopted a computational workflow combining the Minimum Free Energy Pathway analysis of the LinB-catalyzed dehalogenation of (*S/R*)-DBP and (*S/R*)-DCP with a detailed characterization of noncovalent interactions in the enzyme active site using SAPT. The SAPT analysis was employed to identify relative differences in residue-substrate interactions and active-site stabilization patterns, providing a detailed interaction-level perspective on the origin of substrate selectivity. In addition to enantioselectivity, regioselectivity was considered by analyzing two possible reaction pathways for each ligand – one involving nucleophilic attack at C1 and the other at C2 position.

The computed free-energy profiles clearly captured the experimentally observed discrimination against DCP and highlighted the important role of structural contributions to the hydrolysis step, which render the C1-derived pathways less favorable than the investigated C2 pathway and thereby support the experimentally observed predominance of the C2-derived product in the LinB-catalyzed DBP transformation. However, decomposition of the activation barriers did not reveal a single dominant factor governing selectivity, but instead pointed to a nonuniform interplay of structural and electronic effects across the analyzed pathways. This lack of a clear, consistent pattern from barrier decomposition motivated a more detailed analysis of enzyme-ligand interactions at the molecular level. In this context, interaction-level analysis provided additional insight into the origin of LinB’s substrate selectivity, indicating that its catalytic efficiency arises from a delicate balance between competing attractive and repulsive interaction components. Importantly, although previous QM/MM studies successfully reproduced selected aspects of LinB reactivity, the present expanded reaction-space analysis demonstrates that neither substrate identity, stereochemistry, nor reaction position can be understood in isolation, emphasizing the importance of considering the complete set of accessible pathways.

The pre-organization of key residues N38, D108, W109, E132, P208, and I211 in the active site, which facilitates favorable substrate orientation and transition-state stabilization, explains LinB’s preference for (*S*)-DBP. In contrast, DCP experiences weaker stabilization (due to the absence of N38 and E132 support) and stronger electronic repulsion (particularly from D108). Moreover, the smaller size of DCP causes it to shift toward D108, increasing overlap and exchange repulsion. Collectively, these effects account for the higher reaction barriers and reduced catalytic efficiency of LinB toward DCP relative to DBP.

Furthermore, the analysis highlights that the local environment of the leaving group, formed by residues N38, P208, and I211, together with nearby water molecules, plays an important role in governing the catalytic efficiency of LinB. The weaker basicity and larger size of bromide, compared with chloride, make bromine a superior leaving group in the protic environment of the active site. The DBP substrate benefits from this effect, adopting a more favorable orientation that minimizes repulsion with D108 and enables stabilizing interactions with N38 and water molecules. In contrast, the shorter C–Cl bond in DCP and its less favorable spatial arrangement result in weaker stabilization of the leaving group and contribute to the higher reaction barriers observed for this substrate.

Finally, the visual NCI analysis qualitatively corroborates the SAPT findings, showing that substrate-dependent noncovalent interactions control activation and leaving-group stabilization in the LinB-catalyzed reaction. For DCP, only D108 interacts attractively in the reactant complex, with leaving-group stabilization appearing only at the transition state. In contrast, DBP shows earlier and stronger stabilizing interactions, consistent with lower repulsion and enhanced electrostatic and inductive effects.

Beyond the specific conclusions regarding DBP and DCP reactivity, this study demonstrates the utility of combining QM/MM free-energy simulations, clustering analysis, and SAPT calculations for investigating enzyme-substrate interactions. The use of PMF-derived conformational ensembles and residue-resolved RC-to-TS interaction analysis provides a practical framework for linking catalytic free-energy landscapes with physically meaningful descriptions of noncovalent interactions in enzymatic systems. An additional contribution of this study is the integration of multiple selectivity determinants, namely substrate identity, stereochemistry, and reaction position, within a single computational framework. This comprehensive perspective was not available from previous experimental or theoretical investigations, which focused on selected substrates or individual reaction pathways.

Overall, this study demonstrates that substrate discrimination by LinB cannot be attributed to a single mechanistic factor but arises from a complex interplay of noncovalent interactions within the enzyme active site. In this context, SAPT provides a rigorous and physically transparent framework for decomposing interaction energies into fundamental components, thereby enabling a detailed characterization of the balance between stabilizing and destabilizing contributions that govern enzymatic selectivity. By complementing free-energy analysis with interaction-level resolution, this approach allows access to determinants of reactivity that are not readily apparent from energy profiles alone. Consequently, the present work provides a more comprehensive mechanistic picture of both regio- and enantioselectivity in LinB-catalyzed reactions and highlights the potential of SAPT as a complementary tool for analyzing noncovalent interaction patterns in enzymatic reaction mechanisms.

## Supplementary Material



## Data Availability

The research data supporting this study have been deposited in the Zenodo repository and are available at 10.5281/zenodo.21904736.^86^

## Notes

Cluster analysis parameters and results; Cartesian coordinates of the LinB first-shell amino acids in complex with the ligands for the representative structures; free energy profiles for all the studied reaction pathways – overall and variable-decomposed; complete results of SAPT2+ calculations; visualization of the active sites of the representative structures; selected geometrical parameters in the active site; intermolecular interaction maps for the remaining stationary structures within the LinB active site (PDF)
