# Supplementary material for: Noncovalent Interaction Origins of Substrate Discrimination in LinB: A Combined QM/MM and SAPT Analysis
Source: J Phys Chem B. 2026 Sep 16;130(39):10175–91. doi: 10.1021/acs.jpcb.6c04481 (PMC13637021; doi:10.1021/acs.jpcb.6c04481)
Supplement: Supplementary file 1 [file jp6c04481_si_001.pdf]

Supporting Information

for

Noncovalent Interaction Origins of Substrate Discrimination in LinB: A Combined QM/MM and SAPT Analysis

Agata Sowińska,[a,*1] Michał Rostkowski,[a] Agnieszka Dybała-Defratyka,[a] Agnieszka Krzemińska[b,*2]

[a] – Institute of Applied Radiation Chemistry, Faculty of Chemistry, Lodz University of Technology, Zeromskiego 114, 90-543 Lodz, Poland

[b] – Institute of Physics, Lodz University of Technology, Wolczanska 217/221, 93-005 Lodz, Poland

[1] *To whom correspondence should be addressed at agata.sowinska@p.lodz.pl*

[2] *To whom correspondence should be addressed at agnieszka.krzeminska-kowalska@p.lodz.pl*

Contents

Table S1. Values of the CVs (Å) corresponding to the stationary points of the dehalogenation step in the LinB-catalysed reactions with DBP and DCP, reported according to the ligand configuration and the position of nucleophilic attack. 4

Table S2. Cluster analysis results for the dehalogenation step of the (S)-DBP pathways.......... 5

Table S3. Cluster analysis results for the dehalogenation step of the (R)-DBP pathways. 6

Table S4. Cluster analysis results for the dehalogenation step of the (S)-DCP pathways.......... 7

Table S5. Cluster analysis results for the dehalogenation step of the (R)-DCP pathways. 8

Table S6. Cartesian coordinates (Å) of the LinB first-shell amino acids in complex with the (S)-DBP, shown for the representative structure of the largest cluster corresponding to the stationary points of the dehalogenation step. 10

Table S7. Cartesian coordinates (Å) of the LinB first-shell amino acids in complex with the (R)-DBP, shown for the representative structure of the largest cluster corresponding to the stationary points of the dehalogenation step. 19

Table S8. Cartesian coordinates (Å) of the LinB first-shell amino acids in complex with the (S)-DCP, shown for the representative structure of the largest cluster corresponding to the stationary points of the dehalogenation step. 29

Table S9. Cartesian coordinates (Å) of the LinB first-shell amino acids in complex with the (R)-DCP, shown for the representative structure of the largest cluster corresponding to the stationary points of the dehalogenation step. 39

Figure S1. Free energy profile and evolution of the main CVs along the MFEP obtained for the LinB reaction with (S)-1,2-DBP in the C1 pathway. 49

Figure S2. Free energy profile and evolution of the main CVs along the MFEP obtained for the LinB reaction with (S)-1,2-DBP in the C2 pathway. 50

Figure S3. Free energy profile and evolution of the main CVs along the MFEP obtained for the LinB reaction with (R)-1,2-DBP in the C1 pathway. 51

Figure S4. Free energy profile and evolution of the main CVs along the MFEP obtained for the LinB reaction with (R)-1,2-DBP in the C2 pathway. 52

Figure S5. Free energy profile and evolution of the main CVs along the MFEP obtained for the LinB reaction with (S)-1,2-DCP in the C1 pathway. 53

Figure S6. Free energy profile and evolution of the main CVs along the MFEP obtained for the LinB reaction with (S)-1,2-DCP in the C2 pathway. 54

Figure S7. Free energy profile and evolution of the main CVs along the MFEP obtained for the LinB reaction with (R)-1,2-DCP in the C1 pathway. 55

Figure S8. Free energy profile and evolution of the main CVs along the MFEP obtained for the LinB reaction with (R)-1,2-DCP in the C2 pathway. 56

Table S10. SAPT2+ interaction energy decomposition for the selected amino acid-DBP complexes, with the corresponding standard errors of the mean (SEM). 57

Table S11. SAPT2+ interaction energy decomposition for the selected amino acid-DCP complexes, with the corresponding standard errors of the mean (SEM). 60

Figure S9. Representative structures of DBP in the (S)-C1 and (S)-C2 reactant complexes [RC] and their corresponding transition states [TS1]. 63

Figure S10. Representative structures of DBP in the (R)-C1 and (R)-C2 reactant complexes [RC] and their corresponding transition states [TS1]. 63

Figure S11. Representative structures of DCP in the (S)-C1 and (S)-C2 reactant complexes [RC] and their corresponding transition states [TS1]. 64

Figure S12. Representative structures of DCP in the (R)-C1 and (R)-C2 reactant complexes [RC] and their corresponding transition states [TS1]. 64

Table S12. Mean ligand–residue distances (Å) for representative structures obtained from cluster analysis, reported by ligand configuration and nucleophilic attack position, with standard errors of the mean (SEM). 65

Figure S13. Weak intermolecular interaction maps for the remaining stationary structures in the LinB active site. 66

Figure S14. Free energy profiles for each CV as a function of the reaction path for LinB-DBP reactions depending on the ligand configuration and the position of the nucleophilic attack. .67

References..67

Table S1. Values of the CVs (Å) corresponding to the reactant complexes [RC] and the first transition-state structures [TS1] of the dehalogenation step in the LinB-catalysed reactions with DBP and DCP, reported according to the ligand configuration and the position of nucleophilic attack.[3]

				C*X*-Br*X*	C*X*-OD1	OD1-CG	CG-O_w
DBP	*S*	C1	RC	1.99	3.20	1.26	3.47
			TS1	2.42	2.11	1.30	3.57
		C2	RC	2.00	3.42	1.26	3.54
			TS1	2.45	1.98	1.30	3.64
	R	C1	RC	1.97	3.23	1.25	3.38
			TS1	2.30	2.09	1.29	4.00
		C2	RC	1.96	2.96	1.26	3.51
			TS1	2.45	2.08	1.26	3.90
				C*X*-Cl*X*	C*X*-OD1	OD1-CG	CG-O_w
DCP	*S*	C1	RC	1.81	3.34	1.25	3.45
			TS1	2.26	1.98	1.29	3.80
		C2	RC	1.79	2.84	1.26	3.49
			TS1	2.37	1.89	1.30	3.49
	R	C1	RC	1.83	2.77	1.26	3.68
			TS1	2.23	1.95	1.29	3.81
		C2	RC	1.83	2.89	1.27	3.52
			TS1	2.46	2.04	1.27	3.52

[3] *CX, BrX, and ClX denote the atoms forming the respective carbon-halogen bond at the reactive position.*

Table S2. Cluster analysis results for the dehalogenation step of the (*S*)-DBP pathways. The analysed structures were selected based on all four CVs within ±0.05 Å of each stationary point. Highlighted clusters (including ≥ 5% of the population) provided the representative structures used for further analysis.[4]

C1												
	RC						TS1					
Cluster	Count	Frac	AvgDist	Stdev	Centroid	AvgCDist	Count	Frac	AvgDist	Stdev	Centroid	AvgCDist
0	264	0.41	0.71	0.11	259	0.91	151	0.65	0.59	0.07	137	0.78
1	171	0.27	0.67	0.11	74	0.99	30	0.13	0.59	0.10	109	0.84
2	140	0.22	0.70	0.12	329	0.97	24	0.10	0.55	0.08	20	0.77
3	42	0.07	0.67	0.13	608	0.96	11	0.05	0.54	0.13	189	0.81
4	10	0.02	0.48	0.07	637	1.06	6	0.03	0.51	0.07	229	0.88
5	4	0.01	0.48	0.13	176	1.00	4	0.02	0.51	0.10	201	0.83
6	4	0.01	0.34	0.06	553	1.02	2	0.01	0.60	0.00	119	0.85
7	3	0.01	0.58	0.01	574	1.04	2	0.01	0.48	0.00	225	0.85
8	1	0.00	0.00	0.00	400	1.00	1	0.00	0.00	0.00	116	0.84
9	1	0.00	0.00	0.00	549	1.03	1	0.00	0.00	0.00	117	0.85
C2												
	RC						TS1					
Cluster	Count	Frac	AvgDist	Stdev	Centroid	AvgCDist	Count	Frac	AvgDist	Stdev	Centroid	AvgCDist
0	231	0.44	0.68	0.09	265	1.00	104	0.69	0.62	0.08	29	0.77
1	104	0.20	0.65	0.10	40	0.88	10	0.07	0.59	0.09	63	0.80
2	62	0.12	0.57	0.09	324	1.00	10	0.07	0.49	0.10	18	0.78
3	30	0.06	0.68	0.11	125	0.90	9	0.06	0.57	0.09	7	0.85
4	28	0.05	0.61	0.09	409	0.92	5	0.03	0.55	0.05	52	0.81
5	24	0.05	0.59	0.12	517	0.94	5	0.03	0.55	0.03	148	0.88

[4] *Count – number of structures in cluster. Frac – size of cluster as fraction of total number of structures obtained for each stationary point. AvgDist – average distance between points in the cluster. Stdev – standard deviation of points in the cluster. Centroid – Frame # of structure in cluster that has the lowest cumulative distance to every other point. AvgCDist – average distance of this cluster to every other cluster.*

6	23	0.04	0.55	0.08	145	0.93	3	0.02	0.55	0.16	23	0.78
7	10	0.02	0.58	0.13	497	0.95	3	0.02	0.57	0.04	144	0.89
8	6	0.01	0.56	0.12	158	0.98	1	0.01	0.00	0.00	56	0.79
9	3	0.01	0.48	0.11	160	0.97	1	0.01	0.00	0.00	66	0.76

Table S3. Cluster analysis results for the dehalogenation step of the (*R*)-DBP pathways. The analysed structures were selected based on all four CVs within ±0.05 Å of each stationary point. Highlighted clusters (including ≥ 5% of the population) provided the representative structures used for further analysis.[5]

**C1**												
	**RC**						**TS1**					
Cluster	Count	Frac	AvgDist	Stdev	Centroid	AvgCDist	Count	Frac	AvgDist	Stdev	Centroid	AvgCDist
0	720	0.72	0.71	0.09	649	0.87	166	0.69	0.62	0.09	140	0.93
1	74	0.07	0.67	0.11	782	0.91	47	0.20	0.60	0.10	17	0.98
2	73	0.07	0.63	0.12	53	0.96	7	0.03	0.52	0.14	237	1.11
3	30	0.03	0.57	0.12	744	0.89	6	0.03	0.48	0.12	233	1.00
4	29	0.03	0.64	0.13	705	0.92	4	0.02	0.53	0.06	4	0.96
5	24	0.02	0.59	0.11	594	0.87	4	0.02	0.49	0.10	226	1.05
6	17	0.02	0.54	0.15	989	0.96	2	0.01	0.51	0.00	53	0.92
7	14	0.01	0.55	0.09	941	1.05	2	0.01	0.34	0.00	55	0.92
8	11	0.01	0.57	0.08	409	0.90	1	0.00	0.00	0.00	5	0.98
9	3	0.00	0.50	0.15	399	0.93	1	0.00	0.00	0.00	57	0.91
**C2**												
	**RC**						**TS1**					
Cluster	Count	Frac	AvgDist	Stdev	Centroid	AvgCDist	Count	Frac	AvgDist	Stdev	Centroid	AvgCDist

[5] *Count – number of structures in cluster. Frac – size of cluster as fraction of total number of structures obtained for each stationary point. AvgDist – average distance between points in the cluster. Stdev – standard deviation of points in the cluster. Centroid – Frame # of structure in cluster that has the lowest cumulative distance to every other point. AvgCDist – average distance of this cluster to every other cluster.*

0	946	0.69	0.79	0.12	20	1.00	105	0.31	0.62	0.10	43	1.01
1	150	0.11	0.75	0.14	365	1.01	64	0.19	0.67	0.10	285	1.07
2	111	0.08	0.70	0.11	764	1.04	62	0.18	0.61	0.10	203	0.90
3	53	0.04	0.76	0.16	1167	1.01	42	0.12	0.61	0.08	328	0.97
4	49	0.04	0.66	0.11	158	0.99	36	0.11	0.60	0.10	171	0.93
5	31	0.02	0.59	0.10	1360	1.04	12	0.04	0.70	0.12	108	1.02
6	17	0.01	0.66	0.13	211	1.01	7	0.02	0.62	0.11	130	0.97
7	14	0.01	0.52	0.12	1109	1.09	7	0.02	0.60	0.10	146	0.92
8	7	0.01	0.73	0.11	1376	1.04	3	0.01	0.59	0.07	152	1.01
9	2	0.00	0.75	0.00	725	1.05	2	0.01	0.46	0.00	136	0.92

Table S4. Cluster analysis results for the dehalogenation step of the (*S*)-DCP pathways. The analysed structures were selected based on all four CVs within ±0.05 Å of each stationary point. Highlighted clusters (including ≥ 5% of the population) provided the representative structures used for further analysis.[6]

**C1**												
	**RC**						**TS1**					
Cluster	Count	Frac	AvgDist	Stdev	Centroid	AvgCDist	Count	Frac	AvgDist	Stdev	Centroid	AvgCDist
0	362	0.83	0.60	0.07	395	0.68	115	0.87	0.56	0.07	24	0.66
1	23	0.05	0.53	0.07	103	0.70	4	0.03	0.48	0.12	86	0.73
2	15	0.04	0.52	0.09	293	0.76	3	0.02	0.41	0.15	63	0.75
3	11	0.03	0.58	0.09	28	0.72	2	0.02	0.53	0.00	38	0.75
4	6	0.01	0.48	0.18	140	0.76	2	0.02	0.51	0.00	72	0.74
5	5	0.01	0.47	0.12	87	0.79	2	0.02	0.55	0.00	110	0.72
6	4	0.01	0.56	0.03	148	0.76	1	0.01	0.00	0.00	14	0.69
7	4	0.01	0.51	0.04	198	0.76	1	0.01	0.00	0.00	62	0.77

[6] *Count – number of structures in cluster. Frac – size of cluster as fraction of total number of structures obtained for each stationary point. AvgDist – average distance between points in the cluster. Stdev – standard deviation of points in the cluster. Centroid – Frame # of structure in cluster that has the lowest cumulative distance to every other point. AvgCDist – average distance of this cluster to every other cluster.*

8	3	0.01	0.43	0.07	248	0.77	1	0.01	0.00	0.00	111	0.70
9	1	0.00	0.00	0.00	49	0.72	1	0.01	0.00	0.00	126	0.76
**C2**												
	**RC**						**TS1**					
Cluster	Count	Frac	AvgDist	Stdev	Centroid	AvgCDist	Count	Frac	AvgDist	Stdev	Centroid	AvgCDist
0	532	0.83	0.57	0.07	9	0.64	278	0.80	0.57	0.06	208	0.65
1	78	0.12	0.54	0.07	149	0.69	35	0.10	0.54	0.07	58	0.67
2	9	0.01	0.52	0.09	246	0.71	8	0.02	0.52	0.10	39	0.72
3	5	0.01	0.50	0.11	102	0.68	8	0.02	0.56	0.05	236	0.67
4	4	0.01	0.53	0.11	216	0.69	7	0.02	0.54	0.13	245	0.72
5	4	0.01	0.36	0.11	325	0.68	6	0.02	0.42	0.14	91	0.67
6	2	0.00	0.56	0.00	334	0.68	3	0.01	0.45	0.12	275	0.70
7	2	0.00	0.57	0.00	575	0.69	2	0.01	0.50	0.00	338	0.71
8	1	0.00	0.00	0.00	351	0.68	1	0.00	0.00	0.00	93	0.73
9	1	0.00	0.00	0.00	558	0.73	1	0.00	0.00	0.00	238	0.67

Table S5. Cluster analysis results for the dehalogenation step of the (*R*)-DCP pathways. The analysed structures were selected based on all four CVs within ±0.05 Å of each stationary point. Highlighted clusters (including ≥ 5% of the population) provided the representative structures used for further analysis.[7]

**C1**												
	**RC**						**TS1**					
Cluster	Count	Frac	AvgDist	Stdev	Centroid	AvgCDist	Count	Frac	AvgDist	Stdev	Centroid	AvgCDist
0	82	0.34	0.59	0.08	109	0.77	108	0.54	0.55	0.08	53	0.70
1	56	0.23	0.61	0.09	16	0.77	34	0.17	0.53	0.07	166	0.65

[7] *Count – number of structures in cluster. Frac – size of cluster as fraction of total number of structures obtained for each stationary point. AvgDist – average distance between points in the cluster. Stdev – standard deviation of points in the cluster. Centroid – Frame # of structure in cluster that has the lowest cumulative distance to every other point. AvgCDist – average distance of this cluster to every other cluster.*

2	37	0.16	0.56	0.08	80	0.78	14	0.07	0.51	0.07	199	0.78
3	26	0.11	0.54	0.09	172	0.77	13	0.07	0.53	0.08	182	0.75
4	12	0.05	0.55	0.09	185	0.83	10	0.05	0.50	0.10	123	0.69
5	12	0.05	0.57	0.09	204	0.82	7	0.04	0.50	0.04	134	0.69
6	9	0.04	0.49	0.09	228	0.88	5	0.03	0.47	0.13	4	0.76
7	2	0.01	0.56	0.00	21	0.79	4	0.02	0.50	0.05	156	0.69
8	2	0.01	0.50	0.00	218	0.88	4	0.02	0.51	0.04	171	0.72
9	1	0.00	0.00	0.00	58	0.92	2	0.01	0.51	0.00	148	0.66
**C2**												
	**RC**						**TS1**					
Cluster	Count	Frac	AvgDist	Stdev	Centroid	AvgCDist	Count	Frac	AvgDist	Stdev	Centroid	AvgCDist
0	523	0.70	0.66	0.10	9	0.79	169	0.39	0.57	0.07	37	0.81
1	132	0.18	0.59	0.09	484	0.82	147	0.34	0.56	0.07	285	0.73
2	39	0.05	0.61	0.10	704	0.82	41	0.10	0.52	0.07	231	0.76
3	30	0.04	0.54	0.08	214	0.85	38	0.09	0.55	0.08	254	0.76
4	19	0.03	0.58	0.11	81	0.82	12	0.03	0.53	0.08	180	0.80
5	4	0.01	0.46	0.08	671	0.82	8	0.02	0.43	0.10	27	0.75
6	2	0.00	0.52	0.00	748	0.84	6	0.01	0.52	0.11	309	0.75
7	1	0.00	0.00	0.00	44	0.83	5	0.01	0.49	0.08	246	0.79
8	1	0.00	0.00	0.00	125	0.89	4	0.01	0.40	0.09	189	0.91
9	1	0.00	0.00	0.00	670	0.91	1	0.00	0.00	0.00	380	0.76

Table S6. Cartesian coordinates (Å) of the LinB first-shell amino acids in complex with the (*S*)-DBP, shown for the representative structure of the largest cluster corresponding to the stationary points of the dehalogenation step. Provided geometries were used as the reference model for SAPT analysis of noncovalent interactions.

**C1**								**C2**							
**RC**				**TS1**				**RC**				**TS1**			
Atom	X	Y	Z	Atom	X	Y	Z	Atom	X	Y	Z	Atom	X	Y	Z
N	34.220	32.005	37.256	N	34.490	31.763	37.022	N	34.524	31.921	36.984	N	34.062	32.188	37.024
H	34.667	32.346	38.151	H	34.914	31.921	37.917	H	34.954	31.926	37.875	H	34.319	32.389	38.015
C	32.887	31.448	37.196	C	33.186	31.223	36.901	C	33.275	31.272	36.779	C	32.911	31.412	36.840
H	32.920	30.720	36.302	H	33.189	30.484	36.069	H	33.306	30.661	35.940	H	33.093	30.781	36.000
C	32.780	30.606	38.495	C	32.921	30.554	38.281	C	33.001	30.430	38.052	C	32.824	30.572	38.158
H	33.483	29.794	38.606	H	33.751	29.875	38.318	H	33.878	29.742	38.112	H	33.728	30.157	38.529
H	33.044	31.215	39.357	H	32.890	31.283	39.144	H	32.883	31.008	38.932	H	32.467	31.257	38.998
C	31.422	30.026	38.526	C	31.672	29.712	38.285	C	31.835	29.508	38.021	C	31.819	29.372	38.069
O	31.155	29.000	37.832	O	31.275	29.121	37.272	O	31.677	28.708	37.137	O	31.900	28.606	37.137
N	30.482	30.646	39.190	N	30.972	29.483	39.353	N	30.878	29.409	38.905	N	30.880	29.269	38.966
H	29.517	30.224	39.142	H	30.443	28.603	39.333	H	30.266	28.622	38.847	H	30.209	28.528	38.962
H	30.645	31.585	39.583	H	31.339	29.995	40.195	H	30.678	30.000	39.729	H	30.733	29.983	39.663
C	31.734	32.432	36.978	C	32.097	32.322	36.551	C	31.997	32.176	36.620	C	31.650	32.294	36.640
O	31.918	33.603	37.415	O	32.123	33.331	37.184	O	31.978	33.273	37.128	O	31.516	33.309	37.278
N	37.535	37.333	39.371	N	37.649	37.243	39.560	N	37.608	37.278	39.324	N	37.580	37.465	39.645
H	37.165	38.232	39.651	H	37.460	38.093	40.113	H	37.502	38.160	39.799	H	37.154	38.356	39.907
C	36.859	36.241	40.043	C	37.084	35.999	40.195	C	36.992	36.137	40.018	C	36.963	36.235	40.239
H	36.477	36.692	40.916	H	36.664	36.332	41.079	H	36.877	36.372	41.028	H	36.556	36.485	41.195
C	35.562	35.922	39.304	C	35.890	35.459	39.271	C	35.608	35.835	39.230	C	35.693	35.867	39.370
H	35.014	36.822	39.305	H	35.190	36.253	39.044	H	35.029	36.739	39.202	H	35.064	36.751	39.310
H	35.796	35.736	38.317	H	36.346	35.242	38.344	H	35.905	35.625	38.236	H	36.038	35.698	38.410
C	34.727	34.864	39.931	C	35.069	34.367	39.912	C	34.778	34.712	39.810	C	34.938	34.777	39.975

O	33.855	35.248	40.719	O	34.474	34.523	41.056	O	34.039	35.034	40.794	O	34.700	34.969	41.249
O	34.828	33.646	39.611	O	35.030	33.234	39.369	O	34.793	33.582	39.297	O	34.892	33.616	39.531
C	37.769	35.027	40.407	C	38.153	34.907	40.512	C	38.078	34.977	40.151	C	38.033	35.120	40.553
O	38.826	35.222	40.999	O	39.288	35.224	40.882	O	39.178	35.263	40.657	O	39.060	35.415	41.092
N	37.520	33.824	39.941	N	37.898	33.591	40.223	N	37.791	33.722	39.788	N	37.784	33.843	40.215
H	36.626	33.632	39.458	H	36.972	33.344	39.893	H	36.832	33.635	39.396	H	36.900	33.621	39.988
C	38.462	32.688	40.127	C	38.835	32.504	40.193	C	38.698	32.588	39.815	C	38.774	32.765	40.129
H	38.883	32.584	41.133	H	39.306	32.413	41.205	H	39.076	32.530	40.824	H	39.251	32.562	41.157
C	37.761	31.365	39.874	C	38.120	31.215	39.812	C	37.916	31.253	39.639	C	38.089	31.485	39.827
H	37.019	31.409	39.042	H	37.493	31.305	38.956	H	37.379	31.249	38.650	H	37.261	31.681	39.084
H	38.542	30.777	39.447	H	38.872	30.503	39.511	H	38.658	30.505	39.416	H	38.820	30.769	39.363
C	37.055	30.666	41.030	C	37.411	30.657	41.007	C	37.221	30.683	40.858	C	37.466	30.866	41.031
C	35.715	30.647	41.259	C	36.069	30.465	41.085	C	35.929	30.697	41.137	C	36.147	30.814	41.277
H	34.989	31.150	40.642	H	35.384	30.686	40.254	H	35.127	31.167	40.508	H	35.302	31.095	40.661
N	35.462	30.165	42.479	N	35.726	29.955	42.281	N	35.727	30.151	42.318	N	36.000	30.224	42.556
H	34.469	30.177	42.861	H	34.772	29.615	42.547	H	34.812	29.953	42.723	H	35.062	30.107	42.948
C	36.604	29.582	43.020	C	36.860	29.614	43.015	C	36.945	29.696	42.897	C	37.178	29.832	43.140
C	36.766	28.851	44.209	C	37.130	28.898	44.214	C	37.361	28.955	44.034	C	37.434	29.031	44.256
H	36.003	28.579	44.953	H	36.341	28.347	44.642	H	36.557	28.498	44.593	H	36.625	28.498	44.769
C	38.086	28.432	44.519	C	38.441	28.768	44.722	C	38.677	28.775	44.394	C	38.787	28.959	44.640
H	38.165	27.906	45.483	H	38.556	28.105	45.532	H	39.010	28.324	45.307	H	38.989	28.307	45.488
C	39.175	28.799	43.725	C	39.493	29.319	43.987	C	39.650	29.215	43.532	C	39.810	29.521	43.844
H	40.174	28.533	44.137	H	40.474	28.987	44.406	H	40.625	29.205	43.853	H	40.814	29.317	44.187
C	38.917	29.560	42.564	C	39.259	29.995	42.774	C	39.247	29.832	42.281	C	39.434	30.264	42.632
H	39.700	29.692	41.843	H	40.093	30.265	42.130	H	40.033	30.095	41.606	H	40.298	30.492	42.040
C	37.648	29.942	42.143	C	37.979	30.141	42.257	C	37.898	30.087	41.973	C	38.102	30.305	42.222
C	39.765	32.874	39.221	C	40.059	32.690	39.295	C	39.866	32.624	38.879	C	39.986	33.012	39.210
O	40.878	32.443	39.606	O	41.143	32.210	39.677	O	40.972	32.076	39.104	O	40.987	32.325	39.426

N	37.220	42.568	41.372	N	37.565	42.257	41.885	N	37.458	42.297	41.554	N	37.654	42.545	41.798
H	37.121	43.462	41.874	H	37.464	43.243	42.100	H	37.334	43.286	41.758	H	37.919	43.381	42.342
C	36.389	41.477	41.886	C	36.730	41.273	42.571	C	36.563	41.309	42.292	C	36.798	41.525	42.424
H	36.109	41.620	42.922	H	36.422	41.651	43.554	H	36.433	41.715	43.242	H	36.603	41.884	43.486
C	35.070	41.434	41.170	C	35.435	41.085	41.805	C	35.146	41.150	41.612	C	35.358	41.426	41.853
H	35.328	41.411	40.128	H	35.604	40.701	40.793	H	35.313	41.036	40.541	H	35.489	41.441	40.745
H	34.406	40.543	41.396	H	34.984	40.219	42.261	H	34.709	40.262	42.013	H	34.858	40.495	42.015
C	34.228	42.689	41.479	C	34.400	42.240	41.871	C	34.144	42.290	41.779	C	34.362	42.604	41.972
H	34.849	43.602	41.533	H	34.935	43.181	41.660	H	34.699	43.205	41.700	H	34.945	43.551	41.977
H	33.619	42.869	40.531	H	33.697	42.156	41.045	H	33.288	42.149	41.077	H	33.747	42.580	41.001
C	33.432	42.533	42.750	C	33.525	42.115	43.097	C	33.709	42.369	43.234	C	33.546	42.366	43.162
O	33.765	43.128	43.837	O	33.916	42.473	44.246	O	34.419	42.689	44.205	O	33.971	42.760	44.321
O	32.286	42.027	42.679	O	32.411	41.564	43.046	O	32.493	42.243	43.600	O	32.366	41.930	43.194
C	37.135	40.154	42.102	C	37.442	39.861	42.800	C	37.212	39.927	42.607	C	37.460	40.145	42.608
O	36.735	39.131	41.550	O	36.983	38.798	42.367	O	36.815	38.933	42.013	O	36.967	39.120	42.108
N	39.412	37.170	44.541	N	39.952	36.997	44.768	N	39.612	36.931	44.650	N	39.865	37.065	44.771
H	39.719	36.693	43.693	H	39.983	36.493	43.952	H	39.898	36.602	43.733	H	40.229	36.666	43.894
C	39.525	36.281	45.738	C	39.861	36.219	46.033	C	39.644	35.927	45.795	C	39.851	36.218	45.988
H	38.639	36.377	46.374	H	38.888	36.477	46.490	H	38.877	36.141	46.552	H	39.029	36.635	46.555
C	39.428	34.838	45.261	C	39.662	34.692	45.758	C	39.454	34.496	45.270	C	39.503	34.675	45.829
H	40.205	34.650	44.567	H	40.682	34.336	45.430	H	40.374	34.177	44.882	H	40.343	34.142	45.302
C	39.429	33.830	46.424	C	39.299	33.861	46.980	C	39.155	33.615	46.494	C	39.254	34.056	47.276
H	39.344	32.834	46.079	H	39.503	32.855	46.732	H	38.088	33.635	46.630	H	39.265	33.023	47.167
H	40.231	33.813	47.185	H	39.928	34.162	47.776	H	39.354	32.599	46.176	H	40.030	34.261	48.010
H	38.524	33.910	47.026	H	38.294	34.116	47.308	H	39.852	33.859	47.345	H	38.340	34.472	47.623
C	38.114	34.532	44.480	C	38.650	34.423	44.587	C	38.365	34.439	44.166	C	38.252	34.524	44.995
H	37.327	34.811	45.092	H	37.633	34.822	44.877	H	37.325	34.682	44.443	H	37.364	34.783	45.552
H	37.994	35.276	43.692	H	38.998	34.932	43.676	H	38.637	35.061	43.354	H	38.405	35.157	44.116

C	37.782	33.166	43.918	C	38.401	32.949	44.281	C	38.171	33.020	43.570	C	38.063	33.057	44.660
H	37.610	32.523	44.741	H	37.879	32.914	43.372	H	37.560	33.115	42.668	H	37.345	33.034	43.849
H	37.029	33.078	43.112	H	39.309	32.437	44.099	H	39.142	32.457	43.451	H	38.906	32.611	44.187
H	38.686	32.736	43.526	H	37.762	32.532	45.064	H	37.553	32.434	44.224	H	37.557	32.392	45.444
C	40.863	36.560	46.426	C	41.079	36.430	46.909	C	41.049	36.049	46.455	C	41.135	36.524	46.824
O	41.739	35.717	46.360	O	41.966	35.618	46.999	O	41.862	35.111	46.462	O	42.008	35.660	46.950
N	29.834	38.250	53.406	N	29.208	37.209	53.189	N	29.417	37.453	53.960	N	28.961	37.582	53.499
H	30.261	37.598	53.994	H	29.415	36.452	53.852	H	29.953	36.639	54.325	H	29.398	36.878	54.049
C	29.250	37.856	52.113	C	28.719	36.861	51.849	C	28.928	37.356	52.593	C	28.395	37.078	52.244
H	29.828	38.399	51.433	H	29.398	37.444	51.198	H	29.503	38.076	52.092	H	28.921	37.623	51.479
C	29.401	36.367	51.812	C	28.975	35.364	51.611	C	29.241	35.956	51.962	C	28.651	35.579	52.047
H	30.462	36.059	51.868	H	30.047	35.154	51.914	H	30.282	35.646	52.188	H	29.611	35.216	52.511
H	28.810	35.759	52.619	H	28.315	34.750	52.229	H	28.507	35.327	52.442	H	27.905	34.976	52.556
C	29.037	35.829	50.426	C	28.693	34.865	50.170	C	29.151	35.955	50.392	C	28.742	35.274	50.566
C	30.096	35.289	49.590	C	29.726	34.775	49.246	C	30.216	36.636	49.711	C	29.989	35.433	49.920
H	31.142	35.553	49.800	H	30.785	34.826	49.560	H	31.083	36.976	50.331	H	30.899	35.657	50.508
C	29.713	34.616	48.406	C	29.454	34.400	47.911	C	30.377	36.546	48.326	C	30.087	35.056	48.531
H	30.417	34.166	47.732	H	30.211	34.214	47.144	H	31.203	36.978	47.719	H	31.032	35.171	47.972
C	28.327	34.586	48.009	C	28.107	34.115	47.556	C	29.487	35.644	47.729	C	28.957	34.594	47.773
H	28.018	34.047	47.087	H	27.873	33.946	46.517	H	29.577	35.531	46.661	H	29.163	34.284	46.731
C	27.325	35.165	48.785	C	27.111	34.228	48.526	C	28.351	35.057	48.378	C	27.734	34.463	48.502
H	26.300	35.085	48.535	H	26.101	34.062	48.279	H	27.624	34.530	47.734	H	26.868	34.140	47.987
C	27.695	35.766	50.026	C	27.392	34.583	49.860	C	28.192	35.254	49.746	C	27.632	34.806	49.887
H	26.909	36.036	50.723	H	26.652	34.842	50.587	H	27.426	34.741	50.350	H	26.690	34.683	50.411
C	27.761	38.228	52.065	C	27.242	37.219	51.608	C	27.441	37.744	52.590	C	26.803	37.505	52.223
O	27.062	37.998	53.069	O	26.387	36.896	52.469	O	26.671	37.205	53.413	O	26.063	37.319	53.233
N	23.898	30.463	47.443	N	23.674	29.881	47.252	N	23.998	30.654	47.373	N	24.079	29.443	47.699
H	23.727	31.410	47.724	H	23.561	30.816	47.611	H	24.083	31.541	47.758	H	23.844	30.234	48.284

C	25.205	29.871	47.476	C	25.036	29.308	47.161	C	25.212	29.818	47.320	C	25.371	28.836	47.705
H	25.392	29.348	46.563	H	24.998	28.774	46.191	H	25.295	29.407	46.306	H	25.485	28.350	46.732
C	26.172	31.104	47.617	C	26.166	30.456	47.047	C	26.390	30.614	47.694	C	26.447	29.924	47.905
H	25.755	31.838	48.316	H	25.940	31.208	47.786	H	26.178	31.431	48.434	H	26.088	30.685	48.546
H	27.132	30.750	47.970	H	27.106	29.974	47.436	H	27.164	29.990	48.229	H	27.336	29.418	48.337
C	26.392	31.742	46.293	C	26.385	31.068	45.702	C	27.038	31.382	46.632	C	26.932	30.634	46.673
C	25.500	32.653	45.712	C	25.329	31.870	45.179	C	26.382	32.334	45.825	C	26.030	31.424	45.990
H	24.618	32.992	46.210	H	24.355	32.006	45.713	H	25.326	32.524	45.859	H	25.012	31.414	46.333
C	25.784	33.151	44.398	C	25.498	32.555	44.002	C	27.105	33.009	44.742	C	26.436	32.252	44.915
H	25.083	33.834	43.953	H	24.779	33.190	43.610	H	26.723	33.819	44.136	H	25.772	32.879	44.318
C	26.943	32.748	43.713	C	26.715	32.495	43.315	C	28.481	32.732	44.547	C	27.738	32.112	44.441
H	27.226	33.126	42.737	H	26.942	33.038	42.454	H	29.025	33.338	43.738	H	28.036	32.835	43.693
C	27.858	31.897	44.346	C	27.724	31.654	43.822	C	29.208	31.842	45.362	C	28.697	31.331	45.145
H	28.813	31.659	43.899	H	28.635	31.504	43.184	H	30.243	31.674	45.123	H	29.759	31.358	44.842
C	27.550	31.345	45.603	C	27.673	30.957	45.023	C	28.424	31.251	46.419	C	28.331	30.635	46.305
H	28.228	30.702	46.076	H	28.515	30.337	45.343	H	28.967	30.636	47.202	H	29.109	30.183	46.887
C	25.346	28.797	48.520	C	25.398	28.185	48.217	C	25.092	28.628	48.279	C	25.478	27.650	48.625
O	26.026	27.747	48.390	O	26.048	27.211	47.839	O	25.664	27.597	48.003	O	26.076	26.612	48.258
N	22.293	29.461	38.140	N	22.640	28.886	37.593	N	21.937	28.813	38.003	N	23.049	28.547	37.950
H	22.351	28.450	38.147	H	22.753	27.891	37.460	H	22.174	27.888	37.728	H	23.332	27.582	37.854
C	23.441	30.305	37.894	C	23.791	29.798	37.689	C	22.977	29.857	37.903	C	24.144	29.525	38.004
H	23.477	31.063	38.739	H	23.542	30.415	38.588	H	22.811	30.619	38.725	H	23.893	30.196	38.767
C	24.676	29.433	38.058	C	25.045	28.972	38.057	C	24.389	29.247	38.041	C	25.437	28.843	38.406
H	24.428	28.432	38.447	H	24.712	28.202	38.718	H	24.317	28.274	38.486	H	25.126	27.920	38.962
H	25.163	29.302	37.069	H	25.409	28.428	37.227	H	24.766	29.124	36.993	H	25.901	28.649	37.447
C	25.737	29.947	39.005	C	26.130	29.819	38.733	C	25.395	30.006	38.852	C	26.404	29.687	39.228
C	26.536	29.110	39.818	C	26.534	29.422	40.013	C	25.704	29.559	40.143	C	26.857	29.223	40.468
H	26.407	28.052	39.743	H	26.046	28.639	40.498	H	25.099	28.833	40.636	H	26.403	28.387	40.873

C	27.556	29.608	40.621	C	27.568	30.106	40.675	C	26.664	30.230	40.962	C	27.865	29.898	41.164
H	28.139	28.939	41.249	H	27.872	29.811	41.701	H	26.892	29.825	41.944	H	28.296	29.417	42.007
C	27.767	31.040	40.567	C	28.168	31.304	40.075	C	27.368	31.310	40.447	C	28.370	31.126	40.708
H	28.456	31.608	41.265	H	28.910	31.872	40.596	H	28.102	31.895	41.011	H	29.143	31.611	41.241
C	27.075	31.905	39.672	C	27.736	31.662	38.798	C	26.969	31.762	39.133	C	27.816	31.697	39.566
H	27.276	32.957	39.770	H	28.171	32.563	38.402	H	27.477	32.638	38.770	H	28.275	32.624	39.216
C	26.107	31.311	38.835	C	26.759	30.957	38.124	C	26.006	31.145	38.304	C	26.914	30.973	38.769
H	25.691	31.788	38.011	H	26.384	31.373	37.165	H	25.782	31.553	37.335	H	26.584	31.291	37.770
C	23.456	31.104	36.565	C	23.912	30.621	36.384	C	22.850	30.580	36.584	C	24.258	30.322	36.690
O	23.759	32.315	36.520	O	24.124	31.832	36.386	O	22.780	31.816	36.720	O	24.263	31.592	36.734
N	21.261	34.323	37.973	N	21.359	33.639	37.639	N	20.987	33.880	38.002	N	21.850	33.190	38.408
H	21.310	33.365	37.926	H	21.515	32.649	37.553	H	21.064	32.886	37.753	H	21.863	32.149	38.377
C	22.426	34.944	38.630	C	22.365	34.403	38.429	C	22.167	34.569	38.602	C	22.880	33.998	39.236
H	22.051	35.652	39.337	H	21.812	35.079	39.102	H	21.797	35.337	39.211	H	22.373	34.671	39.874
C	23.194	33.903	39.490	C	23.161	33.468	39.332	C	22.909	33.589	39.450	C	23.737	33.093	40.137
H	23.580	33.188	38.758	H	23.375	32.572	38.757	H	23.391	32.901	38.833	H	24.267	32.410	39.490
C	24.448	34.618	40.092	C	24.517	33.920	39.801	C	23.985	34.258	40.374	C	24.932	33.557	40.973
H	24.062	35.476	40.575	H	24.585	34.844	40.382	H	23.430	35.057	40.888	H	24.526	33.924	41.894
H	24.766	34.038	40.965	H	25.060	33.141	40.315	H	24.473	33.477	40.988	H	25.545	32.591	41.060
H	25.186	34.711	39.284	H	25.158	34.108	38.978	H	24.794	34.630	39.844	H	25.491	34.234	40.342
C	22.281	33.256	40.626	C	22.370	32.866	40.476	C	22.047	32.861	40.467	C	22.907	32.210	41.056
H	22.159	33.946	41.463	H	22.263	33.632	41.265	H	21.519	33.590	41.182	H	22.270	31.651	40.434
H	21.324	33.088	40.225	H	21.376	32.414	40.236	H	21.132	32.340	40.033	H	23.431	31.669	41.840
H	22.860	32.412	41.080	H	22.986	32.141	40.920	H	22.566	32.204	41.105	H	22.336	32.857	41.793
C	23.295	35.766	37.661	C	23.214	35.370	37.550	C	23.020	35.292	37.664	C	23.664	34.953	38.342
O	23.528	36.900	37.894	O	23.191	36.596	37.764	O	23.152	36.488	37.851	O	24.115	35.953	38.793
N	33.011	25.076	41.645	N	33.435	24.968	41.147	N	33.382	25.164	41.031	N	33.474	25.333	41.366
H	32.720	24.917	40.673	H	33.199	24.874	40.178	H	33.011	25.003	40.118	H	33.201	25.200	40.431

C	33.572	26.321	41.918	C	33.989	26.229	41.567	C	33.783	26.490	41.337	C	34.024	26.648	41.701
H	34.633	26.244	42.033	H	35.030	26.127	41.678	H	34.874	26.483	41.552	H	35.042	26.401	41.946
C	33.303	27.193	40.687	C	33.853	27.268	40.510	C	33.643	27.349	40.132	C	33.985	27.610	40.465
H	32.326	27.068	40.203	H	32.848	27.156	40.066	H	32.657	27.136	39.771	H	33.045	27.513	40.010
H	33.341	28.247	41.064	H	34.009	28.284	40.852	H	33.688	28.409	40.342	H	34.078	28.653	40.841
C	34.380	27.042	39.631	C	34.869	27.126	39.384	C	34.625	27.199	39.060	C	35.114	27.477	39.407
C	34.087	26.917	38.315	C	34.677	26.932	38.068	C	34.461	27.087	37.731	C	34.893	27.228	38.088
H	33.101	26.751	37.867	H	33.661	26.750	37.674	H	33.483	26.999	37.276	H	33.969	26.978	37.646
N	35.320	26.860	37.642	N	35.839	26.937	37.383	N	35.641	26.967	37.064	N	36.120	27.185	37.435
H	35.374	26.560	36.666	H	35.931	26.690	36.339	H	35.819	26.746	36.086	H	36.228	26.868	36.480
C	36.381	27.077	38.450	C	36.878	27.168	38.228	C	36.693	27.129	37.923	C	37.169	27.401	38.344
C	37.767	27.098	38.186	C	38.267	27.231	37.981	C	38.097	27.292	37.693	C	38.592	27.441	38.166
H	38.133	26.925	37.181	H	38.699	27.125	36.966	H	38.470	27.129	36.676	H	39.012	27.395	37.172
C	38.650	27.180	39.281	C	39.161	27.338	39.144	C	38.962	27.556	38.778	C	39.417	27.549	39.316
H	39.718	27.117	39.144	H	40.243	27.339	38.983	H	40.040	27.567	38.709	H	40.528	27.717	39.297
C	38.107	27.271	40.547	C	38.576	27.348	40.430	C	38.452	27.648	40.051	C	38.749	27.528	40.605
H	38.729	27.362	41.469	H	39.257	27.356	41.183	H	39.122	27.846	40.805	H	39.365	27.780	41.440
C	36.781	27.129	40.818	C	37.229	27.245	40.620	C	37.068	27.488	40.323	C	37.359	27.391	40.695
H	36.392	27.140	41.885	H	36.867	27.266	41.689	H	36.636	27.647	41.330	H	36.885	27.652	41.637
C	35.844	27.077	39.730	C	36.345	27.188	39.517	C	36.114	27.227	39.224	C	36.534	27.494	39.587
C	33.060	27.040	43.201	C	33.427	26.746	42.890	C	33.153	27.139	42.580	C	33.350	27.259	42.969
O	33.915	27.414	44.038	O	34.211	26.850	43.869	O	33.896	27.664	43.435	O	34.142	27.721	43.778
N	31.725	27.130	43.513	N	32.094	26.977	43.055	N	31.830	27.192	42.791	N	31.958	27.293	43.155
C	30.545	26.940	42.688	C	31.022	26.601	42.113	C	30.787	27.092	41.773	C	30.827	26.951	42.271
H	30.720	26.017	42.136	H	31.163	25.600	41.791	H	30.813	26.183	41.205	H	30.923	26.070	41.643
H	30.398	27.846	42.014	H	31.121	27.271	41.234	H	30.739	27.952	41.072	H	30.699	27.874	41.704
C	29.392	26.796	43.608	C	29.637	26.750	42.725	C	29.433	27.205	42.494	C	29.564	26.840	43.126
H	29.454	25.767	44.119	H	29.378	25.766	43.147	H	29.115	26.244	42.973	H	29.333	25.912	43.633

H	28.483	27.086	43.089	H	28.865	27.121	42.081	H	28.746	27.693	41.795	H	28.686	27.204	42.605
C	29.735	27.860	44.590	C	29.978	27.649	43.896	C	29.836	28.150	43.648	C	29.896	27.790	44.315
H	29.184	27.804	45.548	H	29.322	27.406	44.791	H	29.108	28.023	44.486	H	29.383	27.467	45.204
H	29.601	28.869	44.185	H	29.882	28.697	43.683	H	29.709	29.130	43.292	H	29.587	28.858	44.038
C	31.205	27.571	44.849	C	31.479	27.426	44.271	C	31.291	27.804	44.037	C	31.411	27.754	44.420
H	31.650	28.518	45.088	H	31.905	28.415	44.582	H	31.772	28.761	44.230	H	31.738	28.775	44.656
C	31.504	26.627	46.040	C	31.664	26.437	45.439	C	31.523	26.993	45.308	C	31.843	26.953	45.654
O	31.414	27.123	47.170	O	31.711	26.885	46.654	O	31.389	27.587	46.393	O	31.979	27.370	46.771
N	35.627	27.527	47.322	N	35.329	27.154	47.273	N	35.483	27.511	46.927	N	35.866	27.501	47.211
H	34.722	27.468	46.912	H	34.479	27.118	46.816	H	34.565	27.481	46.567	H	34.932	27.221	47.038
C	35.853	28.856	47.934	C	35.804	28.471	47.691	C	35.839	28.789	47.559	C	36.140	28.810	47.793
H	36.418	29.530	47.226	H	36.410	28.868	46.860	H	36.539	29.232	46.796	H	36.805	29.361	47.097
C	34.526	29.574	48.309	C	34.539	29.481	47.716	C	34.640	29.832	47.645	C	34.914	29.774	47.735
H	33.986	29.046	49.125	H	33.919	29.154	48.463	H	33.872	29.331	48.347	H	34.160	29.253	48.252
C	34.899	30.972	48.799	C	34.974	30.840	48.202	C	34.883	31.178	48.336	C	35.130	31.181	48.309
H	35.474	31.476	48.055	H	35.759	31.168	47.545	H	33.993	31.622	48.874	H	36.069	31.592	47.873
H	34.042	31.619	49.001	H	34.139	31.632	48.207	H	35.660	31.084	49.137	H	34.304	31.828	48.179
H	35.420	30.983	49.772	H	35.420	30.827	49.199	H	35.378	31.872	47.694	H	35.341	31.041	49.441
C	33.568	29.529	47.020	C	33.772	29.631	46.397	C	33.950	30.014	46.288	C	34.407	29.955	46.300
H	34.078	30.020	46.205	H	34.340	30.416	45.897	H	34.719	30.232	45.611	H	35.112	30.480	45.549
H	33.307	28.505	46.818	H	33.793	28.676	45.858	H	33.635	29.042	46.001	H	34.203	29.099	45.752
C	32.255	30.231	47.380	C	32.342	30.129	46.678	C	32.747	30.982	46.311	C	33.045	30.751	46.188
H	32.425	31.276	47.668	H	31.959	30.332	45.687	H	32.955	32.005	46.664	H	32.353	30.228	46.900
H	31.537	30.257	46.588	H	31.815	29.246	47.066	H	32.352	31.040	45.281	H	33.239	31.839	46.310
H	31.778	29.602	48.174	H	32.334	30.867	47.473	H	31.952	30.531	46.917	H	32.600	30.662	45.156
C	36.626	28.718	49.211	C	36.512	28.464	49.075	C	36.510	28.586	48.917	C	36.668	28.775	49.252
O	36.117	27.979	50.016	O	35.971	27.802	50.030	O	35.967	27.734	49.672	O	36.125	28.079	50.052
N	31.755	40.719	45.253	N	31.901	40.600	45.957	N	31.486	40.395	45.749	N	31.613	40.650	45.667

H	31.906	40.865	44.234	H	32.047	40.906	44.977	H	31.857	40.969	44.991	H	31.851	40.826	44.655
C	32.783	40.083	46.127	C	32.941	39.821	46.679	C	32.402	39.536	46.503	C	32.509	39.714	46.340
H	32.140	39.342	46.607	H	32.518	39.033	47.245	H	31.843	38.729	46.964	H	31.863	39.010	46.875
C	33.805	39.414	45.149	C	33.947	39.162	45.702	C	33.275	38.768	45.503	C	33.214	38.946	45.217
H	33.275	39.020	44.264	H	33.437	38.754	44.824	H	32.616	38.113	44.963	H	32.510	38.598	44.568
H	34.571	40.086	44.689	H	34.669	39.889	45.432	H	33.673	39.500	44.787	H	33.796	39.688	44.682
C	34.469	38.224	45.868	C	34.668	38.045	46.440	C	34.408	37.960	46.096	C	34.039	37.694	45.667
H	34.968	38.618	46.747	H	34.966	38.426	47.440	H	34.317	37.948	47.167	H	34.779	38.184	46.267
C	33.558	37.108	46.376	C	33.813	36.772	46.578	C	34.224	36.485	45.677	C	33.245	36.574	46.337
H	34.127	36.320	46.729	H	34.466	35.976	46.965	H	33.435	35.981	46.141	H	33.970	35.865	46.673
H	32.891	37.572	47.179	H	33.008	37.038	47.219	H	34.178	36.359	44.570	H	32.686	36.874	47.206
H	32.860	36.803	45.577	H	33.371	36.523	45.594	H	35.073	35.844	45.996	H	32.591	36.081	45.675
C	35.542	37.431	45.063	C	36.030	37.622	45.744	C	35.773	38.366	45.633	C	34.670	37.172	44.399
H	36.051	36.797	45.761	H	36.753	38.496	45.603	H	36.016	39.367	45.851	H	35.327	36.327	44.704
H	35.076	36.839	44.281	H	36.612	36.806	46.306	H	36.467	37.781	46.108	H	33.868	36.819	43.733
H	36.300	38.055	44.598	H	35.842	37.204	44.751	H	35.828	38.203	44.514	H	35.295	37.946	44.001
C	33.452	41.052	47.067	C	33.675	40.801	47.656	C	33.187	40.283	47.598	C	33.487	40.404	47.281
O	33.662	40.703	48.229	O	33.631	40.535	48.866	O	33.327	39.836	48.724	O	33.756	39.898	48.303
N	28.436	43.444	39.050	N	28.495	42.998	40.056	N	28.558	42.996	39.079	N	28.495	42.995	39.995
H	28.351	43.965	38.196	H	28.170	43.297	39.084	H	28.523	43.579	38.281	H	28.407	43.511	39.161
C	29.594	42.622	39.374	C	29.684	42.050	40.126	C	29.785	42.238	39.233	C	29.749	42.243	40.231
H	30.109	43.148	40.184	H	30.316	42.473	40.886	H	30.353	42.744	40.066	H	30.457	42.791	40.900
C	29.253	41.251	39.994	C	29.282	40.557	40.327	C	29.419	40.844	39.638	C	29.494	40.835	40.762
H	29.088	41.391	41.055	H	28.856	40.436	41.329	H	28.940	40.908	40.618	H	29.192	40.928	41.845
H	28.274	40.936	39.575	H	28.551	40.321	39.509	H	28.749	40.419	38.881	H	28.680	40.509	40.151
C	30.304	40.227	39.931	C	30.411	39.582	40.250	C	30.519	39.846	39.653	C	30.610	39.945	40.591
N	31.472	40.293	40.765	N	31.557	39.591	41.081	N	31.839	39.869	40.283	N	31.864	40.125	41.230
H	31.675	41.061	41.390	H	31.835	40.362	41.687	H	32.225	40.747	40.513	H	32.145	41.019	41.656

C	32.276	39.242	40.460	C	32.298	38.429	40.766	C	32.584	38.754	39.789	C	32.687	39.110	40.696
H	33.110	38.994	41.071	H	33.296	37.964	41.196	H	33.613	38.503	39.939	H	33.765	39.098	40.946
N	31.731	38.464	39.484	N	31.597	37.693	39.834	N	31.729	37.990	39.081	N	31.914	38.189	39.986
C	30.476	39.033	39.248	C	30.386	38.374	39.607	C	30.475	38.710	38.947	C	30.657	38.790	39.843
H	29.772	38.481	38.588	H	29.720	37.849	39.013	H	29.812	38.253	38.193	H	30.010	38.361	39.094
C	30.543	42.491	38.140	C	30.487	42.176	38.864	C	30.702	42.248	38.012	C	30.439	42.194	38.875
O	31.642	42.999	38.152	O	31.611	42.629	38.972	O	31.872	42.590	38.119	O	31.637	42.401	38.897
C	32.730	33.047	42.765	C	33.288	32.965	41.784	C	31.838	33.810	41.920	C	33.048	32.879	41.525
C	32.768	33.707	44.145	C	33.448	33.300	43.271	C	32.444	33.003	43.110	C	33.434	33.982	42.445
C	33.779	33.183	45.109	C	34.627	32.639	43.932	C	33.939	33.112	43.257	C	34.319	33.768	43.576
H	33.737	32.953	42.423	H	34.022	32.286	41.479	H	32.676	34.115	41.333	H	33.951	32.715	40.986
H	32.001	33.455	42.094	H	32.425	33.212	41.232	H	31.197	34.517	42.328	H	32.215	33.244	40.991
H	32.621	34.747	44.103	H	33.545	34.341	43.277	H	31.960	33.094	44.078	H	33.030	34.971	42.281
H	34.694	33.653	44.857	H	35.412	32.706	43.234	H	34.362	34.082	43.275	H	35.254	34.074	43.236
H	33.479	33.570	46.051	H	34.840	33.241	44.739	H	34.274	32.645	44.146	H	34.090	34.329	44.435
H	33.770	32.143	45.196	H	34.388	31.650	44.106	H	34.415	32.545	42.521	H	34.375	32.745	43.802
BR	31.063	32.912	44.913	BR	31.884	32.923	44.255	BR	32.169	31.132	42.629	BR	31.298	33.488	43.606
BR	32.260	31.153	43.147	BR	31.824	31.103	42.249	BR	30.864	32.698	40.885	BR	32.658	31.328	42.442

Table S7. Cartesian coordinates (Å) of the LinB first-shell amino acids in complex with the (*R*)-DBP, shown for the representative structure of the largest cluster corresponding to the stationary points of the dehalogenation step. Provided geometries were used as the reference model for SAPT analysis of noncovalent interactions.

**C1**								**C2**							
**RC**				**TS1**				**RC**				**TS1**			
Atom	X	Y	Z	Atom	X	Y	Z	Atom	X	Y	Z	Atom	X	Y	Z
N	35.496	38.963	31.549	N	35.333	39.359	31.584	N	36.184	36.184	31.311	N	36.072	39.185	31.502
H	34.465	38.808	31.425	H	34.341	39.432	31.812	H	35.196	35.196	31.024	H	35.068	39.150	31.374
C	36.236	39.846	30.696	C	35.986	40.377	30.708	C	36.831	36.831	30.661	C	36.709	40.369	30.809

H	37.172	39.280	30.420	H	36.870	39.859	30.439	H	37.786	37.786	30.183	H	37.612	40.103	30.366
C	35.438	40.088	29.406	C	35.085	40.676	29.506	C	35.878	35.878	29.472	C	35.787	40.619	29.588
H	35.514	39.185	28.838	H	35.027	39.807	28.894	H	35.968	35.968	28.796	H	35.706	39.624	29.046
H	34.406	40.220	29.651	H	34.042	40.865	29.720	H	34.879	34.879	29.819	H	34.746	40.882	29.931
C	35.872	41.170	28.426	C	35.779	41.669	28.647	C	36.310	36.310	28.585	C	36.294	41.628	28.608
O	36.916	41.140	27.848	O	36.872	41.430	28.163	O	37.271	37.271	27.899	O	37.480	41.667	28.255
N	35.167	42.234	28.281	N	35.241	42.879	28.546	N	35.545	35.545	28.497	N	35.452	42.509	28.165
H	35.561	42.873	27.640	H	35.770	43.630	28.104	H	35.775	35.775	27.855	H	35.706	43.156	27.336
H	34.302	42.354	28.808	H	34.326	43.114	28.814	H	34.760	34.760	29.134	H	34.493	42.296	28.275
C	36.576	41.204	31.391	C	36.362	41.655	31.500	C	36.970	36.970	31.537	C	36.855	41.610	31.677
O	35.710	41.840	32.027	O	35.515	42.091	32.275	O	36.140	36.140	32.438	O	35.894	41.962	32.287
N	31.124	37.703	36.418	N	31.230	37.757	36.056	N	31.431	31.431	35.723	N	31.271	37.615	36.108
H	30.509	38.209	37.058	H	30.805	38.322	36.802	H	30.722	30.722	36.318	H	30.583	38.005	36.739
C	31.016	38.163	35.004	C	31.009	38.121	34.678	C	31.392	31.392	34.269	C	31.255	38.227	34.715
H	30.087	38.748	34.934	H	30.080	38.594	34.729	H	30.548	30.548	34.111	H	30.340	38.870	34.667
C	32.137	39.176	34.769	C	31.931	39.208	34.199	C	32.623	32.623	33.967	C	32.375	39.164	34.434
H	32.226	39.888	35.605	H	31.769	40.086	34.900	H	32.665	32.665	34.669	H	32.307	39.862	35.227
H	33.137	38.652	34.754	H	32.954	38.863	34.346	H	33.473	33.473	34.128	H	33.328	38.681	34.495
C	32.050	39.876	33.408	C	31.598	39.738	32.819	C	32.670	32.670	32.541	C	32.160	39.816	33.026
O	30.976	40.171	32.904	O	30.572	40.356	32.431	O	31.885	31.885	32.280	O	31.157	40.455	32.685
O	33.137	40.066	32.788	O	32.428	39.457	31.973	O	33.523	33.523	31.690	O	33.024	39.627	32.063
C	30.817	37.046	33.976	C	30.792	36.873	33.796	C	31.144	31.144	33.314	C	31.098	37.049	33.613
O	30.002	36.144	34.120	O	30.120	35.933	34.257	O	30.308	30.308	33.599	O	30.400	36.121	33.904
N	31.562	37.016	32.933	N	31.531	36.783	32.719	N	31.943	31.943	32.242	N	31.890	36.935	32.496
H	32.147	37.830	32.697	H	32.079	37.550	32.440	H	32.572	32.572	31.911	H	32.543	37.686	32.301
C	31.414	35.883	32.012	C	31.481	35.692	31.767	C	31.941	31.941	31.508	C	31.933	35.741	31.651
H	30.414	35.746	31.677	H	30.434	35.516	31.587	H	30.944	30.944	31.128	H	30.969	35.593	31.098
C	32.223	36.134	30.691	C	32.202	36.021	30.509	C	32.652	32.652	30.204	C	33.021	35.963	30.518

H	33.177	36.493	30.878	H	33.021	36.735	30.688	H	33.625	33.625	30.312	H	33.823	36.488	30.894
H	32.408	35.166	30.150	H	32.696	35.103	30.165	H	32.687	32.687	29.784	H	33.307	35.026	30.086
C	31.476	37.014	29.733	C	31.352	36.668	29.433	C	31.948	31.948	29.196	C	32.431	36.759	29.399
C	31.628	38.339	29.475	C	31.308	37.992	29.185	C	32.091	32.091	28.939	C	32.584	38.095	29.089
H	32.386	38.972	29.935	H	31.917	38.733	29.692	H	32.765	32.765	29.387	H	33.309	38.785	29.606
N	30.809	38.635	28.423	N	30.382	38.245	28.163	N	31.321	31.321	27.825	N	31.794	38.461	28.028
H	30.690	39.605	28.107	H	30.125	39.151	27.821	H	31.135	31.135	27.435	H	31.473	39.367	27.763
C	30.066	37.596	28.059	C	29.691	37.059	27.862	C	30.491	30.491	27.522	C	30.999	37.419	27.646
C	29.147	37.475	26.998	C	28.646	36.763	26.960	C	29.543	29.543	26.482	C	30.067	37.189	26.620
H	28.913	38.276	26.345	H	28.227	37.518	26.319	H	29.458	29.458	25.813	H	29.756	38.078	26.064
C	28.349	36.288	26.966	C	28.181	35.436	26.913	C	28.870	28.870	26.290	C	29.455	35.969	26.463
H	27.523	36.166	26.244	H	27.380	35.139	26.277	H	28.071	28.071	25.598	H	28.907	35.757	25.506
C	28.679	35.200	27.847	C	28.743	34.414	27.723	C	29.212	29.212	27.113	C	29.765	34.938	27.326
H	28.136	34.247	27.695	H	28.283	33.470	27.789	H	28.705	28.705	26.968	H	29.205	34.019	27.168
C	29.654	35.379	28.837	C	29.793	34.772	28.589	C	30.192	30.192	28.113	C	30.753	35.077	28.277
H	29.877	34.532	29.488	H	30.308	34.015	29.170	H	30.518	30.518	28.683	H	31.069	34.206	28.756
C	30.369	36.505	28.901	C	30.326	36.044	28.624	C	30.832	30.832	28.371	C	31.403	36.317	28.476
C	31.764	34.555	32.654	C	31.972	34.393	32.417	C	32.384	32.384	32.167	C	32.196	34.399	32.378
O	31.197	33.583	32.219	O	31.450	33.321	32.066	O	32.023	32.023	31.779	O	31.732	33.298	32.042
N	28.500	39.652	40.970	N	27.854	38.975	41.112	N	27.534	27.534	40.369	N	27.564	39.463	40.392
H	29.376	39.897	41.422	H	28.448	38.129	41.047	H	28.054	28.054	40.917	H	28.390	39.756	40.922
C	28.100	40.233	39.661	C	27.359	39.647	39.883	C	27.479	27.479	38.977	C	27.398	40.099	39.038
H	27.086	40.555	39.787	H	26.383	40.059	39.979	H	26.492	26.492	38.847	H	26.411	40.512	38.967
C	28.950	41.404	39.292	C	28.379	40.745	39.395	C	28.547	28.547	38.844	C	28.259	41.424	38.908
H	29.986	41.140	39.187	H	29.383	40.413	39.307	H	29.482	29.482	39.206	H	29.325	41.272	39.172
H	28.595	41.769	38.326	H	28.100	40.946	38.355	H	28.570	28.570	37.772	H	28.106	41.694	37.895
C	28.921	42.623	40.271	C	28.226	42.201	40.041	C	28.216	28.216	39.787	C	27.753	42.501	39.832
H	29.158	42.209	41.270	H	28.048	42.074	41.102	H	28.674	28.674	40.750	H	28.071	42.282	40.857

H	29.727	43.317	39.919	H	29.170	42.671	39.980	H	28.886	28.886	39.553	H	28.306	43.384	39.572
C	27.546	43.323	40.129	C	27.116	42.995	39.399	C	26.747	26.747	39.679	C	26.251	42.644	39.909
O	27.340	44.179	39.187	O	27.321	43.947	38.565	O	26.295	26.295	38.730	O	25.627	43.447	39.103
O	26.705	43.205	41.073	O	25.897	42.816	39.739	O	25.889	25.889	40.522	O	25.553	42.074	40.780
C	28.221	39.217	38.516	C	27.391	38.467	38.856	C	27.709	27.709	38.022	C	27.774	39.069	37.960
O	29.193	38.414	38.490	O	28.401	37.778	38.678	O	28.857	28.857	37.849	O	28.855	38.506	37.904
N	25.877	37.260	34.785	N	25.885	36.729	34.847	N	26.009	26.009	33.977	N	26.197	36.818	34.102
H	26.501	36.478	34.854	H	26.777	36.310	34.835	H	26.819	26.819	33.923	H	26.881	36.101	34.257
C	24.914	37.284	33.676	C	25.004	36.383	33.764	C	25.009	25.009	32.907	C	25.303	36.548	32.959
H	24.504	38.285	33.552	H	24.366	37.187	33.539	H	24.447	24.447	32.608	H	24.862	37.476	32.637
C	25.739	36.972	32.415	C	25.845	36.072	32.482	C	25.594	25.594	31.562	C	26.097	36.083	31.676
H	26.335	36.039	32.491	H	26.281	35.101	32.610	H	26.053	26.053	31.725	H	26.557	35.107	31.788
C	24.749	36.714	31.250	C	24.879	35.851	31.264	C	24.545	24.545	30.421	C	25.172	35.992	30.472
H	25.150	36.088	30.514	H	25.448	35.123	30.612	H	23.635	23.635	30.867	H	24.771	36.993	30.146
H	23.776	36.267	31.501	H	23.954	35.390	31.456	H	24.162	24.162	30.002	H	25.759	35.699	29.573
H	24.505	37.638	30.773	H	24.877	36.784	30.706	H	24.897	24.897	29.665	H	24.484	35.193	30.625
C	26.829	38.023	32.030	C	27.042	37.057	32.229	C	26.655	26.655	31.113	C	27.231	37.125	31.302
H	26.299	38.873	31.578	H	26.621	38.079	32.108	H	26.222	26.222	30.915	H	26.705	38.088	31.158
H	27.374	38.363	32.890	H	27.596	37.040	33.099	H	27.399	27.399	31.847	H	27.928	37.320	32.090
C	27.802	37.572	30.983	C	27.914	36.817	31.021	C	27.420	27.420	29.835	C	28.067	36.923	30.088
H	28.432	36.809	31.372	H	28.471	35.890	31.148	H	27.778	27.778	30.043	H	28.412	35.886	29.953
H	27.275	37.184	30.136	H	27.378	36.849	30.068	H	26.775	26.775	29.053	H	27.500	37.015	29.184
H	28.386	38.450	30.650	H	28.746	37.526	30.936	H	28.150	28.150	29.589	H	28.870	37.628	30.074
C	23.766	36.305	34.017	C	24.027	35.290	34.147	C	24.039	24.039	33.477	C	24.125	35.613	33.385
O	23.601	35.242	33.397	O	24.217	34.078	33.835	O	24.109	24.109	33.122	O	24.041	34.479	32.895
N	20.941	48.315	30.538	N	19.639	47.652	30.268	N	20.406	20.406	29.771	N	20.769	47.515	29.485
H	20.406	48.137	29.705	H	19.401	47.325	29.269	H	20.221	20.221	28.777	H	20.301	47.293	28.637
C	22.151	49.178	30.491	C	20.791	48.485	30.493	C	21.608	21.608	29.989	C	21.787	48.639	29.568

H	22.626	48.855	31.467	H	21.227	48.250	31.485	H	22.085	22.085	30.928	H	22.419	48.303	30.384
C	22.997	48.719	29.342	C	21.881	48.267	29.444	C	22.589	22.589	28.872	C	22.606	48.602	28.292
H	22.395	48.804	28.450	H	21.620	48.506	28.429	H	22.020	22.020	27.956	H	21.958	48.758	27.360
H	23.828	49.398	29.271	H	22.763	48.890	29.677	H	23.440	23.440	28.897	H	23.397	49.357	28.380
C	23.393	47.254	29.575	C	22.509	46.867	29.500	C	23.117	23.117	28.852	C	23.321	47.215	28.026
C	23.188	46.397	28.510	C	22.742	46.134	28.298	C	23.096	23.096	27.608	C	24.647	47.077	28.396
H	22.622	46.681	27.653	H	22.556	46.501	27.324	H	22.598	22.598	26.764	H	25.245	47.942	28.737
C	23.709	45.084	28.671	C	23.384	44.891	28.327	C	23.737	23.737	27.501	C	25.286	45.849	28.231
H	23.589	44.395	27.799	H	23.596	44.308	27.383	H	23.916	23.916	26.554	H	26.311	45.671	28.582
C	24.526	44.714	29.758	C	23.890	44.400	29.542	C	24.351	24.351	28.641	C	24.675	44.780	27.583
H	24.908	43.717	29.759	H	24.472	43.462	29.576	H	24.876	24.876	28.607	H	25.279	43.937	27.363
C	24.591	45.576	30.830	C	23.771	45.181	30.726	C	24.303	24.303	29.901	C	23.329	44.864	27.151
H	25.131	45.282	31.674	H	24.271	44.825	31.601	H	24.721	24.721	30.737	H	22.850	44.075	26.570
C	24.040	46.852	30.717	C	23.083	46.414	30.717	C	23.632	23.632	30.011	C	22.677	46.112	27.491
H	24.235	47.572	31.540	H	22.996	47.011	31.578	H	23.526	23.526	30.929	H	21.656	46.199	27.142
C	21.803	50.692	30.457	C	20.400	50.020	30.448	C	21.374	21.374	29.958	C	21.075	50.016	29.747
O	21.050	51.083	29.544	O	19.590	50.390	29.590	O	20.490	20.490	29.217	O	20.164	50.341	29.045
N	28.788	50.221	24.871	N	27.915	49.797	25.100	N	28.782	28.782	24.829	N	28.780	50.383	24.795
H	28.171	50.585	25.642	H	27.292	49.961	25.868	H	28.074	28.074	25.523	H	28.239	50.609	25.561
C	28.637	48.872	24.414	C	27.713	48.474	24.395	C	28.806	28.806	24.161	C	28.838	49.020	24.248
H	29.666	48.423	24.516	H	28.594	47.884	24.284	H	29.755	29.755	24.322	H	29.855	48.732	24.146
C	27.605	48.070	25.181	C	26.896	47.504	25.251	C	27.704	27.704	24.765	C	28.186	48.001	25.234
H	26.741	48.731	25.282	H	25.848	47.751	25.348	H	26.814	26.814	24.630	H	27.202	48.263	25.561
H	27.197	47.176	24.624	H	26.791	46.563	24.676	H	27.690	27.690	24.170	H	28.136	46.978	24.723
C	27.961	47.781	26.595	C	27.464	47.139	26.645	C	27.951	27.951	26.152	C	28.954	47.820	26.478
C	26.984	48.030	27.580	C	26.656	47.380	27.802	C	26.912	26.912	27.086	C	29.820	46.731	26.597
H	26.048	48.494	27.249	H	25.694	47.746	27.657	H	26.072	26.072	26.744	H	29.844	45.970	25.867
C	27.234	47.758	28.951	C	27.148	47.283	29.124	C	27.096	27.096	28.426	C	30.494	46.548	27.764

H	26.394	47.891	29.720	H	26.531	47.735	29.897	H	26.375	26.375	29.194	H	31.012	45.614	27.939
C	28.539	47.308	29.310	C	28.504	46.920	29.284	C	28.211	28.211	28.802	C	30.479	47.471	28.850
H	28.860	47.166	30.363	H	28.944	46.853	30.288	H	28.446	28.446	29.833	H	31.009	47.365	29.714
C	29.522	47.033	28.340	C	29.241	46.580	28.153	C	29.229	29.229	27.793	C	29.585	48.530	28.730
H	30.545	46.723	28.581	H	30.257	46.193	28.338	H	30.099	30.099	28.166	H	29.631	49.350	29.415
C	29.198	47.228	26.985	C	28.819	46.755	26.823	C	29.152	29.152	26.489	C	28.831	48.709	27.565
H	29.859	46.991	26.154	H	29.474	46.560	25.989	H	30.027	30.027	25.842	H	28.115	49.559	27.576
C	28.096	48.818	22.932	C	27.256	48.586	22.898	C	28.563	28.563	22.687	C	28.235	48.955	22.871
O	28.698	48.194	22.072	O	27.622	47.678	22.188	O	29.267	29.267	21.927	O	28.650	48.039	22.117
N	38.367	48.970	26.993	N	38.045	49.054	27.108	N	39.240	39.240	27.152	N	38.879	49.389	27.346
H	38.570	48.585	26.090	H	38.380	48.547	26.279	H	39.756	39.756	26.335	H	39.100	49.132	26.454
C	37.719	48.112	27.985	C	37.405	48.201	28.192	C	38.699	38.699	28.135	C	38.300	48.459	28.363
H	36.810	48.608	28.355	H	36.516	48.736	28.542	H	37.771	37.771	28.514	H	37.371	48.884	28.694
C	37.366	46.763	27.307	C	36.940	46.843	27.604	C	38.273	38.273	27.527	C	37.854	47.108	27.663
H	37.067	46.967	26.248	H	36.713	47.183	26.612	H	38.367	38.367	26.470	H	37.689	47.256	26.550
H	38.282	46.145	27.225	H	37.670	46.111	27.546	H	38.875	38.875	27.849	H	38.617	46.367	27.824
C	36.158	46.053	27.945	C	35.608	46.384	28.120	C	36.786	36.786	27.779	C	36.531	46.547	28.192
C	35.012	45.878	27.172	C	34.591	46.058	27.214	C	35.843	35.843	26.775	C	35.324	46.639	27.418
H	35.014	46.192	26.150	H	34.888	46.015	26.123	H	36.106	36.106	25.707	H	35.246	47.176	26.482
C	33.823	45.252	27.738	C	33.264	45.669	27.607	C	34.498	34.498	27.149	C	34.107	46.066	27.811
H	32.916	45.078	27.185	H	32.533	45.372	26.924	H	33.725	33.725	26.345	H	33.241	46.190	27.106
C	33.835	44.851	29.092	C	33.074	45.491	28.967	C	34.039	34.039	28.449	C	34.030	45.414	29.066
H	32.914	44.352	29.551	H	32.214	45.047	29.370	H	33.028	33.028	28.747	H	33.034	45.053	29.414
C	34.956	45.065	29.811	C	34.112	45.778	29.936	C	34.989	34.989	29.387	C	35.128	45.332	29.919
H	34.996	44.665	30.845	H	33.873	45.719	30.963	H	34.576	34.576	30.417	H	35.035	44.766	30.797
C	36.163	45.611	29.254	C	35.368	46.209	29.487	C	36.315	36.315	29.083	C	36.425	45.726	29.420
H	37.011	45.770	29.960	H	36.164	46.399	30.173	H	36.910	36.910	29.919	H	37.290	45.513	30.064
C	38.724	47.875	29.075	C	38.409	48.036	29.387	C	39.560	39.560	29.346	C	39.102	48.282	29.710

O	38.358	47.978	30.233	O	38.004	48.211	30.547	O	39.086	39.086	30.475	O	38.642	48.080	30.787
N	37.433	51.480	31.176	N	36.767	51.381	31.422	N	37.719	37.719	31.258	N	36.956	51.411	31.502
H	37.908	51.188	30.305	H	37.095	50.967	30.594	H	38.230	38.230	30.500	H	37.567	51.112	30.729
C	36.314	50.736	31.817	C	35.631	50.797	32.138	C	36.435	36.435	31.697	C	35.784	50.638	31.916
H	35.673	51.412	32.376	H	35.045	51.627	32.444	H	35.674	35.674	32.040	H	35.000	51.320	32.181
C	35.490	49.781	30.887	C	34.822	49.910	31.191	C	35.745	35.745	30.571	C	35.256	49.726	30.757
H	36.142	49.045	30.417	H	35.524	49.166	30.835	H	36.599	36.599	30.147	H	36.012	48.966	30.478
C	34.334	49.055	31.605	C	33.691	49.162	31.903	C	34.527	34.527	31.010	C	33.910	49.046	31.066
H	33.690	49.803	32.052	H	32.845	48.892	31.256	H	34.133	34.133	30.164	H	33.725	48.500	30.124
H	33.909	48.334	30.980	H	33.941	48.246	32.349	H	34.768	34.768	31.836	H	34.036	48.275	31.842
H	34.794	48.505	32.431	H	33.309	49.831	32.663	H	33.589	33.589	31.298	H	33.101	49.694	31.405
C	34.794	50.550	29.717	C	34.231	50.540	29.990	C	35.187	35.187	29.493	C	34.996	50.608	29.471
H	35.625	51.002	29.169	H	33.322	51.089	30.409	H	34.405	34.405	30.043	H	34.210	51.316	29.807
H	34.384	49.815	29.030	H	34.887	51.237	29.592	H	36.033	36.033	29.270	H	36.010	50.958	29.213
H	33.942	51.258	29.915	H	33.869	49.759	29.267	H	34.743	34.743	28.665	H	34.686	50.002	28.598
C	36.790	49.920	33.051	C	36.105	50.075	33.438	C	36.719	36.719	32.947	C	36.059	49.809	33.203
O	36.133	50.122	34.065	O	35.515	50.304	34.453	O	36.016	36.016	33.922	O	35.204	49.710	34.041
N	33.503	39.445	23.382	N	33.744	39.575	23.743	N	34.395	34.395	23.199	N	34.618	39.657	23.621
H	34.403	39.879	23.561	H	34.725	39.794	23.948	H	35.397	35.397	23.409	H	35.650	39.800	23.735
C	32.553	39.396	24.493	C	32.919	39.311	24.902	C	33.437	33.437	24.325	C	33.776	39.444	24.851
H	32.101	38.405	24.364	H	32.533	38.265	24.742	H	32.968	32.968	24.352	H	33.317	38.464	24.927
C	33.331	39.293	25.797	C	33.833	39.295	26.122	C	34.313	34.313	25.663	C	34.598	39.528	26.152
H	33.993	40.147	25.880	H	34.344	40.183	26.124	H	35.050	35.050	25.599	H	35.246	40.422	26.156
H	32.654	39.516	26.618	H	33.200	39.266	27.012	H	33.553	33.553	26.470	H	33.847	39.635	26.938
C	34.149	38.075	26.128	C	34.672	38.113	26.375	C	35.002	35.002	26.185	C	35.392	38.230	26.507
C	35.474	38.031	26.466	C	35.995	38.107	26.697	C	36.321	36.321	26.491	C	36.687	38.250	26.844
H	36.241	38.836	26.453	H	36.677	38.982	26.688	H	37.103	37.103	26.415	H	37.221	39.139	27.029
N	35.763	36.761	26.932	N	36.410	36.810	26.958	N	36.616	36.616	26.777	N	37.140	36.934	26.965

H	36.569	36.514	27.394	H	37.414	36.561	27.106	H	37.519	37.519	27.088	H	38.068	36.692	27.117
C	34.713	35.903	26.788	C	35.406	35.906	26.999	C	35.553	35.553	26.855	C	36.128	36.042	26.890
C	34.484	34.533	27.126	C	35.264	34.564	27.396	C	35.326	35.326	27.198	C	36.062	34.612	27.062
H	35.223	34.000	27.676	H	36.173	34.045	27.704	H	36.199	36.199	27.404	H	36.864	33.930	27.456
C	33.272	33.992	26.684	C	34.023	33.870	27.343	C	34.068	34.068	27.016	C	34.807	34.051	26.940
H	33.065	32.971	26.852	H	33.993	32.840	27.588	H	33.918	33.918	27.277	H	34.719	33.023	27.161
C	32.224	34.803	26.244	C	32.952	34.606	26.771	C	33.004	33.004	26.465	C	33.688	34.809	26.556
H	31.279	34.396	25.962	H	31.964	34.109	26.719	H	32.002	32.002	26.346	H	32.718	34.371	26.460
C	32.454	36.158	26.018	C	33.116	35.939	26.338	C	33.205	33.205	26.207	C	33.792	36.118	26.309
H	31.677	36.753	25.562	H	32.203	36.436	26.005	H	32.336	32.336	25.964	H	32.934	36.637	26.028
C	33.676	36.700	26.224	C	34.305	36.650	26.531	C	34.500	34.500	26.363	C	34.986	36.792	26.559
C	31.392	40.433	24.505	C	31.674	40.206	24.978	C	32.268	32.268	24.353	C	32.465	40.288	24.771
O	30.210	40.076	24.509	O	30.580	39.658	25.076	O	31.149	31.149	24.337	O	31.378	39.691	24.865
N	31.709	41.731	24.258	N	31.729	41.625	24.873	N	32.334	32.334	24.333	N	28.275	38.660	23.665
C	33.048	42.401	24.394	C	32.899	42.480	25.053	C	33.578	33.578	24.172	H	28.797	39.522	23.548
H	33.820	41.880	23.759	H	33.749	41.948	24.596	H	34.205	34.205	23.442	C	27.339	38.623	24.840
H	33.537	42.394	25.419	H	33.079	42.654	26.093	H	34.127	34.127	25.098	H	27.761	37.885	25.453
C	32.836	43.840	23.892	C	32.533	43.813	24.321	C	33.203	33.203	23.629	C	27.117	40.005	25.521
H	33.081	43.842	22.827	H	32.560	43.661	23.239	H	33.046	33.046	22.531	H	26.764	40.744	24.742
H	33.394	44.603	24.388	H	33.217	44.655	24.641	H	34.065	34.065	23.910	C	26.050	39.875	26.630
C	31.347	44.109	24.032	C	31.082	43.949	24.711	C	31.928	31.928	24.437	H	26.493	39.313	27.474
H	31.037	44.796	23.266	H	30.542	44.603	23.970	H	31.337	31.337	24.036	H	25.675	40.831	26.964
H	31.181	44.525	25.002	H	31.045	44.349	25.768	H	32.262	32.262	25.534	H	25.201	39.363	26.234
C	30.653	42.732	24.027	C	30.537	42.507	24.692	C	31.220	31.220	24.235	C	28.495	40.647	26.033
H	30.125	42.600	25.012	H	29.811	42.428	25.553	H	30.528	30.528	25.048	H	28.941	40.037	26.738
C	29.670	42.485	22.837	C	29.821	42.123	23.407	C	30.326	30.326	22.976	H	29.153	40.772	25.156
O	28.567	43.075	22.887	O	28.724	42.686	23.222	O	29.142	29.142	22.955	C	28.154	42.005	26.620
N	26.933	38.908	23.972	N	27.214	38.660	23.947	N	27.623	27.623	23.648	H	27.303	42.419	26.052

H	27.531	39.684	23.791	H	27.845	39.507	23.888	H	28.169	28.169	23.436	H	27.907	41.952	27.680
C	25.884	39.059	24.991	C	26.044	38.680	24.940	C	26.627	26.627	24.810	H	29.052	42.656	26.594
H	26.234	38.521	25.914	H	26.371	38.209	25.805	H	27.025	27.025	25.595	C	25.987	38.025	24.318
C	25.699	40.530	25.475	C	25.861	40.225	25.303	C	26.393	26.393	25.196	O	25.529	38.572	23.327
H	25.198	41.117	24.710	H	25.584	40.633	24.436	H	25.962	25.962	24.424	N	25.906	44.621	36.122
C	24.825	40.530	26.734	C	24.788	40.464	26.286	C	25.363	25.363	26.366	H	26.793	44.669	36.673
H	25.260	39.903	27.462	H	23.884	39.942	26.047	H	24.394	24.394	26.014	C	25.600	43.700	35.030
H	24.625	41.554	27.171	H	25.125	40.091	27.239	H	25.778	25.778	27.153	H	25.353	44.257	34.059
H	23.834	40.005	26.547	H	24.497	41.526	26.458	H	25.262	25.262	26.496	C	26.776	42.707	34.869
C	27.093	41.228	25.744	C	27.104	41.022	25.700	C	27.702	27.702	25.669	H	27.582	43.300	34.508
H	27.580	40.908	26.616	H	27.458	40.568	26.617	H	28.102	28.102	26.551	H	27.064	42.352	35.853
H	27.829	41.113	24.958	H	27.810	40.911	24.901	H	28.430	28.430	24.896	C	26.504	41.652	33.832
C	26.901	42.781	25.901	C	26.885	42.443	25.931	C	27.702	27.702	25.996	H	25.811	40.979	34.244
H	26.142	43.182	25.232	H	26.351	42.666	26.867	H	27.410	27.410	25.221	C	26.220	42.187	32.415
H	26.689	43.087	26.936	H	27.778	42.992	26.147	H	27.205	27.205	26.955	H	25.209	42.676	32.281
H	27.763	43.363	25.557	H	26.371	42.853	25.121	H	28.702	28.702	26.097	H	26.995	42.869	32.112
C	24.507	38.501	24.513	C	24.676	38.121	24.455	C	25.301	25.301	24.363	H	26.099	41.346	31.660
O	24.218	38.814	23.376	O	24.149	38.611	23.480	O	24.832	24.832	23.284	C	27.764	40.694	33.771
N	26.648	45.350	36.665	N	26.166	44.951	36.103	N	25.892	25.892	36.144	H	27.878	40.256	34.740
H	27.338	45.121	37.386	H	27.006	44.783	36.661	H	26.430	26.430	37.001	H	27.652	39.904	32.948
C	26.295	44.216	35.833	C	25.853	43.898	35.143	C	25.748	25.748	35.284	H	28.611	41.303	33.579
H	26.234	44.629	34.802	H	25.744	44.410	34.163	H	25.549	25.549	34.224	C	24.260	42.837	35.226
C	27.475	43.197	35.796	C	27.001	42.880	35.034	C	26.958	26.958	35.164	O	23.418	42.696	34.345
H	28.377	43.776	35.454	H	27.898	43.191	34.468	H	27.758	27.758	34.654	N	30.858	46.923	40.387
H	27.724	42.818	36.809	H	27.352	42.664	36.088	H	27.219	27.219	36.143	H	31.678	47.139	40.967
C	27.267	41.994	34.876	C	26.619	41.464	34.606	C	26.638	26.638	34.300	C	30.732	45.660	39.718
H	26.303	41.552	34.999	H	25.863	41.032	35.208	H	26.017	26.017	34.783	H	29.758	45.228	39.911
C	27.431	42.418	33.428	C	26.184	41.303	33.173	C	26.087	26.087	32.907	C	30.754	45.853	38.162

H	27.494	41.468	32.806	H	26.704	41.988	32.521	H	26.349	26.349	32.270	H	29.906	46.511	37.939
H	26.686	43.156	33.201	H	26.305	40.285	32.833	H	25.008	25.008	32.978	H	31.630	46.466	37.926
H	28.404	42.906	33.296	H	25.135	41.489	33.032	H	26.492	26.492	32.419	C	30.584	44.636	37.309
C	28.298	40.937	35.115	C	27.926	40.594	34.673	C	27.953	27.953	33.971	N	31.251	43.454	37.485
H	27.902	40.103	34.628	H	28.791	41.082	34.172	H	27.645	27.645	33.600	H	31.777	43.299	38.331
H	29.305	41.198	34.768	H	28.210	40.529	35.705	H	28.515	28.515	33.223	C	30.832	42.573	36.523
H	28.292	40.628	36.168	H	27.782	39.541	34.409	H	28.564	28.564	34.833	H	31.175	41.497	36.483
C	24.932	43.565	36.142	C	24.495	43.169	35.559	C	24.473	24.473	35.665	N	30.004	43.207	35.645
O	24.063	43.673	35.297	O	23.623	42.960	34.734	O	23.470	23.470	34.895	C	29.813	44.456	36.131
N	32.112	47.415	40.176	N	31.068	47.262	40.445	N	31.141	31.141	39.519	H	29.337	45.292	35.606
H	32.933	47.554	40.754	H	31.790	47.316	41.021	H	31.990	31.990	39.935	C	31.757	44.612	40.160
C	31.869	46.046	39.686	C	30.782	45.976	39.808	C	31.150	31.150	38.887	O	31.329	43.514	40.585
H	30.951	45.734	40.250	H	29.880	45.454	40.251	H	30.182	30.182	39.016	C	31.060	42.576	31.079
C	31.736	46.079	38.177	C	30.515	46.210	38.352	C	31.261	31.261	37.436	BR	30.062	43.289	29.616
H	30.708	46.348	37.910	H	29.518	46.658	38.294	H	30.490	30.490	37.109	C	31.362	41.206	30.720
H	32.422	46.764	37.639	H	31.176	47.044	38.043	H	32.230	32.230	37.239	BR	32.139	41.907	28.536
C	31.854	44.845	37.369	C	30.736	44.981	37.562	C	31.279	31.279	36.691	H	32.446	40.804	30.880
N	31.289	43.583	37.588	N	29.892	43.813	37.461	N	30.746	30.746	36.986	H	30.570	42.649	32.005
H	30.625	43.516	38.285	H	29.061	43.754	38.040	H	30.381	30.381	37.847	H	31.946	43.220	31.106
C	31.559	42.765	36.539	C	30.575	42.863	36.726	C	31.098	31.098	35.943	C	30.207	40.448	30.126
H	31.237	41.819	36.322	H	30.213	41.858	36.554	H	30.740	30.740	35.917	H	30.395	39.429	30.376
N	32.480	43.383	35.751	N	31.676	43.415	36.259	N	31.766	31.766	34.969	H	30.192	40.441	29.098
C	32.722	44.677	36.250	C	31.808	44.704	36.805	C	31.929	31.929	35.439	H	29.309	40.847	30.515
H	33.401	45.329	35.776	H	32.574	45.327	36.588	H	32.561	32.561	34.985	N	36.072	39.185	31.502
C	32.967	45.023	40.042	C	31.974	45.043	40.042	C	32.238	32.238	39.520	H	35.068	39.150	31.374
O	32.593	43.975	40.497	O	31.746	43.875	40.385	O	31.890	31.890	40.059	C	36.709	40.369	30.809
C	29.478	41.370	30.355	C	29.246	40.673	30.809	C	29.373	29.373	29.826	H	37.612	40.103	30.366
BR	27.789	40.940	29.425	BR	27.279	40.520	29.703	BR	27.453	27.453	29.369	C	35.787	40.619	29.588

C	29.930	42.669	29.641	C	29.712	41.911	30.075	C	30.221	30.221	30.053	H	35.706	39.624	29.046
BR	28.336	43.527	29.380	BR	28.374	43.076	29.757	BR	28.961	28.961	30.454	H	34.746	40.882	29.931
H	30.607	43.242	30.189	H	30.370	42.348	30.761	H	30.752	30.752	31.021	C	36.294	41.628	28.608
H	29.943	40.460	30.288	H	29.579	39.684	30.661	H	29.627	29.627	28.945	O	37.480	41.667	28.255
H	29.219	41.558	31.356	H	28.909	40.832	31.819	H	29.183	29.183	30.620	N	35.452	42.509	28.165
C	30.489	42.380	28.274	C	30.360	41.739	28.698	C	30.965	30.965	28.797	H	35.706	43.156	27.336
H	29.723	41.832	27.655	H	29.692	41.305	27.966	H	31.322	31.322	28.925	H	34.493	42.296	28.275
H	31.353	41.693	28.421	H	31.316	41.243	28.949	H	30.358	30.358	27.949	C	36.855	41.610	31.677
H	30.839	43.267	27.689	H	30.720	42.734	28.333	H	31.714	31.714	28.632	O	35.894	41.962	32.287

Table S8. Cartesian coordinates (Å) of the LinB first-shell amino acids in complex with the (*S*)-DCP, shown for the representative structure of the largest cluster corresponding to the stationary points of the dehalogenation step. Provided geometries were used as the reference model for SAPT analysis of noncovalent interactions.

**C1**								**C2**							
**RC**				**TS1**				**RC**				**TS1**			
Atom	X	Y	Z	Atom	X	Y	Z	Atom	X	Y	Z	Atom	X	Y	Z
N	37.992	40.800	34.318	N	37.816	40.587	34.412	N	37.972	40.969	34.588	N	37.942	41.428	34.353
H	38.335	39.859	34.226	H	38.184	39.707	34.534	H	38.401	40.060	34.504	H	38.464	40.603	34.052
C	37.364	41.387	33.089	C	37.182	40.990	33.141	C	37.331	41.447	33.375	C	37.121	42.120	33.275
H	37.714	42.390	32.936	H	37.226	42.023	33.151	H	37.213	42.526	33.498	H	36.957	43.086	33.641
C	37.979	40.571	31.966	C	38.146	40.597	31.965	C	38.179	41.085	32.123	C	38.017	42.209	32.052
H	39.066	40.703	32.053	H	39.074	41.154	32.028	H	39.059	41.703	32.113	H	38.786	42.982	32.275
H	37.814	39.543	32.110	H	38.407	39.564	32.075	H	38.499	40.072	32.219	H	38.512	41.270	31.968
C	37.511	41.113	30.651	C	37.614	40.829	30.604	C	37.538	41.287	30.815	C	37.307	42.652	30.765
O	37.953	42.183	30.322	O	37.248	41.917	30.230	O	37.639	42.326	30.185	O	37.202	43.867	30.529
N	36.483	40.624	29.974	N	37.505	39.790	29.811	N	36.893	40.290	30.250	N	36.947	41.697	29.926
H	36.184	41.126	29.154	H	37.183	39.985	28.885	H	36.643	40.404	29.291	H	36.293	41.991	29.268
H	35.916	39.864	30.255	H	37.896	38.887	30.137	H	36.562	39.467	30.709	H	36.995	40.690	30.145

C	35.824	41.293	33.007	C	35.738	40.463	32.938	C	35.907	40.961	33.161	C	35.812	41.334	33.010
O	35.309	40.311	33.486	O	35.624	39.232	32.987	O	35.776	39.769	33.121	O	35.766	40.097	33.044
N	38.567	35.279	38.693	N	38.804	35.377	38.836	N	38.501	35.576	38.419	N	39.180	35.580	38.336
H	37.997	34.461	38.754	H	38.469	34.452	38.926	H	38.035	34.659	38.383	H	38.947	34.624	38.430
C	39.012	35.702	37.339	C	39.153	35.741	37.477	C	38.974	36.017	37.067	C	39.439	36.083	36.978
H	38.982	34.829	36.696	H	39.220	34.895	36.888	H	39.119	35.202	36.478	H	39.421	35.153	36.381
C	37.937	36.680	36.788	C	37.992	36.545	36.834	C	37.814	36.850	36.521	C	38.287	36.940	36.485
H	37.057	36.266	37.090	H	37.034	36.092	36.949	H	36.933	36.229	36.776	H	37.365	36.440	36.654
H	38.147	37.567	37.307	H	37.969	37.428	37.440	H	37.648	37.723	37.212	H	38.264	37.857	37.002
C	37.958	36.941	35.242	C	38.235	36.832	35.382	C	37.964	37.208	35.008	C	38.454	37.271	34.961
O	37.497	36.137	34.360	O	37.992	35.855	34.508	O	37.487	36.443	34.150	O	38.601	36.190	34.217
O	38.535	38.056	34.973	O	38.528	37.951	34.980	O	38.478	38.271	34.658	O	38.572	38.440	34.573
C	40.473	36.220	37.310	C	40.628	36.342	37.365	C	40.407	36.616	37.162	C	40.847	36.739	37.056
O	41.296	35.556	37.991	O	41.555	35.721	37.947	O	41.304	36.118	37.932	O	41.726	36.049	37.581
N	40.823	37.277	36.565	N	40.859	37.446	36.632	N	40.716	37.734	36.449	N	41.138	37.925	36.483
H	40.090	37.515	35.918	H	40.172	37.900	36.012	H	40.034	38.068	35.857	H	40.506	38.339	35.841
C	42.039	38.114	36.582	C	42.202	38.124	36.552	C	42.060	38.363	36.491	C	42.384	38.609	36.493
H	42.906	37.462	36.306	H	42.924	37.285	36.309	H	42.733	37.596	36.260	H	43.079	37.823	36.134
C	41.957	39.195	35.485	C	42.260	39.110	35.431	C	42.239	39.471	35.477	C	42.504	39.739	35.448
H	41.068	39.824	35.542	H	41.379	39.744	35.412	H	41.340	40.042	35.322	H	41.567	40.357	35.629
H	42.823	39.874	35.613	H	43.117	39.733	35.602	H	42.947	40.270	35.752	H	43.453	40.352	35.564
C	42.204	38.772	34.077	C	42.366	38.494	34.114	C	42.607	38.890	34.151	C	42.476	39.131	34.103
C	41.332	38.723	33.021	C	41.342	38.261	33.256	C	41.655	38.732	33.164	C	41.553	39.236	33.186
H	40.337	39.069	33.084	H	40.331	38.525	33.441	H	40.601	39.027	33.305	H	40.621	39.857	33.258
N	41.988	38.082	31.961	N	41.773	37.599	32.174	N	42.160	38.093	32.098	N	41.944	38.611	32.044
H	41.539	37.966	31.050	H	41.099	37.351	31.459	H	41.590	37.743	31.423	H	41.415	38.512	31.185
C	43.266	37.736	32.272	C	43.156	37.329	32.260	C	43.472	37.803	32.307	C	43.215	38.147	32.199
C	44.242	37.061	31.586	C	44.092	36.686	31.454	C	44.417	37.165	31.488	C	44.144	37.595	31.271

H	44.149	36.921	30.531	H	43.772	36.307	30.507	H	44.085	36.789	30.516	H	43.876	37.485	30.272
C	45.444	36.839	32.324	C	45.449	36.688	31.823	C	45.655	36.802	32.069	C	45.496	37.461	31.676
H	46.197	36.394	31.693	H	46.159	36.292	31.185	H	46.381	36.125	31.613	H	46.333	37.099	31.031
C	45.695	37.134	33.616	C	45.892	37.169	33.073	C	45.988	37.190	33.396	C	45.862	37.667	32.990
H	46.676	36.926	34.109	H	46.892	37.079	33.349	H	46.870	36.898	33.896	H	46.913	37.640	33.267
C	44.742	37.879	34.303	C	44.959	37.791	33.917	C	45.060	37.984	34.143	C	44.899	38.136	33.944
H	44.939	38.150	35.296	H	45.215	38.143	34.920	H	45.222	38.297	35.103	H	45.257	38.424	34.918
C	43.415	38.152	33.661	C	43.556	37.869	33.502	C	43.805	38.297	33.620	C	43.552	38.445	33.527
C	42.501	38.637	37.983	C	42.730	38.717	37.896	C	42.503	38.806	37.896	C	42.870	38.873	37.855
O	43.707	38.684	38.323	O	43.941	38.807	38.147	O	43.691	38.835	38.168	O	44.040	38.545	38.164
N	36.322	30.421	40.841	N	36.851	30.921	41.277	N	36.135	31.043	40.959	N	36.404	31.021	40.538
H	35.587	29.740	41.100	H	36.181	30.231	41.732	H	35.538	30.325	41.262	H	35.732	30.338	40.699
C	36.283	30.895	39.425	C	36.970	31.004	39.808	C	36.229	31.301	39.551	C	36.500	31.427	39.145
H	36.053	29.986	38.737	H	36.942	29.958	39.527	H	36.103	30.337	39.066	H	36.288	30.544	38.564
C	35.109	31.755	39.290	C	35.778	31.864	39.298	C	35.207	32.290	39.098	C	35.345	32.421	38.904
H	35.038	32.449	40.094	H	35.605	32.651	40.049	H	35.068	33.184	39.760	H	35.407	33.211	39.629
H	35.298	32.227	38.364	H	35.904	32.386	38.304	H	35.512	32.784	38.169	H	35.419	32.834	37.913
C	33.790	31.048	39.216	C	34.480	31.067	39.267	C	33.831	31.664	38.879	C	33.925	31.860	39.004
H	33.763	30.366	40.118	H	34.454	30.428	40.132	H	33.554	31.094	39.771	H	33.713	31.582	40.110
H	32.977	31.797	39.261	H	33.626	31.746	39.450	H	33.037	32.425	38.605	H	33.225	32.711	38.754
C	33.642	30.236	37.943	C	34.327	30.239	37.998	C	33.899	30.713	37.681	C	33.848	30.827	37.957
O	33.228	30.729	36.857	O	34.040	30.826	36.938	O	33.738	31.177	36.512	O	33.672	31.159	36.733
O	33.727	28.955	37.915	O	34.640	29.018	37.991	O	34.074	29.456	37.834	O	34.023	29.591	38.129
C	37.586	31.514	38.920	C	38.378	31.573	39.378	C	37.673	31.648	39.093	C	37.953	31.921	38.719
O	37.669	32.637	38.416	O	38.489	32.552	38.641	O	37.943	32.741	38.594	O	38.205	32.913	38.043
N	41.882	31.658	37.768	N	42.363	31.446	37.827	N	42.069	31.498	38.141	N	41.952	31.722	37.434
H	41.792	32.643	37.980	H	42.360	32.402	38.208	H	42.149	32.456	38.409	H	42.211	32.610	37.945
C	42.949	31.169	36.916	C	43.387	31.165	36.741	C	43.213	30.997	37.318	C	43.054	31.237	36.451

H	42.667	30.307	36.334	H	42.933	30.341	36.219	H	42.908	30.132	36.676	H	42.614	30.439	35.773
C	43.451	32.237	35.896	C	43.656	32.378	35.808	C	43.814	32.114	36.497	C	43.619	32.443	35.596
H	43.949	33.030	36.512	H	44.095	33.233	36.204	H	44.328	32.799	37.174	H	44.370	32.938	36.192
C	44.340	31.520	34.934	C	44.594	31.938	34.614	C	44.844	31.591	35.490	C	44.378	31.945	34.341
H	44.708	32.246	34.265	H	45.218	32.833	34.326	H	45.740	31.123	35.969	H	43.645	31.501	33.709
H	45.116	31.036	35.502	H	45.451	31.311	34.997	H	44.470	30.970	34.625	H	44.769	32.800	33.767
H	43.823	30.656	34.539	H	44.052	31.316	33.892	H	45.357	32.381	35.028	H	45.179	31.227	34.574
C	42.244	32.844	35.102	C	42.287	32.941	35.227	C	42.731	32.907	35.733	C	42.526	33.461	35.215
H	41.621	32.083	34.672	H	41.867	32.127	34.670	H	42.480	32.353	34.866	H	41.806	33.128	34.435
H	41.655	33.482	35.848	H	41.589	33.128	36.086	H	41.814	33.176	36.246	H	41.932	33.610	36.083
C	42.722	33.927	34.010	C	42.313	34.338	34.426	C	43.219	34.242	35.191	C	43.038	34.857	34.874
H	43.176	33.420	33.124	H	41.290	34.707	34.365	H	43.299	34.849	36.065	H	42.224	35.548	34.927
H	41.859	34.481	33.679	H	43.036	35.078	34.856	H	44.216	34.182	34.608	H	43.765	35.193	35.560
H	43.370	34.656	34.543	H	42.528	34.086	33.367	H	42.518	34.744	34.463	H	43.489	34.855	33.811
C	44.038	30.647	37.892	C	44.633	30.717	37.527	C	44.159	30.320	38.248	C	44.107	30.484	37.244
O	44.812	31.477	38.311	O	45.619	31.379	37.595	O	45.249	30.847	38.587	O	45.200	30.984	37.497
N	38.580	24.806	29.422	N	38.653	24.399	29.261	N	38.248	24.787	29.165	N	37.829	24.732	28.497
H	39.486	24.769	29.061	H	39.469	24.479	28.581	H	38.994	24.547	28.535	H	38.733	24.667	28.102
C	37.824	26.070	29.156	C	37.667	25.451	28.904	C	37.609	26.045	28.885	C	37.275	26.055	28.216
H	37.690	26.664	30.132	H	37.417	26.146	29.715	H	37.517	26.442	29.883	H	37.214	26.502	29.185
C	38.575	26.936	28.234	C	38.352	26.398	27.890	C	38.469	26.913	28.015	C	38.305	26.867	27.414
H	39.563	26.933	28.541	H	39.362	26.539	28.083	H	39.549	26.889	28.197	H	39.261	26.799	27.931
H	38.679	26.411	27.311	H	38.387	25.942	26.854	H	38.272	26.643	27.021	H	38.428	26.492	26.400
C	38.071	28.358	28.111	C	37.821	27.838	27.918	C	38.172	28.375	28.084	C	37.923	28.364	27.382
C	38.694	29.317	28.951	C	38.347	28.721	28.905	C	38.938	29.226	28.952	C	38.048	29.145	28.547
H	39.643	29.026	29.456	H	39.004	28.347	29.704	H	39.552	28.845	29.736	H	38.551	28.760	29.464
C	38.322	30.691	28.943	C	37.764	30.007	28.951	C	38.568	30.567	28.910	C	37.637	30.520	28.632
H	38.940	31.427	29.398	H	38.042	30.743	29.702	H	39.134	31.214	29.523	H	37.849	31.198	29.432

C	37.247	31.088	28.070	C	36.859	30.523	27.984	C	37.572	31.090	28.133	C	36.850	30.997	27.548
H	36.889	32.096	27.983	H	36.472	31.553	27.927	H	37.400	32.157	28.323	H	36.405	31.985	27.576
C	36.698	30.091	27.185	C	36.413	29.606	27.056	C	36.828	30.233	27.315	C	36.551	30.118	26.456
H	35.867	30.298	26.515	H	35.722	29.902	26.305	H	36.060	30.640	26.628	H	35.961	30.436	25.645
C	37.057	28.674	27.218	C	36.837	28.277	27.049	C	37.223	28.926	27.202	C	37.137	28.815	26.362
H	36.526	27.958	26.603	H	36.591	27.650	26.188	H	36.746	28.216	26.531	H	36.934	28.276	25.464
C	36.429	25.862	28.514	C	36.418	24.719	28.445	C	36.099	25.729	28.476	C	35.935	25.999	27.572
O	36.269	24.890	27.821	O	36.470	23.733	27.675	O	35.862	24.744	27.741	O	35.850	25.346	26.536
N	36.488	32.607	22.148	N	35.665	31.985	22.366	N	36.438	32.716	21.946	N	36.045	33.748	21.731
H	35.952	31.801	22.584	H	35.201	31.232	22.875	H	35.888	32.102	22.535	H	35.640	32.825	22.002
C	37.749	32.931	22.679	C	36.955	32.368	22.899	C	37.715	33.241	22.507	C	37.363	33.938	22.273
H	37.874	33.969	22.821	H	36.898	33.447	22.998	H	37.716	34.282	22.554	H	37.443	34.981	22.495
C	38.050	32.424	24.116	C	37.021	31.923	24.356	C	37.928	32.639	23.856	C	37.372	33.253	23.658
H	37.817	31.428	24.157	H	36.417	31.005	24.391	H	37.408	31.675	23.865	H	36.860	32.342	23.590
H	39.107	32.510	24.255	H	38.014	31.700	24.573	H	38.959	32.416	24.182	H	38.390	33.004	23.917
C	37.253	33.204	25.199	C	36.428	32.893	25.411	C	37.239	33.417	25.012	C	36.728	34.033	24.760
C	35.880	33.127	25.244	C	35.018	32.907	25.562	C	35.811	33.587	25.081	C	35.344	34.202	24.763
H	35.309	32.559	24.507	H	34.477	32.166	24.946	H	35.213	33.114	24.373	H	34.703	33.750	24.034
C	35.166	33.982	26.143	C	34.415	33.851	26.389	C	35.189	34.249	26.160	C	34.688	34.783	25.838
H	34.073	34.043	26.201	H	33.353	33.974	26.390	H	34.139	34.244	26.273	H	33.634	34.924	25.921
C	35.797	34.882	26.988	C	35.239	34.693	27.187	C	36.076	34.897	27.099	C	35.513	35.249	26.928
H	35.262	35.570	27.618	H	34.797	35.430	27.847	H	35.718	35.351	28.018	H	35.040	35.745	27.749
C	37.174	34.994	26.927	C	36.604	34.764	26.909	C	37.490	34.801	27.027	C	36.923	35.055	26.999
H	37.718	35.721	27.502	H	37.277	35.446	27.407	H	38.137	35.312	27.712	H	37.594	35.475	27.754
C	37.921	34.182	26.035	C	37.244	33.767	26.114	C	38.055	34.067	25.992	C	37.476	34.430	25.869
H	38.976	34.279	25.917	H	38.305	33.866	26.012	H	39.124	34.017	25.967	H	38.450	34.111	25.879
C	38.852	32.663	21.671	C	38.149	32.145	21.994	C	38.888	32.958	21.621	C	38.470	33.569	21.338
O	39.808	33.420	21.685	O	39.041	32.965	21.981	O	39.740	33.832	21.478	O	39.548	34.237	21.347

N	31.777	41.133	23.343	N	31.192	40.273	23.776	N	31.783	41.166	23.669	N	31.449	41.876	24.156
H	32.452	41.701	22.808	H	31.806	40.858	23.217	H	32.387	41.900	23.333	H	32.099	42.394	23.626
C	32.014	40.963	24.788	C	31.481	39.945	25.158	C	31.904	40.934	25.133	C	31.649	41.735	25.632
H	31.830	39.822	24.960	H	31.400	38.851	25.283	H	31.924	39.865	25.213	H	31.533	40.622	25.744
C	33.443	41.239	25.237	C	32.987	40.163	25.470	C	33.185	41.434	25.710	C	33.002	42.182	26.127
H	34.125	41.279	24.384	H	33.466	39.808	24.602	H	33.914	41.628	24.898	H	33.513	42.640	25.269
H	33.550	42.315	25.567	H	33.307	41.192	25.568	H	33.132	42.347	26.213	H	32.840	43.120	26.679
C	33.954	40.310	26.396	C	33.615	39.415	26.642	C	33.794	40.382	26.613	C	33.802	41.135	26.922
C	33.321	40.260	27.663	C	32.957	39.252	27.905	C	33.373	40.331	27.974	C	33.444	40.832	28.294
H	32.429	40.876	27.879	H	31.974	39.655	27.957	H	32.585	40.960	28.317	H	32.712	41.454	28.825
C	33.887	39.367	28.609	C	33.466	38.574	29.016	C	33.860	39.313	28.774	C	33.942	39.750	29.038
H	33.426	39.327	29.598	H	32.900	38.550	29.966	H	33.474	39.165	29.783	H	33.519	39.556	30.005
C	35.068	38.636	28.297	C	34.673	37.930	28.865	C	34.768	38.318	28.347	C	34.957	38.931	28.503
H	35.498	38.125	29.090	H	35.168	37.507	29.751	H	35.030	37.513	29.026	H	35.348	38.075	29.035
C	35.708	38.747	27.048	C	35.431	38.134	27.645	C	35.325	38.503	27.073	C	35.365	39.223	27.182
H	36.664	38.259	26.820	H	36.387	37.785	27.542	H	36.082	37.908	26.811	H	36.173	38.586	26.733
C	35.032	39.498	26.092	C	34.923	38.846	26.536	C	34.873	39.581	26.288	C	34.770	40.242	26.385
H	35.425	39.578	25.055	H	35.512	38.976	25.696	H	35.384	39.660	25.344	H	35.154	40.442	25.440
C	30.981	41.611	25.686	C	30.510	40.548	26.130	C	30.695	41.446	25.986	C	30.552	42.361	26.532
O	30.358	40.920	26.519	O	29.718	39.863	26.787	O	30.136	40.651	26.831	O	30.193	41.829	27.604
N	28.535	38.250	25.683	N	27.875	37.355	26.063	N	28.493	37.966	25.528	N	27.910	39.100	26.186
H	29.185	38.815	25.113	H	28.406	38.079	25.562	H	29.096	38.542	24.923	H	28.368	39.823	25.601
C	29.100	37.462	26.771	C	28.554	36.504	27.087	C	29.225	37.224	26.584	C	28.605	38.181	27.174
H	28.755	36.455	26.761	H	28.204	35.504	26.938	H	28.957	36.171	26.527	H	28.455	37.110	26.886
C	30.673	37.386	26.674	C	30.113	36.552	26.980	C	30.792	37.386	26.557	C	30.177	38.375	26.982
H	31.061	38.441	26.787	H	30.432	37.616	26.803	H	31.047	38.453	26.665	H	30.410	39.321	27.300
C	31.395	36.411	27.660	C	30.872	35.852	28.113	C	31.452	36.651	27.656	C	31.013	37.583	28.018
H	32.468	36.258	27.432	H	30.784	36.530	28.961	H	31.200	36.971	28.586	H	32.000	37.729	27.794

H	31.139	36.644	28.714	H	30.514	34.880	28.498	H	31.320	35.555	27.624	H	30.856	37.909	29.056
H	31.104	35.375	27.376	H	31.933	35.807	27.900	H	32.528	36.850	27.648	H	30.614	36.569	27.971
C	31.054	37.025	25.199	C	30.509	35.896	25.640	C	31.181	36.841	25.140	C	30.701	38.151	25.506
H	30.770	37.812	24.482	H	30.230	36.474	24.799	H	30.907	35.770	25.211	H	30.162	38.780	24.829
H	32.152	36.931	25.133	H	31.600	35.764	25.577	H	30.799	37.336	24.244	H	31.765	38.463	25.486
H	30.503	36.158	24.765	H	29.930	34.972	25.472	H	32.248	36.885	25.025	H	30.632	37.179	25.059
C	28.698	37.951	28.169	C	28.105	36.902	28.469	C	28.757	37.524	28.028	C	28.027	38.511	28.557
O	28.280	37.148	28.993	O	27.952	36.021	29.364	O	28.444	36.573	28.746	O	27.492	37.665	29.283
N	42.749	41.413	27.218	N	42.148	41.203	27.341	N	42.702	41.558	27.566	N	42.236	42.129	27.635
H	42.140	42.163	27.126	H	41.398	41.915	27.289	H	42.117	42.342	27.558	H	41.629	42.954	27.770
C	42.572	40.517	28.409	C	41.981	40.228	28.366	C	42.438	40.469	28.590	C	42.132	41.133	28.665
H	43.511	40.581	28.932	H	42.877	40.261	28.933	H	43.305	40.359	29.211	H	43.095	40.974	29.096
C	41.468	41.015	29.354	C	40.806	40.690	29.245	C	41.288	40.749	29.534	C	41.192	41.641	29.734
H	40.629	41.235	28.718	H	39.945	41.067	28.691	H	40.497	40.847	28.807	H	40.251	41.894	29.356
H	41.294	40.087	29.896	H	40.439	39.884	29.832	H	41.098	39.914	30.237	H	40.965	40.833	30.406
C	41.832	42.184	30.190	C	41.127	41.815	30.228	C	41.468	41.930	30.452	C	41.694	42.809	30.561
C	41.183	43.359	30.266	C	40.409	42.869	30.509	C	40.707	43.064	30.473	C	41.006	43.901	30.824
H	40.377	43.702	29.579	H	39.443	43.103	30.033	H	39.813	43.282	29.816	H	40.027	44.190	30.367
N	41.526	43.993	31.416	N	40.914	43.640	31.538	N	41.215	43.838	31.476	N	41.625	44.688	31.775
H	41.282	44.951	31.571	H	40.432	44.384	32.024	H	40.875	44.710	31.757	H	41.155	45.536	32.118
C	42.425	43.316	32.154	C	42.001	43.002	32.058	C	42.319	43.343	32.143	C	42.668	43.993	32.304
C	43.250	43.700	33.191	C	42.845	43.285	33.124	C	43.203	43.771	33.190	C	43.318	44.168	33.528
H	43.008	44.622	33.766	H	42.642	44.140	33.729	H	43.048	44.640	33.710	H	43.147	45.056	34.152
C	44.159	42.707	33.650	C	43.731	42.298	33.533	C	44.221	42.888	33.663	C	44.244	43.135	33.905
H	44.544	42.860	34.621	H	44.252	42.411	34.498	H	44.863	43.183	34.455	H	44.831	43.264	34.819
C	44.340	41.493	32.923	C	43.904	41.133	32.829	C	44.375	41.688	33.055	C	44.465	42.064	33.064
H	45.109	40.835	33.268	H	44.590	40.393	33.218	H	45.232	41.031	33.309	H	45.089	41.289	33.307
C	43.668	41.243	31.725	C	43.126	40.925	31.681	C	43.533	41.299	31.949	C	43.783	41.899	31.813

H	43.903	40.387	31.091	H	43.229	40.011	31.111	H	43.780	40.450	31.314	H	43.863	41.010	31.188
C	42.667	42.152	31.332	C	42.152	41.843	31.262	C	42.473	42.117	31.516	C	42.817	42.879	31.476
C	42.422	39.024	27.970	C	41.881	38.776	27.889	C	42.359	39.054	27.946	C	41.801	39.693	28.165
O	43.308	38.237	28.314	O	42.763	38.005	28.207	O	43.125	38.177	28.421	O	42.614	38.774	28.390
N	41.509	38.668	27.057	N	40.992	38.391	26.980	N	41.527	38.707	26.979	N	40.787	39.395	27.267
C	40.335	39.465	26.648	C	39.778	39.060	26.726	C	40.357	39.478	26.562	C	39.750	40.299	26.898
H	40.736	40.378	26.156	H	39.999	39.991	26.277	H	40.498	40.450	26.112	H	40.201	41.224	26.540
H	39.845	39.776	27.552	H	39.193	39.236	27.672	H	39.780	39.704	27.400	H	39.099	40.535	27.721
C	39.354	38.615	25.866	C	38.956	38.112	25.744	C	39.403	38.616	25.775	C	38.982	39.619	25.814
H	38.835	39.178	25.134	H	38.619	38.687	24.898	H	38.995	39.132	24.886	H	39.355	39.921	24.858
H	38.757	38.154	26.634	H	38.192	37.575	26.320	H	38.547	38.188	26.327	H	37.910	39.868	25.947
C	40.288	37.588	25.275	C	39.940	37.131	25.159	C	40.276	37.456	25.353	C	39.212	38.106	25.965
H	40.696	37.973	24.350	H	40.385	37.575	24.295	H	40.445	37.381	24.292	H	39.165	37.618	25.014
H	39.840	36.653	25.152	H	39.466	36.213	24.912	H	39.773	36.552	25.631	H	38.495	37.603	26.708
C	41.429	37.451	26.357	C	40.982	37.074	26.225	C	41.483	37.458	26.267	C	40.666	38.148	26.565
H	41.116	36.812	27.149	H	40.759	36.346	27.035	H	41.305	36.699	26.980	H	40.554	37.446	27.370
C	42.722	36.965	25.744	C	42.305	36.762	25.679	C	42.814	37.044	25.565	C	41.771	37.794	25.531
O	43.035	35.789	25.806	O	42.695	35.581	25.689	O	43.142	35.847	25.395	O	41.808	36.544	25.240
N	45.227	35.583	28.508	N	45.001	35.420	28.303	N	45.270	35.419	28.115	N	44.747	36.259	28.212
H	44.479	36.186	28.073	H	44.311	35.854	27.714	H	44.544	35.941	27.672	H	44.056	36.746	27.701
C	44.939	34.293	29.191	C	44.673	34.197	29.040	C	44.953	34.178	28.831	C	44.516	35.056	29.042
H	44.785	34.549	30.252	H	44.728	34.539	30.110	H	44.791	34.499	29.863	H	44.414	35.415	30.101
C	43.603	33.634	28.678	C	43.324	33.512	28.668	C	43.618	33.583	28.305	C	43.147	34.329	28.825
H	43.778	33.285	27.615	H	43.297	33.243	27.630	H	43.711	33.389	27.291	H	43.258	33.889	27.865
C	43.220	32.521	29.677	C	43.186	32.170	29.482	C	43.259	32.365	29.154	C	42.763	33.248	29.902
H	42.860	32.917	30.661	H	43.527	32.382	30.439	H	42.365	31.888	28.792	H	41.886	32.753	29.481
H	42.362	32.020	29.287	H	42.183	31.687	29.455	H	44.115	31.686	29.138	H	43.545	32.522	30.211
H	44.027	31.789	29.576	H	43.816	31.459	28.980	H	43.085	32.620	30.162	H	42.438	33.858	30.701

C	42.437	34.627	28.900	C	42.113	34.452	28.834	C	42.453	34.553	28.523	C	42.107	35.375	28.622
H	42.407	34.874	29.895	H	42.030	34.775	29.813	H	42.362	34.778	29.628	H	42.143	35.941	29.531
H	42.663	35.399	28.281	H	42.246	35.262	28.167	H	42.672	35.479	27.946	H	42.365	36.142	27.885
C	41.097	34.138	28.327	C	40.777	33.908	28.303	C	41.041	34.238	27.971	C	40.697	34.944	28.239
H	40.451	34.990	28.195	H	40.706	32.812	28.426	H	40.373	35.008	28.294	H	40.763	34.437	27.274
H	41.227	33.656	27.316	H	39.979	34.466	28.784	H	41.015	34.062	26.930	H	40.253	34.209	28.995
H	40.630	33.460	28.970	H	40.702	34.144	27.218	H	40.743	33.326	28.460	H	40.104	35.856	28.055
C	46.046	33.197	29.024	C	45.856	33.139	28.861	C	46.047	33.174	28.702	C	45.666	33.988	28.680
O	46.442	32.868	27.857	O	46.160	32.909	27.681	O	46.463	32.849	27.566	O	45.908	33.837	27.497
N	35.005	28.845	35.184	N	35.563	28.585	35.208	N	35.424	29.076	35.256	N	34.982	29.564	34.838
H	34.552	29.583	35.729	H	35.119	29.370	35.696	H	34.940	29.758	35.873	H	34.618	30.142	35.536
C	36.473	28.778	35.410	C	37.048	28.534	35.176	C	36.895	28.948	35.397	C	36.448	29.308	34.804
H	36.894	28.455	34.475	H	37.487	28.327	34.173	H	37.331	28.444	34.468	H	36.776	28.995	33.831
C	36.957	30.188	35.633	C	37.685	29.894	35.501	C	37.662	30.287	35.598	C	37.082	30.655	35.155
H	36.486	30.684	36.482	H	37.591	30.084	36.528	H	37.701	30.468	36.663	H	36.675	31.048	36.077
H	38.028	30.138	36.035	H	38.769	29.892	35.426	H	38.591	30.057	35.181	H	38.076	30.339	35.377
C	36.934	31.069	34.355	C	37.012	31.033	34.653	C	36.951	31.481	34.768	C	37.025	31.650	33.996
H	35.936	31.295	34.041	H	35.963	30.986	34.617	H	35.980	31.647	35.200	H	36.005	31.957	33.752
C	37.515	32.458	34.704	C	37.426	32.396	35.237	C	37.869	32.735	35.068	C	37.763	32.905	34.575
H	38.581	32.508	34.959	H	38.472	32.630	35.025	H	38.925	32.412	35.062	H	38.816	32.769	34.713
H	37.254	33.064	33.909	H	36.811	33.118	34.814	H	37.795	33.588	34.397	H	37.640	33.657	33.823
H	37.037	32.950	35.498	H	37.220	32.281	36.311	H	37.646	33.033	36.086	H	37.235	33.306	35.381
C	37.627	30.512	33.085	C	37.404	30.948	33.210	C	36.853	31.238	33.255	C	37.856	31.141	32.735
H	38.670	30.204	33.151	H	38.367	30.754	33.073	H	36.330	30.299	33.063	H	37.395	30.400	32.189
H	36.997	29.689	32.724	H	36.799	30.103	32.753	H	36.274	31.994	32.806	H	37.981	31.918	31.937
H	37.635	31.347	32.311	H	37.060	31.826	32.653	H	37.811	31.158	32.788	H	38.789	30.741	33.087
C	36.909	27.822	36.611	C	37.593	27.516	36.257	C	37.277	27.949	36.519	C	36.961	28.284	35.844
O	38.074	27.479	36.716	O	38.764	27.244	36.437	O	38.351	27.431	36.576	O	38.140	27.839	35.831

N	29.011	32.377	36.969	N	29.847	31.943	37.704	N	29.451	32.525	36.883	N	28.933	33.094	36.715
H	28.361	32.940	37.407	H	29.296	32.288	38.480	H	28.731	32.851	37.480	H	28.183	33.572	37.241
C	30.433	32.837	37.060	C	31.140	32.699	37.620	C	30.702	33.198	36.921	C	30.380	33.364	37.015
H	30.947	31.994	37.476	H	31.880	32.046	38.051	H	31.320	32.388	37.267	H	30.759	32.479	37.561
C	31.069	33.167	35.646	C	31.589	32.836	36.146	C	31.299	33.729	35.632	C	31.108	33.624	35.685
H	31.271	32.197	35.081	H	31.804	31.761	35.753	H	31.469	32.790	35.038	H	31.353	32.626	35.172
H	30.477	33.745	34.970	H	30.799	33.298	35.581	H	30.639	34.376	35.067	H	30.450	34.258	35.069
C	32.325	33.915	35.812	C	32.753	33.751	35.975	C	32.554	34.459	35.781	C	32.442	34.357	35.707
N	33.521	33.430	36.407	N	34.049	33.439	36.387	N	33.782	33.828	36.034	N	33.657	33.796	36.085
H	33.635	32.446	36.778	H	34.242	32.560	36.821	H	33.879	32.812	36.148	H	33.871	32.889	36.571
C	34.414	34.439	36.545	C	34.782	34.604	36.462	C	34.765	34.802	36.177	C	34.677	34.762	35.966
H	35.421	34.351	36.916	H	35.840	34.641	36.831	H	35.876	34.733	36.363	H	35.733	34.581	36.243
N	33.879	35.571	35.987	N	34.007	35.586	35.928	N	34.190	35.999	36.147	N	34.055	35.892	35.518
C	32.581	35.255	35.548	C	32.773	35.069	35.659	C	32.859	35.817	35.846	C	32.697	35.691	35.323
H	31.840	35.873	35.028	H	31.990	35.694	35.323	H	32.098	36.567	35.892	H	32.039	36.571	35.129
C	30.549	34.065	37.994	C	31.153	34.125	38.313	C	30.696	34.276	37.983	C	30.469	34.533	37.977
O	31.403	34.024	38.861	O	31.999	34.468	39.138	O	31.470	34.282	38.896	O	31.350	34.555	38.828
C	39.516	35.301	32.172	C	39.633	34.219	31.827	C	40.272	35.243	32.053	C	39.663	35.729	32.022
H	39.718	36.244	32.675	H	39.738	34.074	30.801	H	40.585	35.889	32.860	H	39.480	34.821	31.463
H	40.261	35.197	31.368	H	39.866	33.296	32.377	H	40.940	35.524	31.244	H	40.365	35.252	32.745
H	39.596	34.421	32.832	H	40.540	34.827	32.144	H	40.371	34.222	32.439	H	40.141	36.496	31.304
C	38.079	35.320	31.597	C	38.325	34.737	32.343	C	38.776	35.663	31.745	C	38.333	36.300	32.396
H	37.494	35.048	32.419	H	38.183	34.435	33.367	H	38.218	35.590	32.792	C	37.962	37.675	31.952
C	37.689	36.717	31.063	C	38.308	36.207	32.618	C	38.640	37.069	31.306	H	38.052	38.320	32.892
H	37.695	37.429	31.847	H	39.185	36.737	32.841	H	39.129	37.687	32.031	H	36.925	37.706	31.605
H	36.717	36.751	30.634	H	37.463	36.796	32.413	H	37.663	37.476	31.130	CL	37.024	35.201	30.733
CL	38.028	34.165	30.307	CL	37.033	34.286	31.269	CL	38.144	34.505	30.574	CL	38.938	38.400	30.628
CL	38.995	37.147	29.902	CL	38.692	36.850	30.468	CL	39.385	37.222	29.697	N	37.942	41.428	34.353

Table S9. Cartesian coordinates (Å) of the LinB first-shell amino acids in complex with the (*R*)-DCP, shown for the representative structure of the largest cluster corresponding to the stationary points of the dehalogenation step. Provided geometries were used as the reference model for SAPT analysis of noncovalent interactions.

**C1**								**C2**							
**RC**				**TS1**				**RC**				**TS1**			
Atom	X	Y	Z	Atom	X	Y	Z	Atom	X	Y	Z	Atom	X	Y	Z
N	34.216	39.661	32.614	N	34.436	39.382	32.602	N	34.290	39.268	32.394	N	33.767	39.050	31.814
H	33.633	38.904	32.182	H	34.327	38.555	32.177	H	33.476	38.715	32.601	H	33.092	38.340	31.748
C	34.182	40.905	31.903	C	34.386	40.686	31.844	C	34.114	40.340	31.441	C	33.674	40.254	30.911
H	34.946	41.521	32.319	H	35.138	41.273	32.323	H	35.020	40.864	31.375	H	34.611	40.813	30.837
C	34.709	40.734	30.442	C	34.759	40.224	30.404	C	33.885	39.657	30.117	C	33.218	39.631	29.629
H	35.707	40.430	30.526	H	35.800	39.888	30.341	H	34.726	38.910	29.920	H	33.846	38.726	29.349
H	34.345	39.899	29.872	H	34.096	39.434	30.083	H	32.953	39.119	30.216	H	32.219	39.197	29.752
C	34.573	41.928	29.525	C	34.408	41.334	29.438	C	33.675	40.645	29.024	C	33.362	40.668	28.530
O	34.988	43.022	29.802	O	34.878	42.457	29.551	O	34.473	41.520	28.610	O	33.991	41.690	28.533
N	33.937	41.879	28.366	N	33.499	41.110	28.504	N	32.506	40.725	28.539	N	32.598	40.389	27.534
H	33.677	42.731	27.916	H	33.384	41.847	27.845	H	32.269	41.504	27.970	H	32.621	40.985	26.704
H	33.455	41.037	28.014	H	33.309	40.135	28.281	H	31.737	40.088	28.806	H	31.949	39.610	27.656
C	32.811	41.621	32.026	C	32.984	41.336	31.968	C	32.998	41.340	31.779	C	32.547	41.232	31.451
O	31.754	41.046	31.824	O	31.979	40.562	31.941	O	31.891	40.901	32.185	O	31.530	40.760	31.825
N	31.553	33.687	34.658	N	31.723	33.644	34.604	N	31.034	34.693	35.611	N	31.118	34.440	35.965
H	30.561	33.497	34.908	H	30.772	33.354	34.837	H	30.089	34.877	35.974	H	30.220	34.316	36.418
C	31.797	34.270	33.335	C	31.968	34.171	33.187	C	31.205	34.911	34.144	C	31.001	34.589	34.418
H	31.052	33.845	32.613	H	31.219	33.590	32.568	H	30.234	34.561	33.733	H	30.120	34.088	34.161
C	31.491	35.838	33.506	C	31.600	35.650	33.159	C	31.208	36.459	33.849	C	30.860	36.138	34.118
H	30.461	35.939	33.898	H	30.498	35.714	33.515	H	30.359	36.907	34.364	H	30.002	36.385	34.776
H	32.132	36.262	34.214	H	32.093	36.243	33.937	H	32.073	36.944	34.362	H	31.675	36.632	34.566

C	31.538	36.627	32.199	C	31.929	36.304	31.813	C	31.203	36.804	32.341	C	30.748	36.476	32.629
O	30.787	36.368	31.205	O	31.043	36.194	30.878	O	30.337	36.389	31.507	O	29.688	36.159	31.982
O	32.318	37.619	32.052	O	32.972	36.955	31.689	O	32.198	37.298	31.835	O	31.640	37.109	32.011
C	33.217	33.875	32.717	C	33.314	33.736	32.641	C	32.337	34.130	33.405	C	32.066	33.814	33.637
O	33.655	32.698	32.832	O	33.494	32.528	32.803	O	32.465	32.954	33.819	O	32.382	32.641	33.964
N	34.015	34.778	32.302	N	34.281	34.511	32.129	N	33.033	34.625	32.388	N	32.859	34.394	32.727
H	33.641	35.775	32.241	H	34.034	35.465	31.951	H	32.705	35.452	31.932	H	32.680	35.386	32.498
C	35.406	34.557	31.825	C	35.688	34.124	31.696	C	34.115	33.919	31.747	C	33.928	33.680	32.030
H	35.322	33.738	31.077	H	35.655	33.341	30.965	H	33.750	33.075	31.219	H	33.538	32.724	31.593
C	35.900	35.871	31.153	C	36.364	35.278	30.923	C	34.816	34.834	30.729	C	34.590	34.549	31.017
H	35.694	36.784	31.745	H	36.292	36.206	31.482	H	34.783	35.883	31.126	H	34.663	35.607	31.292
H	37.007	35.700	31.196	H	37.406	35.187	30.824	H	35.882	34.558	30.606	H	35.638	34.213	30.964
C	35.517	35.998	29.695	C	35.823	35.388	29.588	C	34.125	34.717	29.448	C	33.927	34.455	29.685
C	34.856	36.988	29.077	C	35.025	36.364	29.076	C	33.563	35.729	28.791	C	33.327	35.416	28.966
H	34.499	37.947	29.518	H	34.619	37.285	29.497	H	33.479	36.750	29.184	H	33.288	36.430	29.207
N	34.628	36.625	27.764	N	34.739	36.051	27.727	N	33.028	35.338	27.627	N	32.781	34.926	27.815
H	34.084	37.143	27.119	H	34.119	36.500	27.168	H	32.271	35.850	27.122	H	32.179	35.488	27.256
C	35.236	35.493	27.419	C	35.449	34.953	27.333	C	33.312	34.014	27.366	C	33.036	33.585	27.655
C	35.578	34.944	26.162	C	35.737	34.412	26.051	C	33.190	33.258	26.243	C	32.842	32.670	26.673
H	35.255	35.437	25.259	H	35.263	34.907	25.218	H	32.732	33.701	25.344	H	32.235	32.986	25.792
C	36.372	33.816	26.192	C	36.533	33.261	25.959	C	33.581	31.897	26.364	C	33.336	31.346	26.846
H	36.748	33.228	25.345	H	36.710	32.879	24.934	H	33.341	31.173	25.631	H	33.194	30.649	26.025
C	36.698	33.183	27.395	C	37.073	32.682	27.091	C	34.210	31.407	27.488	C	34.135	31.045	27.955
H	37.082	32.162	27.321	H	37.579	31.748	26.976	H	34.558	30.413	27.379	H	34.701	30.187	28.034
C	36.580	33.878	28.631	C	36.907	33.381	28.267	C	34.542	32.288	28.518	C	34.448	32.036	28.897
H	36.910	33.413	29.556	H	37.415	32.956	29.088	H	35.175	31.959	29.349	H	35.198	31.890	29.701
C	35.778	35.041	28.650	C	36.092	34.492	28.475	C	34.051	33.605	28.529	C	33.835	33.334	28.793
C	36.347	34.062	32.926	C	36.570	33.762	32.910	C	35.154	33.392	32.706	C	35.048	33.164	33.051

O	37.265	33.265	32.807	O	37.525	33.024	32.752	O	35.886	32.491	32.332	O	35.678	32.112	32.752
N	26.487	31.334	36.926	N	26.921	31.006	36.716	N	26.396	33.132	38.863	N	26.462	32.705	38.456
H	25.590	30.880	37.120	H	26.169	30.355	36.937	H	25.561	32.958	39.483	H	25.633	32.349	38.955
C	26.522	32.015	35.577	C	26.852	31.732	35.377	C	26.191	33.718	37.525	C	26.204	33.330	37.178
H	25.681	31.527	34.934	H	26.201	31.092	34.770	H	25.225	33.269	37.131	H	25.312	32.833	36.863
C	26.028	33.451	35.647	C	26.196	33.086	35.650	C	25.869	35.228	37.695	C	25.853	34.807	37.412
H	26.496	33.834	36.480	H	26.399	33.443	36.706	H	26.440	35.575	38.542	H	26.699	35.282	37.999
H	26.327	34.035	34.728	H	26.634	33.772	34.905	H	26.009	35.818	36.842	H	25.887	35.201	36.428
C	24.532	33.549	35.872	C	24.658	32.970	35.685	C	24.374	35.421	38.107	C	24.464	35.075	37.986
H	24.172	32.788	36.578	H	24.410	32.208	36.408	H	24.070	34.620	38.839	H	24.202	34.388	38.774
H	24.331	34.621	36.112	H	24.223	33.931	36.091	H	24.294	36.360	38.642	H	24.493	36.077	38.475
C	23.776	33.370	34.525	C	23.967	32.808	34.325	C	23.470	35.268	36.844	C	23.340	35.182	36.920
O	23.818	34.280	33.626	O	23.792	33.777	33.528	O	23.598	35.857	35.730	O	23.333	36.063	35.997
O	23.111	32.380	34.184	O	23.424	31.730	33.975	O	22.551	34.428	36.868	O	22.446	34.287	36.807
C	27.901	31.914	34.905	C	28.181	31.765	34.563	C	27.331	33.360	36.562	C	27.419	33.028	36.282
O	28.766	32.817	34.858	O	28.963	32.744	34.638	O	28.222	34.161	36.267	O	28.214	33.817	35.826
N	30.881	29.733	32.046	N	31.239	29.672	31.755	N	29.169	30.219	33.457	N	29.445	29.910	33.597
H	31.519	30.262	32.621	H	31.967	30.174	32.285	H	30.121	30.582	33.629	H	30.337	30.342	33.659
C	31.308	29.205	30.678	C	31.567	29.090	30.430	C	29.001	29.536	32.129	C	29.281	29.036	32.406
H	30.454	29.223	29.980	H	30.716	29.078	29.886	H	28.060	29.826	31.712	H	28.214	29.009	32.141
C	32.439	30.009	30.059	C	32.506	30.002	29.677	C	30.122	29.943	31.116	C	30.097	29.589	31.228
H	33.186	30.009	30.770	H	33.330	30.131	30.311	H	31.039	29.583	31.663	H	31.089	29.562	31.566
C	32.897	29.483	28.674	C	32.919	29.341	28.318	C	29.885	29.361	29.711	C	29.949	28.740	29.959
H	32.947	28.399	28.690	H	33.374	30.105	27.650	H	30.820	29.407	29.106	H	28.977	28.906	29.561
H	32.392	29.881	27.823	H	33.495	28.400	28.371	H	29.557	28.384	29.645	H	30.816	28.864	29.280
H	33.972	29.623	28.583	H	32.025	29.053	27.710	H	29.070	29.810	29.213	H	29.921	27.710	30.259
C	32.059	31.498	30.033	C	31.928	31.392	29.395	C	30.113	31.559	31.042	C	29.826	31.127	30.899
H	31.067	31.581	29.641	H	31.035	31.306	28.825	H	29.143	31.929	30.774	H	28.831	31.228	30.487

H	32.077	31.961	31.027	H	31.696	31.759	30.377	H	30.528	31.980	31.994	H	29.865	31.639	31.817
C	32.964	32.286	29.135	C	33.090	32.357	28.812	C	31.123	32.145	30.088	C	30.872	31.782	29.877
H	32.862	31.935	28.113	H	34.044	31.990	29.110	H	32.109	32.014	30.548	H	31.827	32.012	30.374
H	32.716	33.341	29.198	H	33.091	32.333	27.661	H	31.049	31.642	29.150	H	31.155	31.085	29.146
H	34.000	32.140	29.488	H	32.921	33.390	29.117	H	31.114	33.201	29.955	H	30.503	32.713	29.406
C	31.616	27.686	30.975	C	32.109	27.648	30.686	C	29.033	28.063	32.412	C	29.626	27.641	32.803
O	32.740	27.222	30.917	O	33.350	27.422	30.693	O	30.052	27.373	32.156	O	30.695	27.097	32.576
N	21.563	31.805	24.353	N	21.827	31.413	24.345	N	18.749	33.750	27.361	N	18.600	33.629	28.032
H	22.242	31.338	23.742	H	22.314	31.032	23.505	H	18.770	33.458	26.437	H	18.868	33.219	27.169
C	21.800	33.122	24.721	C	21.944	32.835	24.611	C	19.330	35.034	27.800	C	18.715	35.058	28.321
H	22.271	33.133	25.697	H	22.313	33.032	25.628	H	19.717	34.904	28.798	H	19.221	35.191	29.271
C	22.760	33.684	23.700	C	22.922	33.366	23.598	C	20.310	35.431	26.671	C	19.481	35.729	27.185
H	23.522	32.850	23.515	H	23.592	32.584	23.254	H	20.808	34.563	26.216	H	19.050	35.369	26.244
H	22.336	33.883	22.711	H	22.278	33.574	22.732	H	19.677	35.859	25.908	H	19.363	36.861	27.192
C	23.407	34.901	24.136	C	23.746	34.534	24.100	C	21.257	36.443	27.069	C	20.932	35.454	27.227
C	24.663	34.812	24.876	C	24.699	34.246	25.012	C	22.384	36.169	27.864	C	21.445	34.295	26.625
H	25.070	33.850	24.913	H	24.961	33.168	25.223	H	22.681	35.174	28.090	H	20.756	33.530	26.312
C	25.335	36.001	25.318	C	25.404	35.305	25.694	C	23.241	37.225	28.220	C	22.827	34.045	26.634
H	26.350	35.902	25.752	H	26.172	35.068	26.387	H	24.156	37.060	28.749	H	23.136	33.060	26.220
C	24.878	37.217	24.840	C	25.060	36.636	25.386	C	22.987	38.512	27.790	C	23.725	35.024	27.021
H	25.361	38.079	25.112	H	25.510	37.462	25.883	H	23.618	39.316	28.115	H	24.720	34.756	26.978
C	23.675	37.258	24.136	C	24.040	36.924	24.446	C	21.819	38.849	27.034	C	23.216	36.229	27.585
H	23.333	38.240	23.944	H	23.845	37.956	24.233	H	21.525	39.884	26.813	H	23.923	36.948	27.991
C	22.962	36.135	23.765	C	23.350	35.852	23.919	C	21.007	37.759	26.646	C	21.812	36.438	27.656
H	21.953	36.211	23.383	H	22.488	36.027	23.232	H	20.015	38.035	26.187	H	21.406	37.334	28.083
C	20.503	33.977	24.681	C	20.599	33.538	24.478	C	18.273	36.096	27.963	C	17.312	35.729	28.543
O	19.618	33.726	23.812	O	19.873	33.171	23.549	O	17.230	36.023	27.277	O	16.324	35.347	27.919
N	25.509	41.331	21.235	N	25.866	41.205	21.421	N	24.112	41.881	23.126	N	23.562	41.122	23.142

H	24.623	40.843	21.517	H	25.023	40.723	21.717	H	23.251	41.782	23.681	H	22.750	40.779	23.603
C	26.768	40.561	21.323	C	27.118	40.425	21.584	C	25.102	40.816	23.181	C	24.673	40.221	23.080
H	27.451	41.241	21.802	H	27.956	41.048	21.898	H	26.108	41.251	23.258	H	25.580	40.764	23.149
C	26.650	39.368	22.335	C	26.875	39.346	22.632	C	24.737	39.933	24.381	C	24.570	39.215	24.278
H	25.757	38.918	22.220	H	25.971	38.905	22.405	H	23.694	40.000	24.469	H	23.714	38.657	24.080
H	27.399	38.613	22.092	H	27.609	38.626	22.390	H	25.174	38.959	24.184	H	25.404	38.532	24.214
C	26.895	39.871	23.703	C	27.002	39.684	24.053	C	25.237	40.434	25.716	C	24.481	39.786	25.705
C	28.150	39.730	24.325	C	28.338	39.966	24.560	C	26.575	40.132	26.145	C	25.564	40.354	26.362
H	28.941	39.067	23.868	H	29.269	39.811	23.973	H	27.087	39.448	25.551	H	26.427	40.582	25.801
C	28.470	40.385	25.533	C	28.544	40.305	25.911	C	26.937	40.511	27.433	C	25.484	40.820	27.640
H	29.536	40.294	25.877	H	29.506	40.405	26.339	H	27.914	40.244	27.767	H	26.365	41.303	28.080
C	27.421	41.050	26.218	C	27.424	40.581	26.699	C	26.138	41.340	28.224	C	24.268	40.640	28.333
H	27.598	41.639	27.071	H	27.570	41.028	27.720	H	26.430	41.725	29.175	H	24.239	40.880	29.374
C	26.176	41.164	25.618	C	26.099	40.516	26.168	C	24.889	41.702	27.745	C	23.142	40.046	27.767
H	25.363	41.734	26.070	H	25.225	40.782	26.697	H	24.235	42.390	28.336	H	22.210	40.017	28.296
C	25.966	40.618	24.364	C	25.949	40.072	24.789	C	24.443	41.296	26.457	C	23.335	39.629	26.454
H	25.015	40.660	23.885	H	24.973	40.067	24.374	H	23.514	41.565	26.165	H	22.489	39.143	26.030
C	27.381	40.091	19.964	C	27.674	39.903	20.275	C	25.209	40.054	21.859	C	24.867	39.647	21.712
O	28.607	40.036	19.871	O	28.927	40.033	20.000	O	26.380	39.746	21.419	O	25.932	39.687	21.137
N	28.800	48.757	26.622	N	28.616	48.398	26.222	N	28.989	48.212	25.844	N	28.283	47.871	26.095
H	29.531	48.922	25.894	H	29.386	48.627	25.598	H	29.604	48.193	25.008	H	28.838	47.959	25.268
C	29.101	47.688	27.589	C	28.754	47.271	27.213	C	29.301	47.142	26.874	C	28.544	46.803	27.107
H	28.470	46.862	27.372	H	27.901	46.612	26.945	H	28.357	46.596	27.048	H	27.631	46.178	27.146
C	30.583	47.187	27.480	C	30.080	46.574	26.997	C	30.280	46.081	26.329	C	29.796	46.018	26.754
H	31.064	47.536	26.538	H	30.339	46.587	25.877	H	30.004	46.032	25.274	H	29.945	46.027	25.660
H	31.218	47.619	28.259	H	30.837	47.085	27.519	H	31.303	46.263	26.496	H	30.692	46.581	27.142
C	30.566	45.665	27.485	C	30.332	45.099	27.379	C	30.063	44.730	27.004	C	29.800	44.518	27.237
C	30.703	44.950	26.282	C	31.418	44.438	26.806	C	30.267	43.593	26.237	C	29.956	43.586	26.190

H	30.806	45.524	25.365	H	32.064	45.019	26.166	H	30.594	43.678	25.183	H	30.267	43.927	25.168
C	30.504	43.577	26.204	C	31.646	43.075	26.920	C	30.087	42.299	26.785	C	29.972	42.188	26.427
H	30.385	43.070	25.261	H	32.355	42.523	26.317	H	30.260	41.433	26.183	H	30.215	41.544	25.686
C	30.175	42.893	27.432	C	30.889	42.405	27.899	C	29.851	42.164	28.190	C	29.765	41.665	27.752
H	29.931	41.814	27.438	H	30.913	41.278	28.011	H	29.670	41.203	28.627	H	29.664	40.636	27.908
C	30.172	43.559	28.676	C	29.700	43.009	28.463	C	29.699	43.367	28.948	C	29.458	42.604	28.781
H	30.053	43.036	29.595	H	28.937	42.451	28.942	H	29.517	43.228	30.044	H	29.259	42.267	29.748
C	30.367	44.922	28.663	C	29.450	44.363	28.204	C	29.906	44.602	28.396	C	29.468	44.017	28.529
H	30.353	45.398	29.591	H	28.610	44.939	28.557	H	29.839	45.446	29.056	H	29.197	44.737	29.293
C	28.905	48.063	29.027	C	28.622	47.681	28.697	C	29.522	47.754	28.240	C	28.739	47.389	28.510
O	28.527	47.284	29.868	O	27.850	47.091	29.457	O	28.781	47.509	29.136	O	28.063	46.924	29.466
N	24.657	47.783	28.846	N	24.359	47.874	28.593	N	25.512	48.518	29.175	N	24.640	48.285	29.143
H	25.264	48.148	28.096	H	25.229	48.364	28.331	H	26.222	48.576	28.431	H	25.553	48.484	28.806
C	24.685	46.323	29.137	C	24.329	46.402	28.835	C	25.133	47.169	29.688	C	24.319	46.928	29.566
H	23.674	45.854	28.929	H	23.328	46.003	28.667	H	24.071	47.166	29.659	H	23.245	46.897	29.682
C	25.682	45.612	28.175	C	25.275	45.544	27.949	C	25.746	46.060	28.709	C	24.879	46.017	28.458
H	26.686	45.937	28.338	H	26.247	45.924	28.195	H	26.677	46.471	28.323	H	25.938	46.389	28.323
C	25.704	44.060	28.245	C	25.423	43.995	28.335	C	26.294	44.801	29.431	C	24.856	44.559	28.868
H	25.921	43.629	29.196	H	26.255	43.678	27.756	H	26.938	45.065	30.226	H	25.300	44.401	29.839
H	24.754	43.701	27.875	H	25.563	43.837	29.435	H	25.537	44.191	29.809	H	23.808	44.339	28.888
H	26.456	43.689	27.560	H	24.561	43.436	27.928	H	26.862	44.156	28.744	H	25.355	43.935	28.098
C	25.294	45.859	26.709	C	25.148	45.747	26.488	C	24.730	45.746	27.551	C	24.085	46.174	27.199
H	25.520	46.866	26.428	H	25.229	46.760	26.242	H	24.849	44.806	27.101	H	24.609	45.542	26.477
H	25.810	45.185	26.051	H	25.988	45.225	26.042	H	23.738	45.832	27.978	H	23.052	45.841	27.327
H	24.182	45.734	26.592	H	24.213	45.304	26.027	H	24.805	46.628	26.929	H	24.046	47.173	26.768
C	24.964	46.070	30.668	C	24.496	46.054	30.316	C	25.425	47.018	31.186	C	24.827	46.679	30.976
O	24.333	45.220	31.215	O	23.686	45.240	30.734	O	24.691	46.317	31.895	O	24.079	46.232	31.821
N	37.227	40.321	24.174	N	37.548	39.868	24.598	N	35.412	38.409	23.736	N	35.009	38.170	23.518

H	37.615	41.179	24.724	H	37.685	40.703	25.197	H	35.654	39.315	24.110	H	35.433	38.988	23.922
C	36.519	39.240	24.837	C	36.747	38.800	25.132	C	34.632	37.517	24.552	C	34.321	37.209	24.445
H	37.270	38.425	24.814	H	37.373	37.917	25.269	H	35.218	36.631	24.473	H	34.918	36.282	24.447
C	36.135	39.716	26.295	C	36.334	39.146	26.568	C	34.581	38.101	25.990	C	34.251	37.807	25.881
H	35.833	40.777	26.410	H	35.831	40.087	26.579	H	34.462	39.250	25.923	H	34.141	38.892	25.834
H	35.291	39.132	26.569	H	35.529	38.438	26.862	H	33.605	37.795	26.454	H	33.436	37.413	26.435
C	37.172	39.580	27.318	C	37.412	39.106	27.608	C	35.731	37.810	26.890	C	35.419	37.593	26.814
C	37.473	40.462	28.278	C	37.882	40.129	28.378	C	36.257	38.722	27.713	C	36.030	38.636	27.456
H	36.938	41.364	28.348	H	37.546	41.175	28.371	H	35.990	39.790	27.610	H	35.736	39.717	27.440
N	38.249	39.868	29.257	N	38.784	39.667	29.267	N	37.256	38.187	28.569	N	37.013	38.021	28.186
H	38.728	40.400	30.029	H	39.383	40.252	29.847	H	37.817	38.704	29.271	H	37.650	38.459	28.863
C	38.599	38.610	28.956	C	39.039	38.307	29.014	C	37.380	36.862	28.352	C	37.056	36.664	28.110
C	39.473	37.693	29.551	C	39.876	37.309	29.609	C	38.107	35.854	28.982	C	37.885	35.748	28.775
H	39.997	37.950	30.440	H	40.437	37.644	30.493	H	38.801	36.133	29.736	H	38.709	36.047	29.385
C	39.628	36.453	28.906	C	40.134	36.087	28.929	C	38.040	34.605	28.551	C	37.737	34.409	28.520
H	40.189	35.659	29.331	H	40.926	35.424	29.166	H	38.650	33.855	29.014	H	38.355	33.670	28.978
C	38.967	36.172	27.720	C	39.278	35.741	27.865	C	37.062	34.252	27.547	C	36.791	34.119	27.524
H	39.170	35.250	27.122	H	39.334	34.823	27.385	H	36.847	33.174	27.422	H	36.530	33.080	27.244
C	38.145	37.160	27.139	C	38.272	36.591	27.485	C	36.342	35.238	26.929	C	36.003	35.006	26.806
H	37.569	37.034	26.260	H	37.553	36.321	26.754	H	35.584	34.882	26.164	H	35.314	34.606	26.072
C	37.898	38.396	27.742	C	38.162	37.918	27.986	C	36.440	36.607	27.253	C	36.091	36.371	27.155
C	35.283	38.815	24.030	C	35.559	38.372	24.237	C	33.252	37.067	24.143	C	32.964	36.708	23.982
O	35.157	37.596	23.910	O	35.503	37.124	24.007	O	33.062	35.834	23.962	O	32.695	35.524	23.918
N	34.303	39.620	23.497	N	34.726	39.255	23.619	N	32.402	38.005	23.642	N	31.982	37.592	23.513
C	33.855	40.832	24.080	C	34.327	40.486	24.242	C	32.382	39.426	24.168	C	31.998	39.025	23.768
H	34.471	41.624	23.632	H	34.988	41.296	23.952	H	33.348	39.857	24.159	H	32.618	39.452	23.068
H	33.898	40.830	25.168	H	34.262	40.228	25.292	H	31.967	39.297	25.140	H	32.218	39.263	24.856
C	32.405	41.022	23.486	C	32.927	40.682	23.703	C	31.320	40.148	23.300	C	30.628	39.543	23.529

H	32.231	42.033	23.268	H	32.789	41.747	23.487	H	31.496	41.114	22.928	H	30.568	40.578	23.093
H	31.560	40.702	24.182	H	32.155	40.305	24.425	H	30.450	40.424	23.928	H	30.093	39.531	24.437
C	32.462	40.255	22.174	C	32.829	39.926	22.386	C	30.782	39.218	22.177	C	30.046	38.535	22.632
H	32.935	40.761	21.391	H	33.260	40.507	21.617	H	31.196	39.445	21.162	H	30.323	38.826	21.617
H	31.510	39.861	21.871	H	31.860	39.577	22.112	H	29.675	39.252	22.176	H	29.006	38.439	22.750
C	33.247	39.056	22.559	C	33.739	38.751	22.665	C	31.276	37.824	22.646	C	30.688	37.207	22.984
H	32.627	38.285	23.056	H	33.063	38.057	23.227	H	30.450	37.503	23.265	H	30.240	36.707	23.787
C	33.797	38.305	21.357	C	34.201	38.037	21.421	C	31.369	36.774	21.557	C	30.800	36.144	21.849
O	33.182	37.381	20.865	O	33.597	37.078	20.991	O	30.474	35.966	21.469	O	30.027	35.147	21.891
N	35.007	34.589	23.029	N	35.163	34.226	22.782	N	31.725	32.913	22.972	N	31.633	32.431	23.193
H	34.777	35.550	23.193	H	34.874	35.192	22.839	H	31.857	33.935	22.878	H	31.406	33.304	22.836
C	34.069	33.626	23.516	C	34.215	33.240	23.280	C	30.603	32.330	23.743	C	30.605	31.659	23.914
H	34.420	33.324	24.507	H	34.624	32.933	24.218	H	31.001	32.147	24.822	H	31.033	31.409	24.926
C	32.642	34.179	23.471	C	32.766	33.733	23.580	C	29.376	33.167	23.936	C	29.312	32.449	24.298
H	32.419	34.243	22.406	H	32.221	33.805	22.597	H	28.923	33.265	22.952	H	28.773	32.614	23.433
C	31.584	33.216	23.957	C	31.960	32.630	24.322	C	28.514	32.421	24.999	C	28.399	31.551	25.174
H	30.596	33.547	23.588	H	30.906	32.871	24.395	H	27.661	33.020	25.396	H	27.445	31.984	25.404
H	31.749	32.233	23.663	H	31.929	31.672	23.757	H	28.110	31.483	24.643	H	28.112	30.713	24.572
H	31.538	33.223	25.021	H	32.354	32.515	25.370	H	29.137	32.113	25.796	H	28.880	31.216	26.144
C	32.403	35.577	23.921	C	32.649	35.128	24.153	C	29.676	34.539	24.551	C	29.592	33.827	25.022
H	32.504	35.677	24.999	H	32.954	35.142	25.213	H	29.817	34.484	25.607	H	29.956	33.562	26.039
H	33.073	36.219	23.453	H	33.323	35.769	23.551	H	30.565	34.890	24.025	H	30.394	34.417	24.541
C	31.064	36.209	23.523	C	31.303	35.804	24.002	C	28.517	35.577	24.364	C	28.362	34.721	25.140
H	30.260	35.517	23.861	H	31.261	36.657	24.694	H	28.425	35.651	23.267	H	28.676	35.471	25.854
H	30.765	37.090	24.126	H	31.178	36.238	22.955	H	27.698	35.299	24.978	H	28.097	35.226	24.207
H	31.033	36.387	22.466	H	30.569	35.009	24.208	H	28.943	36.485	24.741	H	27.472	34.209	25.411
C	34.077	32.335	22.603	C	34.190	31.919	22.418	C	30.181	31.016	23.070	C	30.337	30.329	23.181
O	34.028	32.398	21.356	O	33.973	32.140	21.200	O	29.879	30.991	21.921	O	29.919	30.480	22.030

N	23.246	32.627	31.185	N	23.849	32.841	30.593	N	21.946	34.273	33.925	N	22.137	33.959	33.942
H	23.025	32.688	32.190	H	23.686	33.082	31.573	H	22.360	34.663	34.779	H	22.469	34.482	34.763
C	24.500	31.919	30.827	C	25.114	32.174	30.162	C	22.608	33.111	33.276	C	22.876	32.734	33.543
H	24.598	31.882	29.736	H	25.251	32.340	29.057	H	22.196	33.011	32.269	H	22.625	32.482	32.491
C	25.715	32.814	31.207	C	26.312	32.904	30.847	C	24.123	33.388	33.294	C	24.351	33.070	33.644
H	25.580	33.717	30.638	H	26.247	33.954	30.783	H	24.381	33.914	34.189	H	24.499	34.154	33.737
H	25.682	32.942	32.255	H	26.251	32.672	31.942	H	24.664	32.428	33.437	H	24.740	32.675	34.527
C	27.098	32.107	30.962	C	27.727	32.485	30.341	C	24.824	34.170	32.120	C	25.027	32.523	32.373
H	27.293	31.277	31.564	H	27.824	31.449	30.582	H	25.820	34.533	32.441	H	26.006	32.900	32.358
C	27.376	31.820	29.439	C	27.681	32.576	28.830	C	24.837	33.356	30.768	C	25.217	30.973	32.198
H	26.614	31.076	29.172	H	27.199	31.685	28.441	H	23.824	33.017	30.551	H	25.984	30.839	31.464
H	27.202	32.758	28.895	H	27.161	33.455	28.531	H	25.125	34.002	29.943	H	25.461	30.527	33.212
H	28.327	31.514	29.163	H	28.660	32.742	28.448	H	25.437	32.497	30.889	H	24.200	30.557	31.922
C	28.216	33.085	31.464	C	28.879	33.294	30.930	C	24.127	35.536	31.809	C	24.503	33.075	31.054
H	29.137	32.652	31.463	H	29.773	32.884	30.431	H	23.100	35.366	31.560	H	23.862	32.371	30.557
H	28.190	33.953	30.866	H	28.824	34.394	30.810	H	24.168	36.242	32.636	H	24.132	34.050	31.127
H	28.054	33.389	32.548	H	29.098	33.066	31.935	H	24.531	35.984	30.932	H	25.488	33.012	30.543
C	24.451	30.424	31.226	C	25.117	30.679	30.361	C	22.378	31.774	34.090	C	22.430	31.495	34.330
O	24.691	29.500	30.399	O	25.752	29.925	29.656	O	22.039	30.764	33.492	O	22.112	30.469	33.729
N	21.885	38.521	35.619	N	21.617	37.905	35.418	N	22.644	40.472	37.744	N	22.490	40.675	38.106
H	21.991	39.388	36.219	H	21.543	38.403	36.257	H	22.931	41.273	38.419	H	22.736	41.535	38.589
C	23.105	37.754	35.380	C	22.872	37.211	35.138	C	23.812	39.667	37.261	C	23.538	39.693	37.652
H	22.941	36.705	35.194	H	22.635	36.089	34.991	H	23.532	38.661	37.530	H	23.163	38.657	37.861
C	23.720	38.337	34.080	C	23.484	37.738	33.838	C	23.982	39.799	35.716	C	23.801	39.830	36.144
H	23.044	38.168	33.279	H	22.929	37.269	32.960	H	23.113	39.524	35.133	H	22.922	39.381	35.580
H	23.862	39.361	34.313	H	23.429	38.852	33.833	H	24.177	40.854	35.616	H	23.914	40.902	35.818
C	25.056	37.800	33.680	C	24.878	37.324	33.595	C	25.218	39.022	35.259	C	25.053	39.197	35.661
N	25.362	36.483	33.240	N	25.327	35.982	33.328	N	25.401	37.660	35.173	N	25.231	37.849	35.290

H	24.726	35.733	33.298	H	24.707	35.197	33.485	H	24.633	37.061	35.352	H	24.500	37.067	35.475
C	26.708	36.377	32.935	C	26.715	36.031	33.244	C	26.506	37.343	34.445	C	26.377	37.717	34.602
H	27.284	35.524	32.766	H	27.316	35.085	33.352	H	26.974	36.400	34.113	H	26.843	36.832	34.150
N	27.226	37.577	33.225	N	27.122	37.301	33.321	N	27.128	38.550	34.136	N	27.041	38.934	34.673
C	26.261	38.449	33.677	C	26.001	38.140	33.431	C	26.403	39.585	34.703	C	26.221	39.835	35.324
H	26.417	39.456	33.991	H	26.062	39.181	33.578	H	26.721	40.634	34.588	H	26.459	40.905	35.383
C	24.058	38.045	36.628	C	23.900	37.396	36.301	C	25.145	39.810	38.112	C	24.876	39.774	38.484
O	24.461	37.081	37.307	O	24.337	36.337	36.788	O	25.686	38.830	38.565	O	25.437	38.874	39.073
CL	31.555	35.051	27.575	CL	32.408	38.117	27.402	CL	26.818	36.443	27.803	CL	27.142	37.296	29.895
C	30.205	35.368	28.736	C	31.587	36.860	29.098	C	27.984	36.715	29.075	C	28.762	37.973	30.184
H	29.910	34.400	29.095	H	32.481	36.247	29.169	H	28.224	37.782	29.171	H	28.855	38.615	29.308
H	30.483	36.092	29.565	H	31.580	37.868	29.665	H	27.544	36.422	30.066	H	28.728	38.577	31.120
C	28.995	35.919	27.940	C	30.372	36.327	28.449	C	29.221	35.937	28.877	C	29.765	36.929	30.046
C	29.476	37.025	27.044	C	29.644	37.260	27.595	C	28.812	34.520	28.396	C	29.457	35.637	29.439
H	29.931	36.681	26.142	H	29.550	38.286	28.021	H	29.723	33.818	28.378	H	28.553	35.189	29.887
H	30.288	37.574	27.573	H	28.673	36.901	27.412	H	28.406	34.441	27.365	H	30.379	35.050	29.740
H	28.759	37.860	26.864	H	30.153	37.433	26.586	H	28.020	34.074	29.016	H	29.242	35.758	28.361
CL	28.423	34.487	26.925	CL	30.905	34.835	27.525	CL	30.251	36.648	27.543	CL	30.674	37.920	27.988
H	28.104	36.115	28.554	H	29.676	35.986	29.282	H	29.923	35.802	29.781	H	30.760	37.274	30.141

Unless otherwise stated, PMF profiles shown in Figures S1-S8 were displayed using a 5-point Savitzky-Golay smoothing procedure for visualization purposes only. All activation barriers and stationary-point energies reported in the manuscript were determined from the original unsmoothed PMF data. The (S)-1,2-DBP-C2 pathway, reported previously,[1] is included here as a reference to enable direct comparison across all studied reaction variants. The corresponding graphs have been adapted from Sowińska et al., Archives of Biochemistry and Biophysics 2023, 743, and are reproduced under the terms of the CC BY 4.0 license.

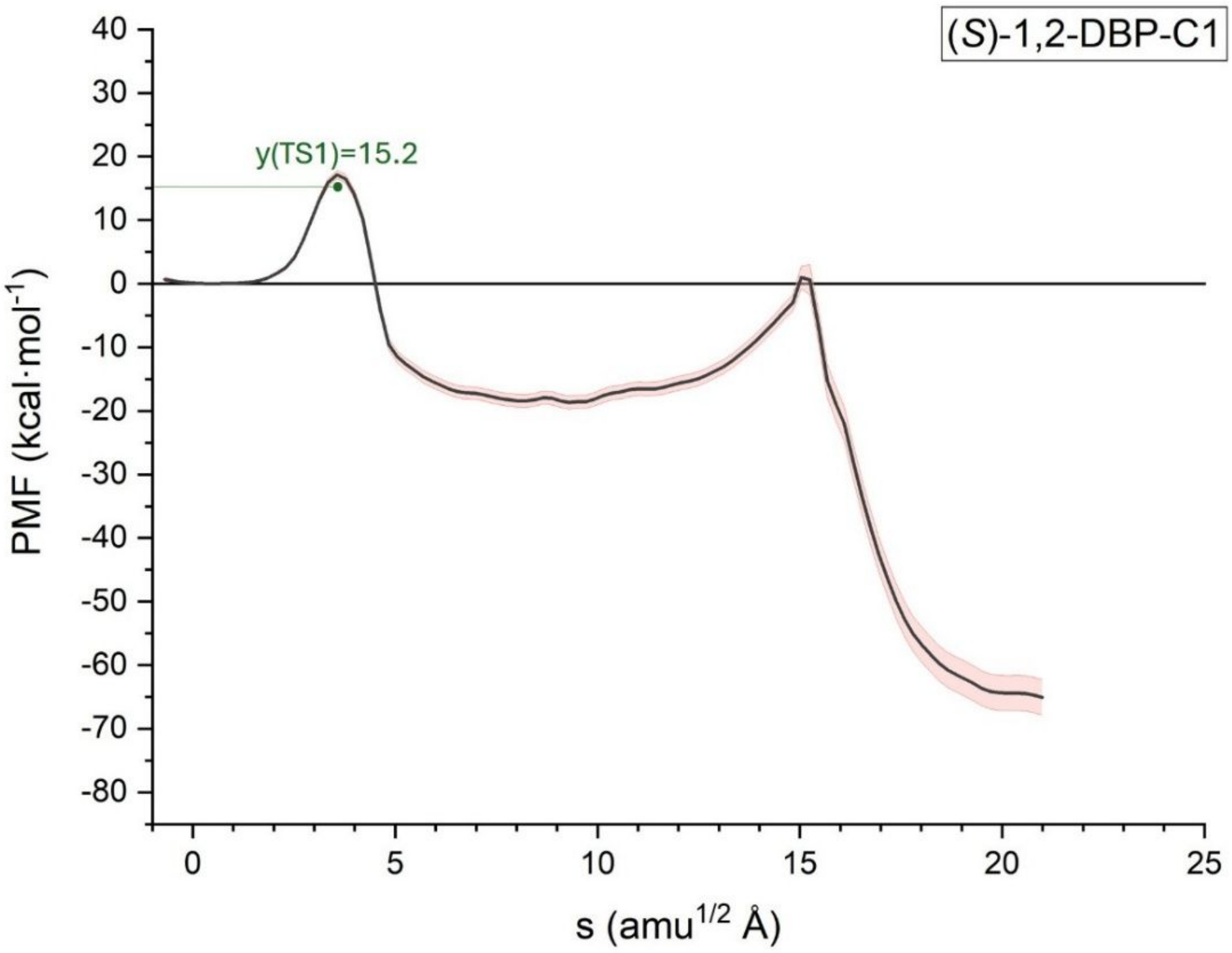

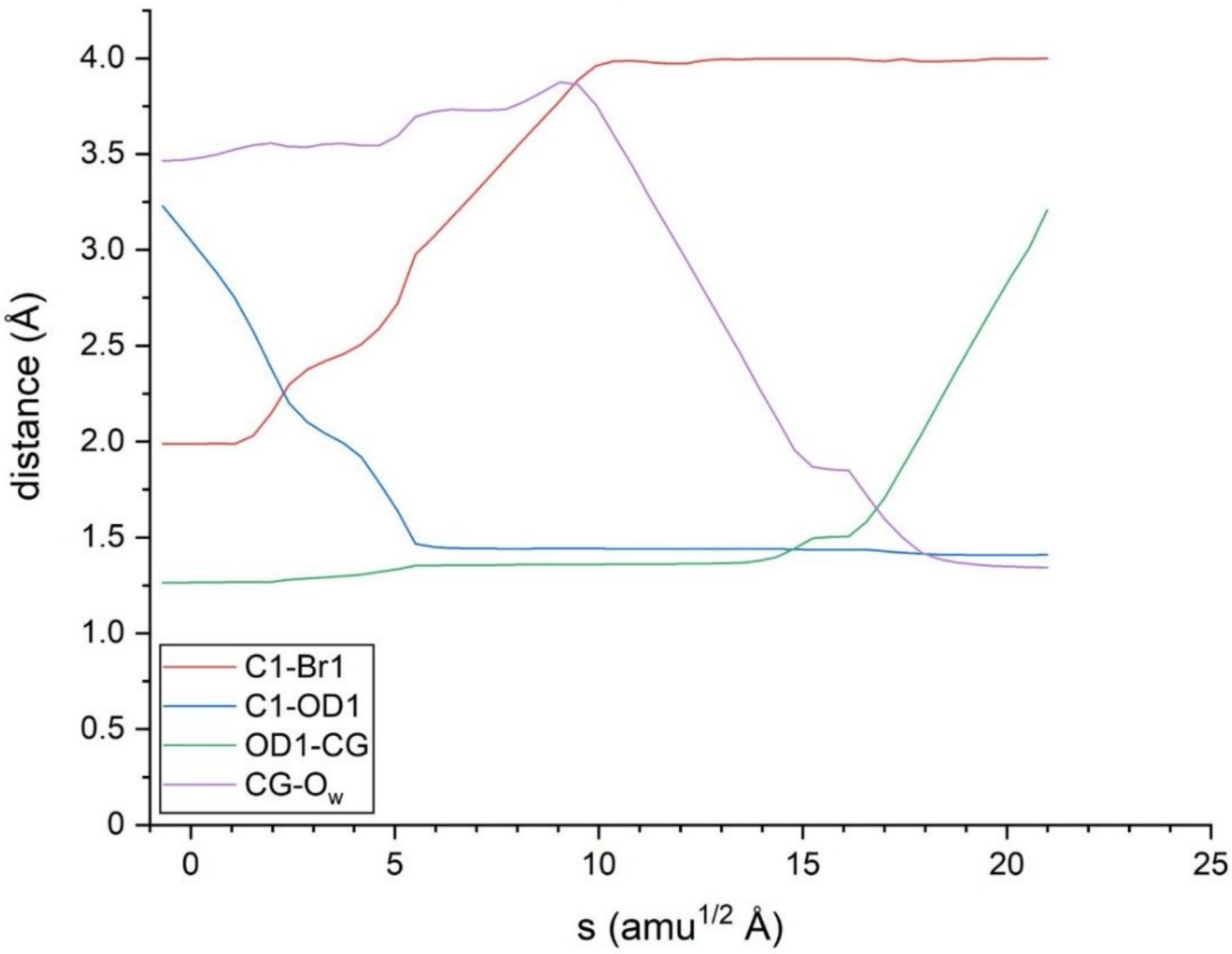

Figure S1. Free energy profile [A] and evolution of the main CVs along the MFEP [B] obtained for the LinB reaction with (*S*)-1,2-DBP in the C1 pathway. The red area shows the 95%

confidence interval in the free energy. The activation barrier reported in Table 1 (15.2 kcal·mol⁻¹) was determined from the original unsmoothed PMF data and is indicated by the green line.

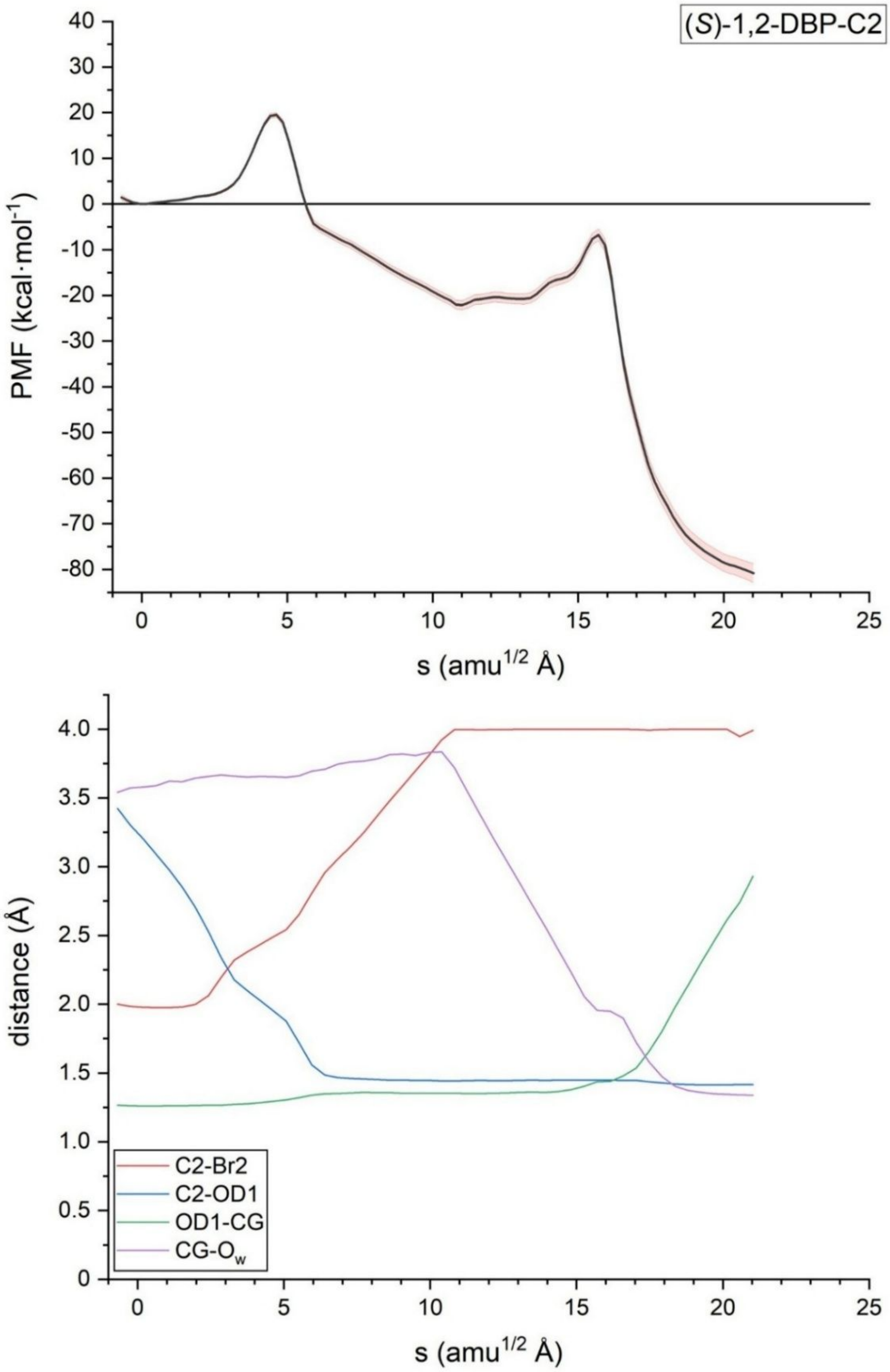

Figure S2. Free energy profile [A] and evolution of the main CVs along the MFEP [B] obtained for the LinB reaction with (*S*)-1,2-DBP in the C2 pathway. The red area shows the 95% confidence interval in the free energy. This pathway, reported previously,[1] is included here as a reference to enable direct comparison across all studied reaction variants.

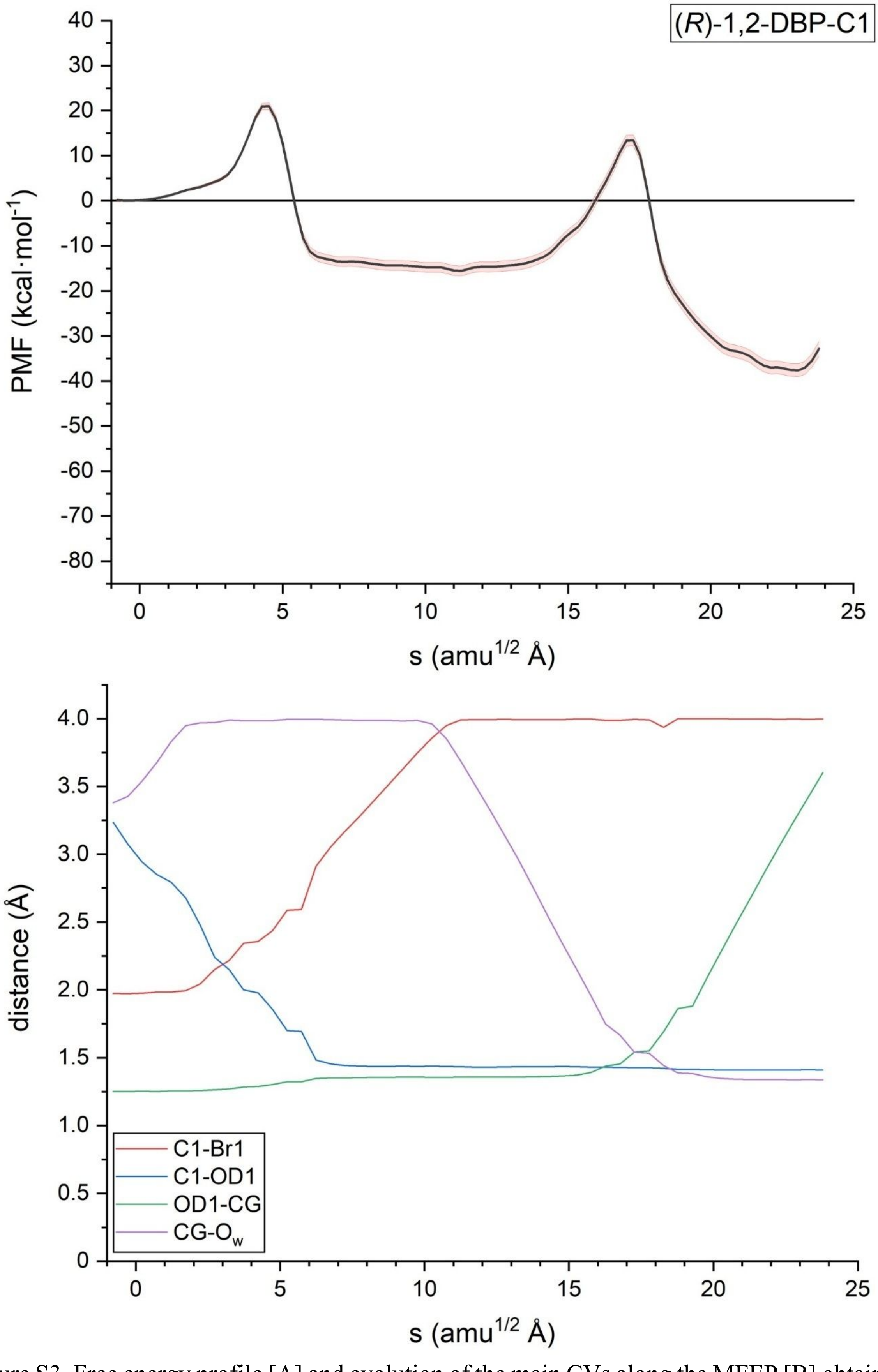

Figure S3. Free energy profile [A] and evolution of the main CVs along the MFEP [B] obtained for the LinB reaction with (*R*)-1,2-DBP in the C1 pathway. The red area shows the 95% confidence interval in the free energy.

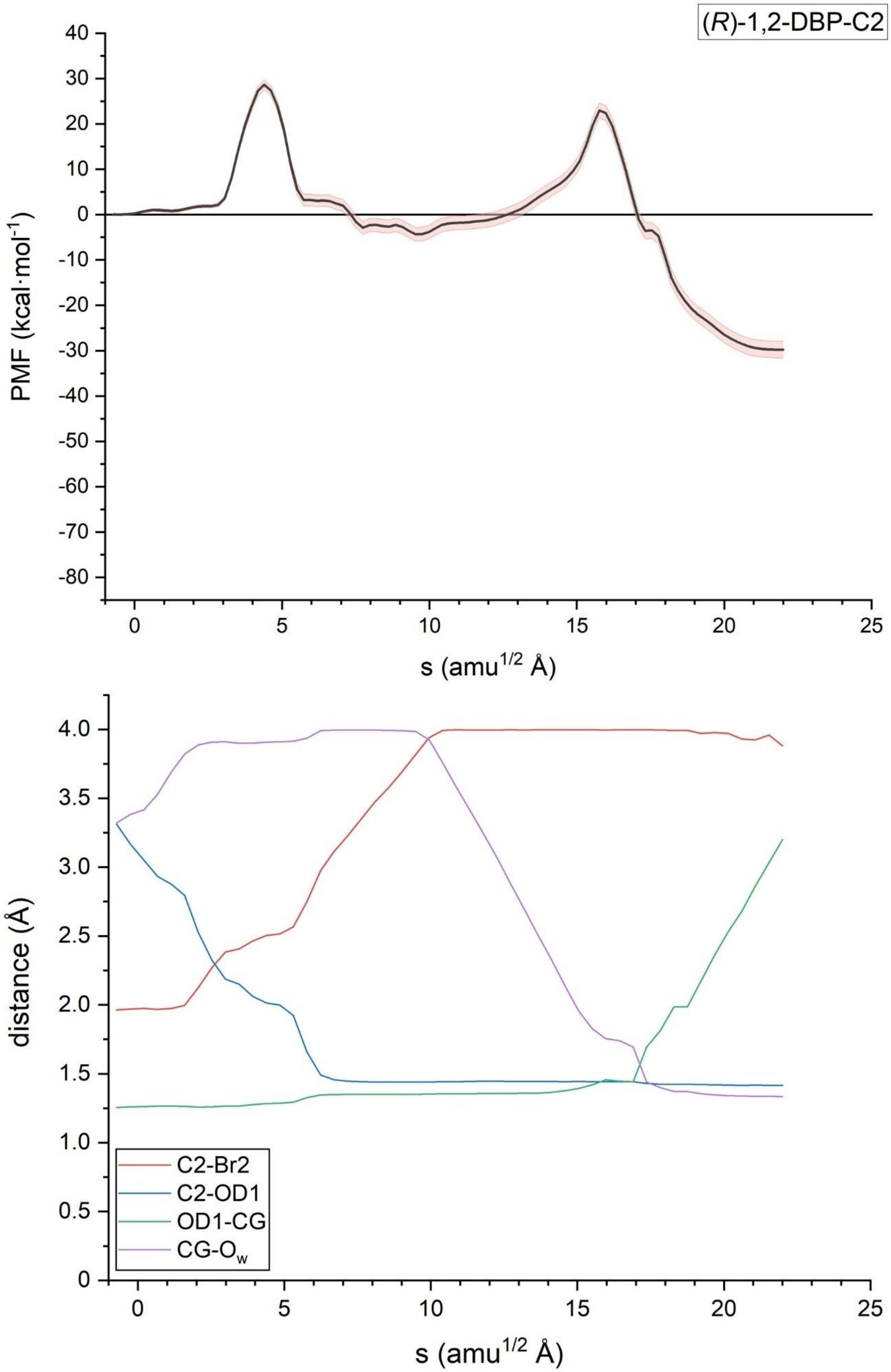

Figure S4. Free energy profile [A] and evolution of the main CVs along the MFEP [B] obtained for the LinB reaction with (*R*)-1,2-DBP in the C2 pathway. The red area shows the 95% confidence interval in the free energy.

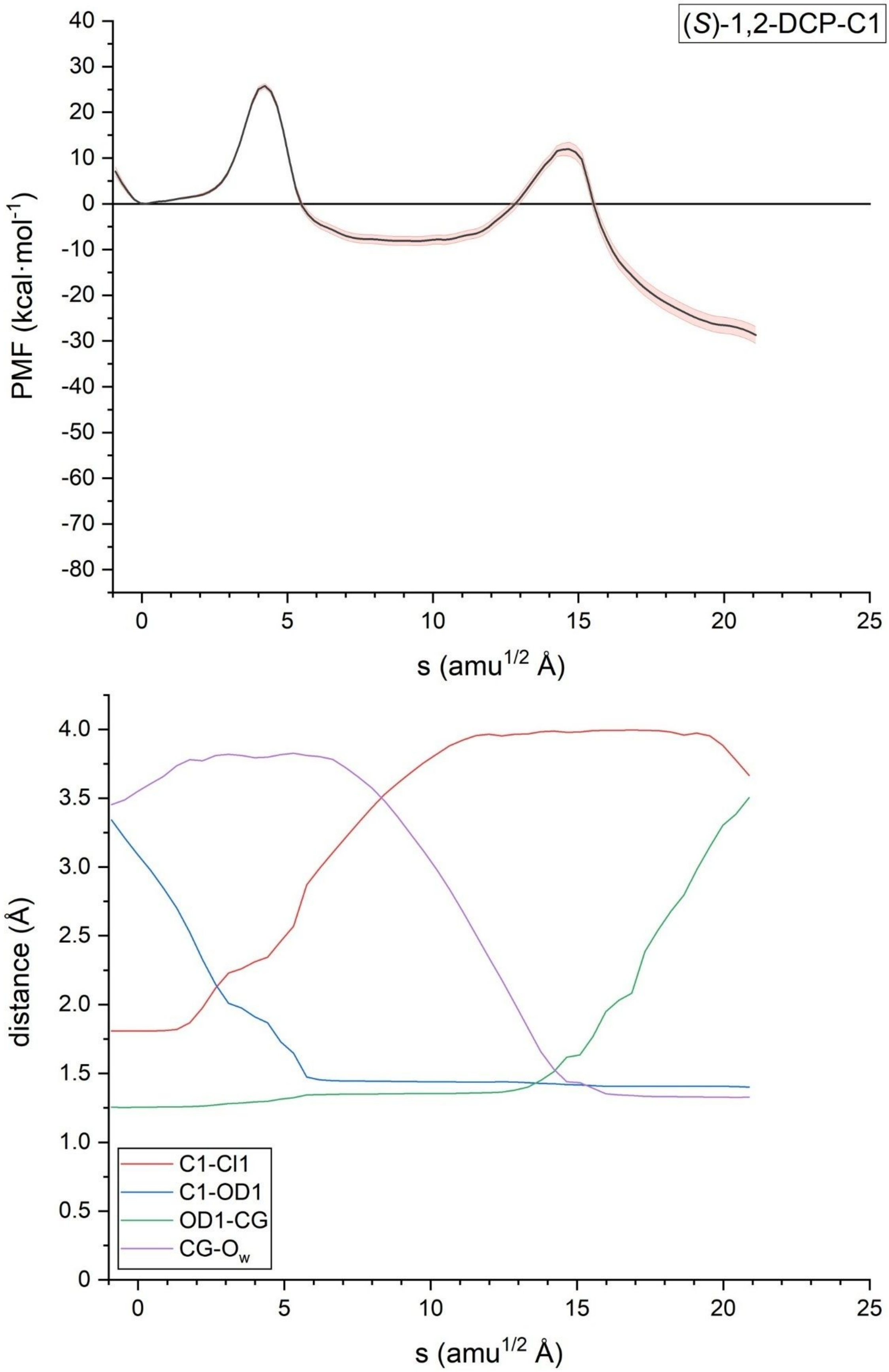

Figure S5. Free energy profile [A] and evolution of the main CVs along the MFEP [B] obtained for the LinB reaction with (*S*)-1,2-DCP in the C1 pathway. The red area shows the 95% confidence interval in the free energy.

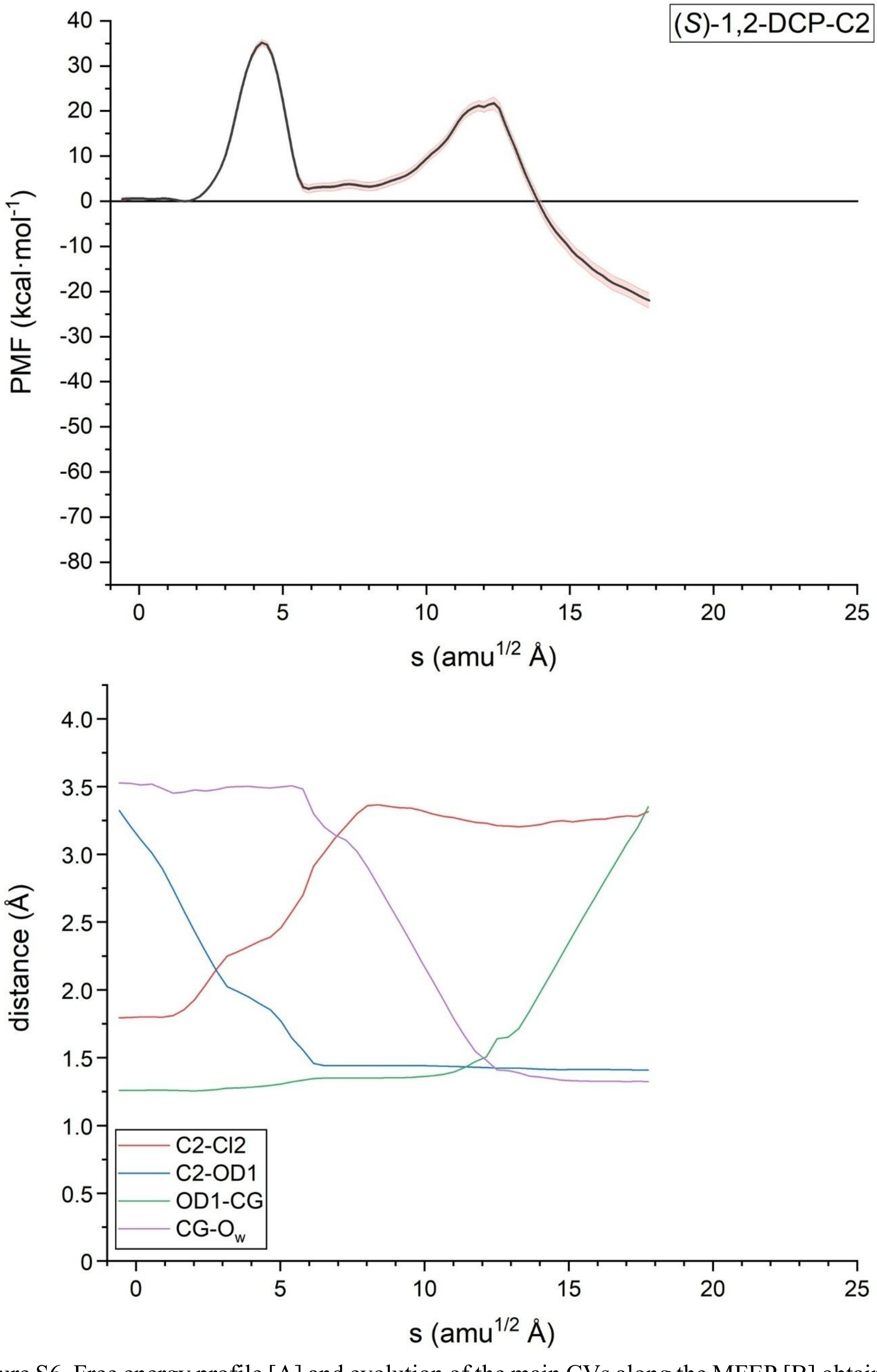

Figure S6. Free energy profile [A] and evolution of the main CVs along the MFEP [B] obtained for the LinB reaction with (*S*)-1,2-DCP in the C2 pathway. The red area shows the 95% confidence interval in the free energy.

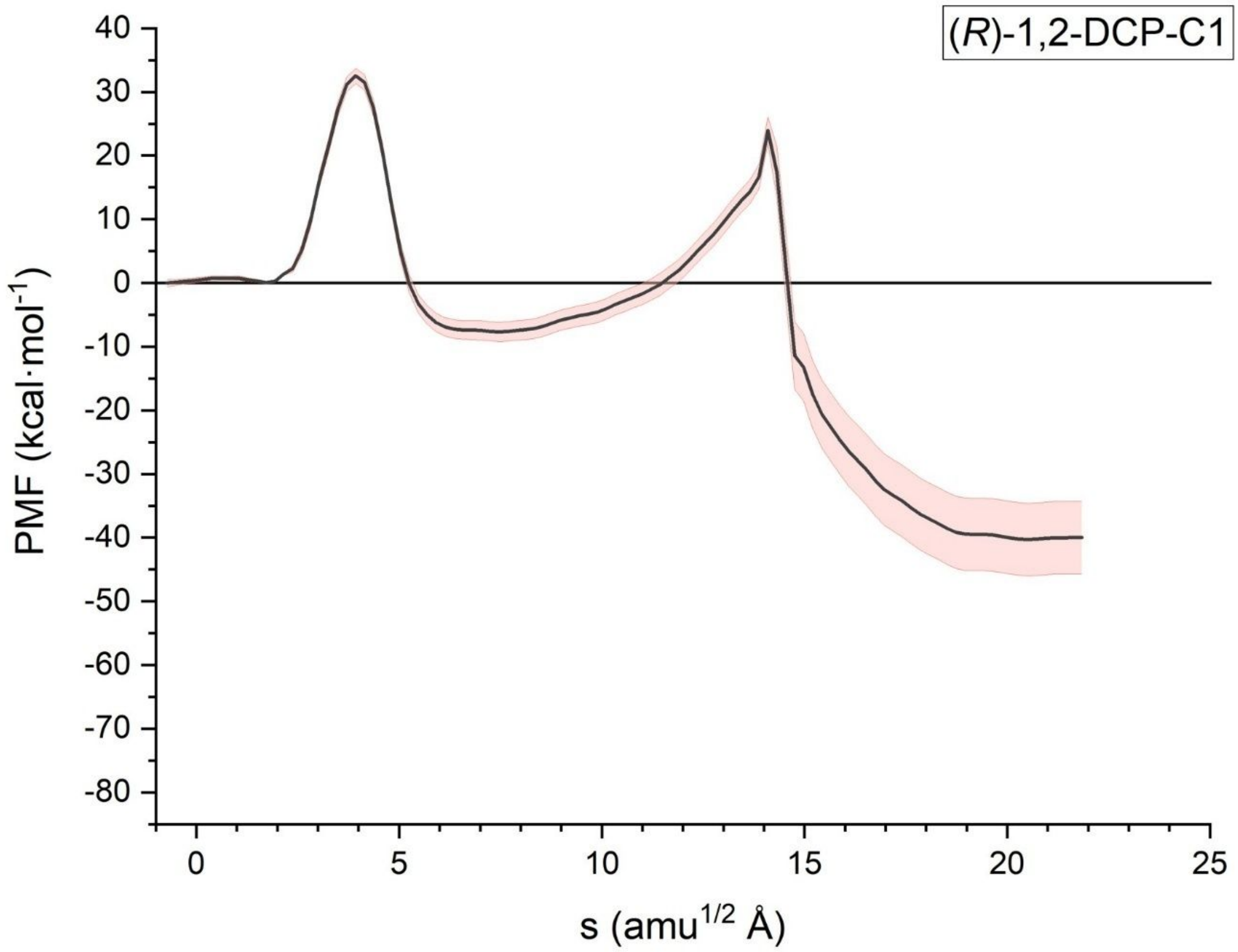

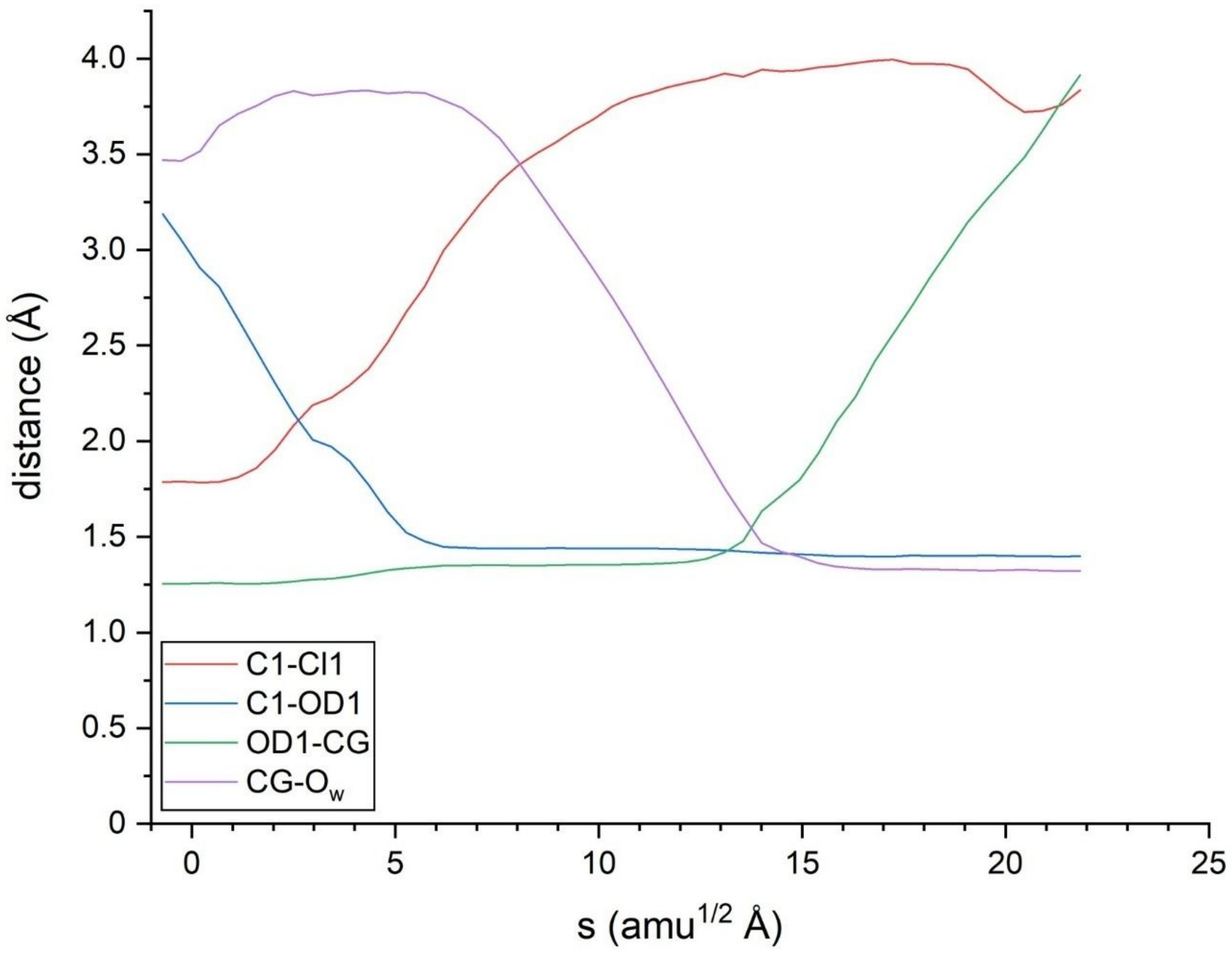

Figure S7. Free energy profile [A] and evolution of the main CVs along the MFEP [B] obtained for the LinB reaction with (*R*)-1,2-DCP in the C1 pathway. The red area shows the 95% confidence interval in the free energy.

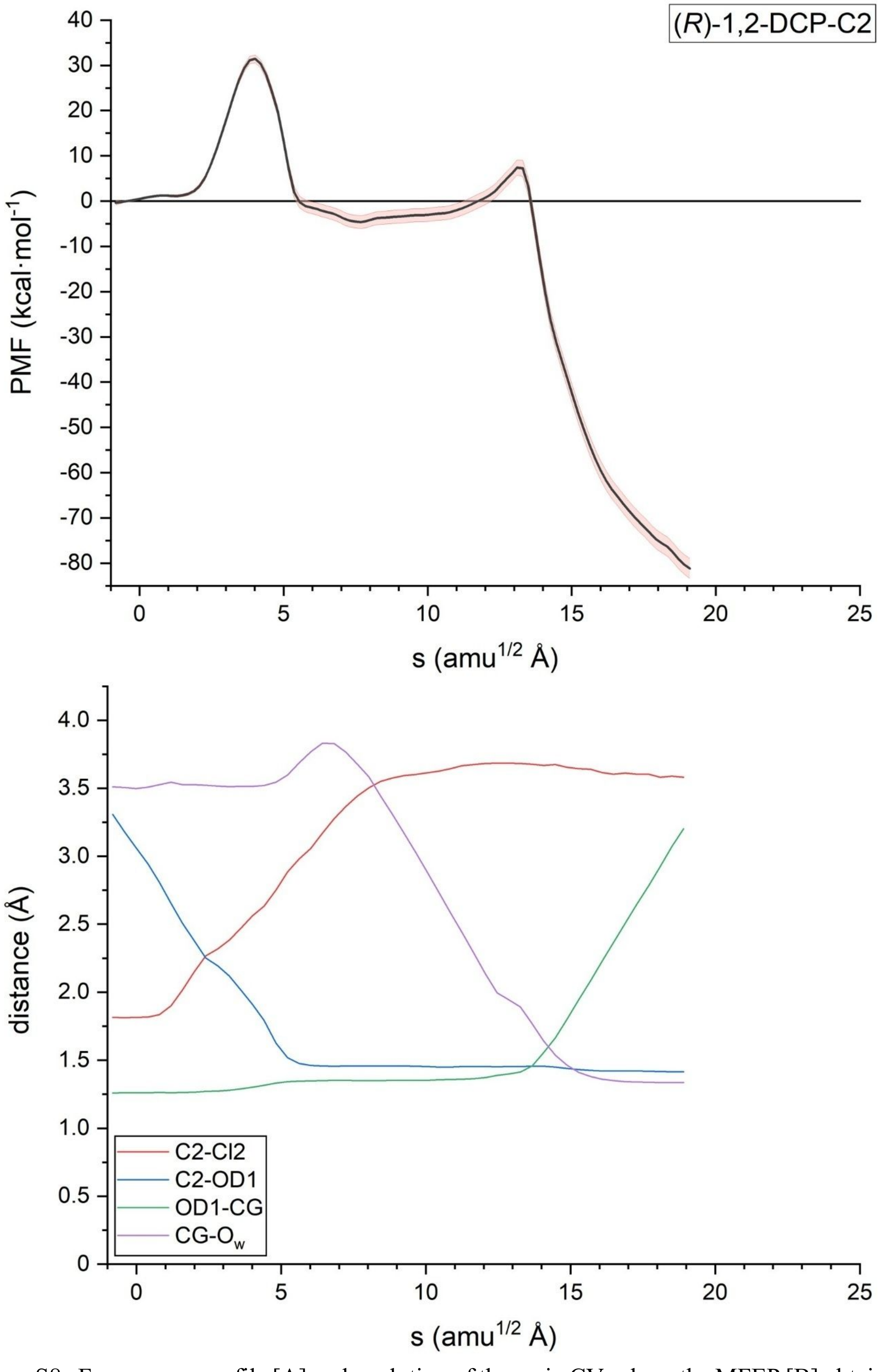

Figure S8. Free energy profile [A] and evolution of the main CVs along the MFEP [B] obtained for the LinB reaction with (*R*)-1,2-DCP in the C2 pathway. The red area shows the 95% confidence interval in the free energy.

Table S10. SAPT2+ interaction energy decomposition for the selected amino acid-DBP complexes. The reported values represent means calculated from results obtained for all representative structures selected through cluster analysis, with the corresponding standard errors of the mean (SEM). The lower section of the table provides the summed SAPT components for the selected key residues (N38, D108, W109, E132, I211). Values are given in kcal mol⁻¹. Basis set aug-cc-pVDZ.

		(*S*)-C1 [RC]		(*S*)-C1 [TS1]		(*S*)-C2 [RC]		(*S*)-C2 [TS1]		(*R*)-C1 [RC]		(*R*)-C1 [TS1]		(*R*)-C2 [RC]		(*R*)-C2 [TS1]	
		Mean	SEM	Mean	SEM	Mean	SEM	Mean	SEM	Mean	SEM	Mean	SEM	Mean	SEM	Mean	SEM
N38	Electrostatics	-2.5	1.1	-8.3	1.1	-0.7	0.5	-3.2	0.7	-0.2	0.6	-2.7	2.3	0.1	0.4	-5.1	1.3
	Exchange	4.2	2.1	15.7	3.5	1.6	1.0	5.6	1.6	3.2	2.6	4.9	3.4	0.1	0.1	8.3	1.8
	Induction	-0.8	0.4	-4.8	1.0	-0.5	0.3	-1.2	0.3	-0.6	0.4	-1.4	0.9	-0.1	0.0	-2.4	0.6
	Dispersion	-2.7	0.9	-6.7	1.1	-1.9	0.8	-4.7	0.7	-2.2	1.3	-2.7	1.3	-0.7	0.1	-5.1	0.5
	Total-SAPT2+	-1.8	0.5	-4.1	0.4	-1.5	0.5	-3.5	0.7	0.3	0.2	-1.9	1.2	-0.6	0.4	-4.3	0.7
D108	Electrostatics	-15.2	2.4	-44.2	1.6	-15.5	1.9	-65.5	1.7	-9.3	0.3	-47.4	4.1	-19.0	0.5	-59.0	2.6
	Exchange	12.2	4.8	59.1	2.4	14.9	4.7	94.8	2.1	3.2	0.5	69.6	6.0	21.2	1.5	82.5	4.8
	Induction	-8.0	1.4	-35.6	1.9	-8.8	1.5	-42.4	0.5	-4.5	0.4	-28.2	3.6	-12.2	0.6	-37.4	1.7
	Dispersion	-5.4	1.1	-14.4	0.4	-6.0	0.8	-20.1	0.3	-2.9	0.4	-14.2	1.2	-6.6	0.2	-18.6	0.6
	Total-SAPT2+	-16.4	0.9	-35.2	1.8	-15.3	0.6	-33.2	0.8	-13.5	0.6	-20.2	2.9	-16.6	0.1	-32.5	1.1
W109	Electrostatics	-4.4	1.0	-7.8	1.6	-3.8	0.6	-3.7	0.5	-2.5	1.2	-4.1	2.3	-2.5	1.5	-6.0	1.3
	Exchange	6.7	2.2	14.5	3.6	6.7	1.0	8.2	0.5	9.1	2.2	8.6	3.1	5.9	1.3	10.4	2.5
	Induction	-1.8	0.5	-3.5	0.8	-1.4	0.3	-1.5	0.1	-2.2	0.5	-2.2	0.8	-1.3	0.3	-2.6	0.6
	Dispersion	-4.6	0.9	-7.7	0.9	-5.2	0.4	-6.2	0.3	-6.3	0.5	-6.8	1.0	-5.1	0.3	-7.0	0.8
	Total-SAPT2+	-4.0	0.3	-4.5	0.6	-3.6	0.5	-3.2	0.4	-1.8	0.5	-4.5	1.1	-3.1	0.8	-5.1	0.3
E132	Electrostatics	-2.2	0.1	-2.4	0.2	-2.0	0.1	-1.6	0.0	-0.3	0.1	-0.9	0.4	-0.4	0.6	-1.5	0.2
	Exchange	0.0	0.0	0.0	0.0	0.0	0.0	0.0	0.0	0.0	0.0	0.0	0.0	0.0	0.0	0.0	0.0
	Induction	-0.3	0.0	-0.4	0.0	-0.3	0.0	-0.3	0.0	-0.1	0.0	-0.2	0.0	-0.2	0.0	-0.2	0.0
	Dispersion	-0.1	0.0	-0.1	0.0	-0.1	0.0	-0.1	0.0	0.0	0.0	-0.1	0.0	-0.1	0.0	-0.1	0.0
	Total-SAPT2+	-2.6	0.1	-2.8	0.2	-2.5	0.1	-2.0	0.1	-0.4	0.1	-1.2	0.3	-0.7	0.6	-1.7	0.2
I134	Electrostatics	-0.1	0.1	0.0	0.1	-0.6	0.2	-0.5	0.3	-1.3	0.4	-0.8	0.0	-0.6	0.1	-0.1	0.0
	Exchange	0.6	0.3	0.4	0.2	2.1	0.6	1.9	0.9	3.1	1.1	2.4	0.0	1.8	0.3	0.9	0.1
	Induction	-0.1	0.0	-0.1	0.0	-0.2	0.1	-0.2	0.1	-0.4	0.1	-0.4	0.1	-0.3	0.1	-0.2	0.0

	Dispersion	-1.3	0.4	-0.9	0.3	-2.2	0.4	-1.7	0.3	-2.6	0.4	-2.7	0.1	-2.4	0.3	-1.3	0.1
	Total-SAPT2+	-0.9	0.3	-0.6	0.2	-0.9	0.1	-0.5	0.2	-1.1	0.3	-1.4	0.2	-1.4	0.1	-0.7	0.0
F143	Electrostatics	-0.4	0.3	-0.1	0.1	-0.1	0.2	-0.2	0.1	-0.2	0.1	-0.2	0.0	-0.2	0.1	0.0	0.1
	Exchange	0.7	0.3	0.3	0.2	0.6	0.4	0.0	0.0	0.2	0.1	0.1	0.0	0.4	0.2	0.0	0.0
	Induction	-0.1	0.1	-0.1	0.0	-0.1	0.1	-0.1	0.0	0.0	0.0	0.0	0.0	-0.1	0.0	0.0	0.0
	Dispersion	-1.1	0.3	-0.7	0.2	-0.9	0.4	-0.4	0.1	-0.9	0.3	-0.7	0.1	-1.3	0.3	-0.3	0.1
	Total-SAPT2+	-0.9	0.3	-0.6	0.2	-0.6	0.3	-0.6	0.2	-0.8	0.3	-0.8	0.1	-1.1	0.2	-0.3	0.1
F151	Electrostatics	-1.0	0.4	-1.2	0.3	-0.7	0.3	-1.4	0.2	-0.3	0.2	-0.3	0.1	-0.9	0.3	-1.1	0.4
	Exchange	3.0	0.8	2.4	0.6	2.3	0.7	3.4	0.6	1.8	0.7	1.2	0.6	2.4	0.7	2.6	0.7
	Induction	-0.4	0.1	-0.5	0.1	-0.3	0.1	-0.6	0.1	-0.2	0.1	-0.2	0.0	-0.3	0.1	-0.5	0.1
	Dispersion	-3.1	0.3	-2.9	0.4	-2.7	0.3	-3.0	0.3	-2.3	0.4	-1.8	0.5	-2.5	0.3	-2.7	0.4
	Total-SAPT2+	-1.5	0.2	-2.1	0.3	-1.5	0.2	-1.6	0.1	-1.1	0.2	-1.1	0.1	-1.2	0.1	-1.6	0.2
F169	Electrostatics	-0.9	0.6	-0.7	0.4	-0.5	0.3	-0.9	0.3	-0.1	0.3	-0.2	0.1	-0.2	0.1	-1.1	0.5
	Exchange	2.0	1.5	1.7	1.0	1.1	0.6	1.6	0.8	1.1	0.6	0.3	0.1	0.4	0.2	3.2	1.1
	Induction	-0.3	0.2	-0.3	0.1	-0.2	0.1	-0.4	0.1	-0.1	0.1	-0.1	0.0	-0.1	0.0	-0.6	0.2
	Dispersion	-1.9	0.8	-1.9	0.6	-1.1	0.4	-2.1	0.5	-1.7	0.4	-0.9	0.0	-1.0	0.4	-3.2	0.5
	Total-SAPT2+	-1.0	0.2	-1.2	0.2	-0.7	0.2	-1.6	0.2	-0.8	0.1	-0.9	0.0	-0.9	0.3	-1.8	0.2
V173	Electrostatics	-0.1	0.0	-0.1	0.0	-0.1	0.0	-0.1	0.0	0.0	0.0	0.0	0.0	0.0	0.0	-0.1	0.0
	Exchange	0.0	0.0	0.0	0.0	0.0	0.0	0.0	0.0	0.0	0.0	0.0	0.0	0.0	0.0	0.0	0.0
	Induction	0.0	0.0	0.0	0.0	0.0	0.0	0.0	0.0	0.0	0.0	0.0	0.0	0.0	0.0	0.0	0.0
	Dispersion	-0.1	0.0	-0.1	0.0	0.0	0.0	-0.1	0.0	0.0	0.0	0.0	0.0	-0.1	0.0	-0.1	0.0
	Total-SAPT2+	-0.2	0.0	-0.2	0.0	-0.1	0.0	-0.2	0.0	0.0	0.0	-0.1	0.0	-0.1	0.1	-0.2	0.0
W207	Electrostatics	-0.4	0.1	-1.0	0.1	-0.1	0.1	-1.1	0.1	-0.3	0.4	-0.4	0.4	-0.1	0.1	-1.3	0.4
	Exchange	0.9	0.2	1.5	0.4	0.5	0.1	1.9	0.4	1.2	0.7	1.0	0.5	0.2	0.2	2.3	0.7
	Induction	-0.2	0.0	-0.4	0.1	-0.1	0.0	-0.3	0.1	-0.2	0.1	-0.3	0.1	-0.1	0.0	-0.5	0.1
	Dispersion	-1.7	0.1	-2.1	0.3	-1.3	0.3	-2.4	0.2	-2.0	0.6	-1.8	0.5	-1.0	0.3	-2.6	0.4
	Total-SAPT2+	-1.5	0.1	-2.1	0.1	-1.0	0.2	-1.9	0.0	-1.3	0.4	-1.4	0.5	-0.9	0.2	-2.1	0.2
P208	Electrostatics	-1.0	0.2	-0.5	0.1	-0.8	0.3	-0.5	0.1	-0.2	0.2	-0.3	0.2	0.0	0.2	-1.0	0.3
	Exchange	1.9	0.5	1.0	0.2	1.5	0.5	0.7	0.2	1.4	0.5	0.7	0.1	0.2	0.1	1.9	0.5
	Induction	-0.3	0.0	-0.2	0.0	-0.2	0.1	-0.1	0.0	-0.2	0.1	-0.1	0.0	-0.1	0.0	-0.3	0.1

	Dispersion	-3.2	0.4	-2.3	0.2	-2.2	0.5	-1.9	0.3	-2.4	0.4	-2.0	0.1	-1.2	0.3	-3.0	0.4
	Total-SAPT2+	-2.4	0.3	-2.1	0.2	-1.7	0.3	-1.9	0.2	-1.3	0.2	-1.6	0.3	-1.0	0.4	-2.5	0.3
I211	Electrostatics	-1.6	0.7	-0.9	0.3	-2.3	0.4	-0.9	0.2	-2.4	0.2	-1.3	0.2	-3.4	0.4	-0.6	0.2
	Exchange	4.5	1.8	2.6	0.8	5.6	1.1	2.8	0.7	6.1	0.5	3.0	0.2	8.7	0.5	1.5	0.4
	Induction	-0.5	0.2	-0.3	0.1	-0.6	0.1	-0.4	0.1	-0.6	0.0	-0.4	0.0	-0.9	0.1	-0.2	0.1
	Dispersion	-3.9	1.0	-3.0	0.6	-4.8	0.4	-3.1	0.4	-5.3	0.4	-3.8	0.3	-6.0	0.1	-2.3	0.4
	Total-SAPT2+	-1.5	0.2	-1.7	0.2	-2.1	0.2	-1.5	0.0	-2.3	0.2	-2.5	0.4	-1.6	0.1	-1.6	0.2
L248	Electrostatics	-0.1	0.2	0.0	0.1	-1.2	0.5	-0.6	0.3	-1.0	0.2	-1.3	0.0	-1.1	0.3	-0.2	0.1
	Exchange	1.1	0.7	0.8	0.3	4.3	1.3	1.8	0.8	3.4	0.4	4.5	0.0	3.4	0.5	1.2	0.3
	Induction	-0.1	0.0	-0.1	0.0	-0.5	0.1	-0.3	0.1	-0.4	0.0	-0.6	0.0	-0.4	0.0	-0.3	0.1
	Dispersion	-1.7	0.5	-1.4	0.3	-3.7	0.8	-2.4	0.6	-3.1	0.1	-4.4	0.0	-3.7	0.3	-2.1	0.3
	Total-SAPT2+	-0.9	0.2	-0.7	0.2	-1.0	0.1	-1.5	0.3	-1.0	0.3	-1.7	0.1	-1.8	0.2	-1.4	0.1
H272	Electrostatics	-0.4	0.1	-0.7	0.1	-0.2	0.1	-0.7	0.1	-0.6	0.0	-0.8	0.2	-0.3	0.4	-0.8	0.3
	Exchange	0.0	0.0	0.1	0.0	0.0	0.0	0.0	0.0	0.0	0.0	0.1	0.0	0.0	0.0	0.1	0.0
	Induction	0.0	0.0	-0.1	0.0	0.0	0.0	-0.1	0.0	0.0	0.0	-0.1	0.1	-0.1	0.0	-0.2	0.0
	Dispersion	-0.2	0.0	-0.5	0.1	-0.3	0.0	-0.3	0.0	-0.1	0.0	-0.4	0.2	-0.4	0.0	-0.5	0.1
	Total-SAPT2+	-0.6	0.1	-1.2	0.2	-0.5	0.1	-1.1	0.2	-0.7	0.0	-1.2	0.3	-0.7	0.4	-1.4	0.2
N38. D108. W109. E132. P208. I211	Electrostatics	-26.9	5.6	-64.2	5.0	-25.0	3.8	-75.4	3.2	-14.9	2.7	-56.7	9.6	-25.3	3.7	-73.2	5.9
	Exchange	29.6	11.4	92.8	10.5	30.4	8.3	112.2	5.1	23.0	6.4	86.8	12.8	36.2	3.4	104.6	10.0
	Induction	-11.6	2.5	-44.8	3.9	-11.8	2.2	-45.9	1.1	-8.2	1.4	-32.6	5.4	-14.7	1.1	-43.1	3.0
	Dispersion	-19.9	4.2	-34.2	3.2	-20.3	2.9	-36.1	2.0	-19.1	3.1	-29.4	4.1	-19.7	1.1	-36.1	2.7
	Total-SAPT2+	-28.8	2.3	-50.4	3.3	-26.7	2.3	-45.3	2.1	-19.1	1.7	-31.9	6.2	-23.5	2.4	-47.8	2.8

Table S11. SAPT2+ interaction energy decomposition for the selected amino acid-DCP complexes. The reported values represent means calculated from results obtained for all representative structures selected through cluster analysis, with the corresponding standard errors of the mean (SEM). The lower section of the table provides the summed SAPT components for the selected key residues (N38, D108, W109, E132, I211). Values are given in kcal mol⁻¹. Basis set aug-cc-pVDZ.

		(*S*)-C1 [RC]		(*S*)-C1 [TS1]		(*S*)-C2 [RC]		(*S*)-C2 [TS1]		(*R*)-C1 [RC]		(*R*)-C1 [TS1]		(*R*)-C2 [RC]		(*R*)-C2 [TS1]	
		Mean	SEM	Mean	SEM	Mean	SEM	Mean	SEM	Mean	SEM	Mean	SEM	Mean	SEM	Mean	SEM
N38	Electrostatics	0.3	0.0	-8.4	0.0	0.3	0.2	-2.4	0.2	-0.4	0.1	-3.3	1.0	-0.6	0.1	-10,5	1,2
	Exchange	1.0	0.6	12.5	0.0	2.7	0.7	4.0	0.9	0.0	0.0	3.0	1.4	0.0	0.0	15,8	2,5
	Induction	-0.2	0.1	-4.2	0.0	-0.5	0.1	-1.0	0.1	0.0	0.0	-1.2	0.5	-0.1	0.0	-5,4	0,7
	Dispersion	-1.7	0.6	-5.5	0.0	-3.3	0.4	-4.1	0.3	-0.3	0.0	-2.3	0.7	-0.4	0.1	-6,8	0,6
	Total-SAPT2+	-0.7	0.1	-5.6	0.0	-0.7	0.1	-3.4	0.7	-0.7	0.1	-3.8	0.8	-1.1	0.1	-6,9	0,3
D108	Electrostatics	-14.4	0.5	-68.5	0.0	-26.0	0.6	-88.7	1.3	-18.3	1.1	-57.7	2.5	-16.9	0.7	-58,3	2,5
	Exchange	7.8	1.9	97.5	0.0	29.4	0.2	138.6	3.3	22.9	1.5	87.4	4.6	18.3	1.6	72,9	5,1
	Induction	-6.4	0.5	-49.1	0.0	-14.6	0.4	-67.4	3.9	-10.9	0.6	-40.3	1.4	-10.2	0.5	-40,8	1,5
	Dispersion	-4.9	0.7	-19.1	0.0	-8.8	0.1	-24.2	0.8	-6.3	0.2	-17.1	0.6	-6.0	0.3	-17,7	0,7
	Total-SAPT2+	-17.9	0.2	-39.1	0.0	-20.0	0.9	-41.7	2.7	-12.7	0.5	-27.8	2.5	-14.8	0.1	-43,9	1,3
W109	Electrostatics	-1.7	0.1	-4.5	0.0	-3.2	1.1	-1.8	1.0	-3.0	0.8	-6.7	1.3	-4.3	1.0	-8,4	1,3
	Exchange	2.9	0.0	6.1	0.0	7.1	1.4	3.9	1.9	5.6	1.4	11.0	2.3	7.7	2.7	12,8	2,9
	Induction	-0.7	0.0	-2.0	0.0	-1.4	0.2	-0.8	0.4	-0.8	0.2	-2.8	0.5	-1.7	0.5	-3,4	0,7
	Dispersion	-3.8	0.0	-5.4	0.0	-6.4	0.6	-4.0	1.0	-4.3	0.5	-6.5	0.6	-4.5	0.6	-7,4	0,8
	Total-SAPT2+	-3.3	0.1	-5.8	0.0	-3.9	0.6	-2.6	0.4	-2.5	0.5	-5.0	0.3	-2.7	0.6	-6,4	0,3
E132	Electrostatics	-1.4	0.1	-2.1	0.0	-0.9	0.1	-0.5	0.0	-2.0	0.2	-2.6	0.1	-2.2	0.2	-1,9	0,2
	Exchange	0.0	0.0	0.0	0.0	0.0	0.0	0.0	0.0	0.0	0.0	0.0	0.0	0.0	0.0	0,0	0,0
	Induction	-0.2	0.0	-0.4	0.0	-0.2	0.0	-0.3	0.0	-0.3	0.0	-0.3	0.0	-0.3	0.0	-0,3	0,0
	Dispersion	-0.1	0.0	-0.1	0.0	-0.1	0.0	-0.1	0.0	-0.1	0.0	-0.1	0.0	-0.1	0.0	-0,1	0,0
	Total-SAPT2+	-1.6	0.1	-2.6	0.0	-1.2	0.1	-0.8	0.0	-2.4	0.2	-3.0	0.1	-2.6	0.2	-2,3	0,2
I134	Electrostatics	-0.2	0.1	-0.6	0.0	-0.2	0.1	-0.1	0.1	-1.2	0.3	-0.9	0.1	-1.1	0.3	-0,2	0,1
	Exchange	0.9	0.2	2.3	0.0	0.7	0.3	0.5	0.2	3.5	0.7	2.6	0.3	3.6	0.7	1,2	0,2
	Induction	-0.1	0.0	-0.3	0.0	-0.1	0.0	-0.1	0.0	-0.4	0.1	-0.2	0.0	-0.3	0.1	-0,2	0,0
	Dispersion	-1.2	0.1	-2.3	0.0	-1.1	0.2	-1.0	0.1	-3.2	0.2	-2.5	0.1	-2.8	0.3	-1,4	0,1

	Total-SAPT2+	-0.7	0.0	-0.8	0.0	-0.6	0.0	-0.8	0.0	-1.2	0.3	-1.0	0.2	-0.6	0.1	-0,6	0,1
F143	Electrostatics	-0.4	0.2	-0.1	0.0	-0.2	0.0	-0.2	0.1	-0.7	0.3	-0.3	0.3	-0.5	0.4	-0,1	0,0
	Exchange	0.8	0.3	0.1	0.0	0.2	0.1	0.0	0.0	1.9	0.6	1.0	0.5	1.8	0.3	0,2	0,1
	Induction	-0.1	0.0	0.0	0.0	-0.1	0.0	0.0	0.0	-0.3	0.1	-0.1	0.1	-0.3	0.0	-0,1	0,0
	Dispersion	-1.5	0.2	-0.6	0.0	-0.8	0.2	-0.4	0.0	-1.9	0.3	-1.5	0.4	-1.9	0.0	-0,7	0,2
	Total-SAPT2+	-1.2	0.1	-0.7	0.0	-0.8	0.2	-0.6	0.1	-0.9	0.1	-0.9	0.2	-0.9	0.1	-0,6	0,1
F151	Electrostatics	-0.9	0.1	-0.3	0.0	-1.1	0.3	-1.0	0.0	-0.5	0.1	-1.1	0.1	-0.2	0.0	-0,8	0,5
	Exchange	2.0	0.6	0.3	0.0	2.0	0.6	1.9	0.1	1.0	0.4	2.3	0.4	0.8	0.0	3,5	1,4
	Induction	-0.3	0.1	-0.1	0.0	-0.3	0.1	-0.5	0.0	-0.1	0.0	-0.4	0.1	-0.2	0.0	-0,6	0,2
	Dispersion	-2.5	0.3	-1.3	0.0	-2.3	0.4	-2.4	0.2	-1.5	0.3	-2.7	0.2	-2.0	0.0	-3,2	0,8
	Total-SAPT2+	-1.7	0.0	-1.4	0.0	-1.8	0.2	-1.9	0.1	-1.1	0.1	-1.9	0.1	-1.5	0.1	-1,1	0,3
F169	Electrostatics	0.0	0.0	-0.3	0.0	-0.1	0.1	-1.1	0.6	0.0	0.0	-0.2	0.0	0.0	0.0	-0,4	0,2
	Exchange	0.3	0.2	0.3	0.0	0.1	0.0	2.1	1.3	0.0	0.0	0.1	0.1	0.0	0.0	1,0	0,4
	Induction	-0.1	0.0	-0.1	0.0	0.0	0.0	-0.5	0.3	0.0	0.0	0.0	0.0	0.0	0.0	-0,3	0,1
	Dispersion	-1.0	0.2	-1.2	0.0	-0.7	0.0	-2.2	0.8	-0.2	0.0	-0.5	0.2	-0.2	0.0	-1,6	0,3
	Total-SAPT2+	-0.8	0.1	-1.3	0.0	-0.7	0.1	-1.7	0.3	-0.2	0.0	-0.6	0.2	-0.3	0.1	-1,3	0,2
V173	Electrostatics	-0.1	0.0	-0.1	0.0	-0.1	0.0	-0.1	0.0	-0.1	0.0	-0.1	0.0	-0.1	0.0	-0,1	0,0
	Exchange	0.0	0.0	0.0	0.0	0.0	0.0	0.0	0.0	0.0	0.0	0.0	0.0	0.0	0.0	0,0	0,0
	Induction	0.0	0.0	0.0	0.0	0.0	0.0	0.0	0.0	0.0	0.0	0.0	0.0	0.0	0.0	0,0	0,0
	Dispersion	-0.1	0.0	-0.1	0.0	0.0	0.0	-0.1	0.0	0.0	0.0	0.0	0.0	0.0	0.0	0,0	0,0
	Total-SAPT2+	-0.1	0.0	-0.2	0.0	-0.1	0.0	-0.2	0.0	-0.1	0.0	-0.1	0.0	-0.1	0.0	-0,1	0,0
W207	Electrostatics	-0.2	0.0	-0.8	0.0	-1.0	0.2	-0.6	0.0	0.2	0.0	-0.2	0.1	0.1	0.0	-1,1	0,2
	Exchange	0.1	0.0	0.3	0.0	2.4	0.8	0.8	0.0	0.0	0.0	0.4	0.1	0.0	0.0	1,4	0,4
	Induction	-0.1	0.0	-0.2	0.0	-0.3	0.1	-0.1	0.0	0.0	0.0	-0.2	0.0	0.0	0.0	-0,5	0,1
	Dispersion	-0.8	0.1	-1.1	0.0	-2.7	0.4	-1.7	0.0	-0.3	0.0	-1.2	0.2	-0.5	0.1	-2,0	0,2
	Total-SAPT2+	-1.0	0.1	-1.8	0.0	-1.7	0.1	-1.6	0.0	-0.1	0.1	-1.1	0.1	-0.5	0.1	-2,2	0,2
P208	Electrostatics	-0.7	0.1	-0.4	0.0	-0.8	0.2	-0.5	0.0	0.1	0.0	-0.3	0.1	0.0	0.0	-0,9	0,1
	Exchange	1.3	0.5	0.2	0.0	1.3	0.2	0.6	0.1	0.0	0.0	0.2	0.1	0.0	0.0	0,7	0,2
	Induction	-0.2	0.1	-0.1	0.0	-0.2	0.0	-0.1	0.0	0.0	0.0	-0.1	0.0	0.0	0.0	-0,3	0,1
	Dispersion	-2.2	0.4	-1.1	0.0	-2.4	0.1	-1.6	0.1	-0.3	0.0	-1.1	0.2	-0.4	0.1	-1,6	0,3

	Total-SAPT2+	-1.8	0.0	-1.4	0.0	-2.1	0.1	-1.6	0.0	-0.2	0.0	-1.2	0.1	-0.4	0.1	-2,1	0,2
I211	Electrostatics	-2.0	0.6	-1.2	0.0	-1.2	0.2	-1.2	0.7	-1.7	0.2	-1.6	0.3	-1.7	0.2	-0,5	0,2
	Exchange	5.8	1.9	3.4	0.0	3.2	0.5	3.7	1.8	4.1	0.5	4.1	0.8	4.3	0.6	0,9	0,3
	Induction	-0.5	0.2	-0.5	0.0	-0.4	0.1	-0.4	0.1	-0.5	0.0	-0.5	0.0	-0.5	0.0	-0,3	0,1
	Dispersion	-5.1	1.1	-3.6	0.0	-3.6	0.3	-3.4	0.5	-4.2	0.3	-4.3	0.4	-4.3	0.6	-1,7	0,2
	Total-SAPT2+	-1.7	0.0	-1.9	0.0	-2.0	0.0	-1.3	0.5	-2.4	0.0	-2.3	0.2	-2.2	0.1	-1,5	0,2
L248	Electrostatics	-0.3	0.1	-1.7	0.0	-0.5	0.2	-0.6	0.1	-1.1	0.2	-0.8	0.3	-0.4	0.2	0,0	0,1
	Exchange	1.1	0.0	4.3	0.0	1.3	0.5	1.6	0.1	3.9	0.6	2.8	0.8	2.1	0.8	0,5	0,2
	Induction	-0.2	0.0	-0.6	0.0	-0.2	0.1	-0.3	0.0	-0.5	0.1	-0.4	0.1	-0.3	0.1	-0,1	0,0
	Dispersion	-2.0	0.0	-3.9	0.0	-2.1	0.4	-2.4	0.0	-3.8	0.4	-3.0	0.4	-2.9	0.7	-1,1	0,2
	Total-SAPT2+	-1.4	0.1	-1.9	0.0	-1.4	0.2	-1.7	0.3	-1.5	0.1	-1.4	0.1	-1.5	0.2	-0,6	0,1
H272	Electrostatics	-0.5	0.1	-0.2	0.0	-0.5	0.0	-0.5	0.0	-0.4	0.2	-0.9	0.0	-0.6	0.0	-0,9	0,2
	Exchange	0.0	0.0	0.0	0.0	0.0	0.0	0.0	0.0	0.0	0.0	0.0	0.0	0.1	0.0	0,1	0,0
	Induction	0.0	0.0	-0.1	0.0	0.0	0.0	-0.1	0.0	0.0	0.0	-0.1	0.0	-0.1	0.0	-0,1	0,0
	Dispersion	-0.2	0.0	-0.4	0.0	-0.2	0.0	-0.4	0.0	-0.3	0.0	-0.3	0.0	-0.5	0.1	-0,5	0,1
	Total-SAPT2+	-0.7	0.1	-0.7	0.0	-0.7	0.1	-0.9	0.1	-0.7	0.1	-1.3	0.1	-1.1	0.2	-1,4	0,2

N38. D108. W109. E132. P208. I211	Electrostatics	-19.7	1.5	-85.0	0.0	-31.8	2.3	-95.0	3.2	-25.4	2.3	-72.3	5.2	-25.7	2.2	-80,5	5,4
	Exchange	18.7	5.0	119.7	0.0	43.6	3.0	150.9	8.0	32.5	3.5	105.7	9.2	30.4	4.9	103,1	11,0
	Induction	-8.2	0.9	-56.4	0.0	-17.3	0.8	-70.0	4.4	-12.6	0.9	-45.3	2.5	-12.7	1.1	-50,5	3,0
	Dispersion	-17.8	2.9	-34.7	0.0	-24.4	1.5	-37.3	2.6	-15.5	1.1	-31.4	2.4	-15.7	1.6	-35,2	2,5
	Total-SAPT2+	-27.0	0.5	-56.4	0.0	-29.9	1.8	-51.5	4.3	-20.9	1.4	-43.2	4.1	-23.8	1.3	-63,1	2,5

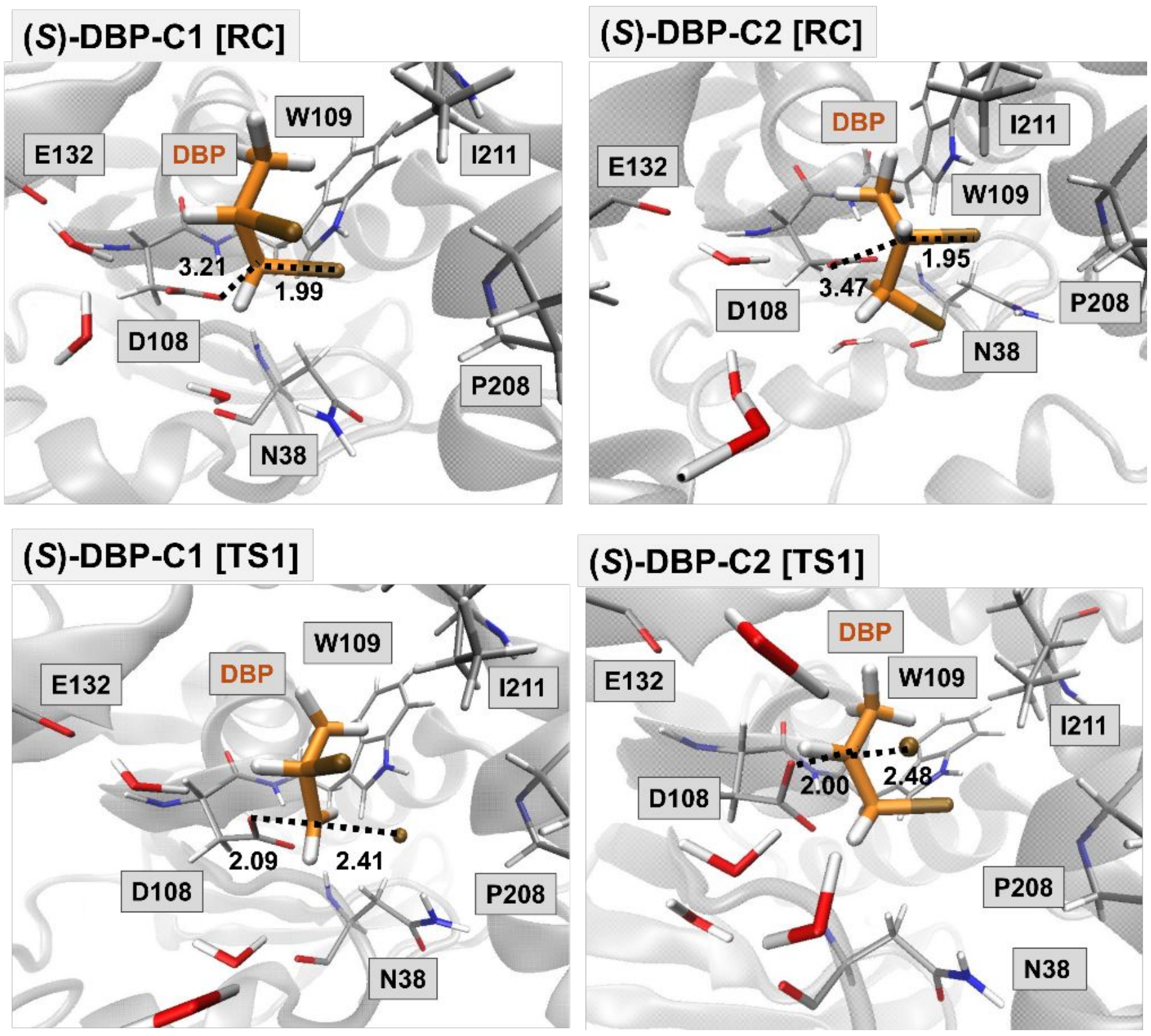

Figure S9. Representative structures of DBP in the (S)-C1 and (S)-C2 reactant complexes [RC] and their corresponding transition states [TS1]. Key distances are given in Å.

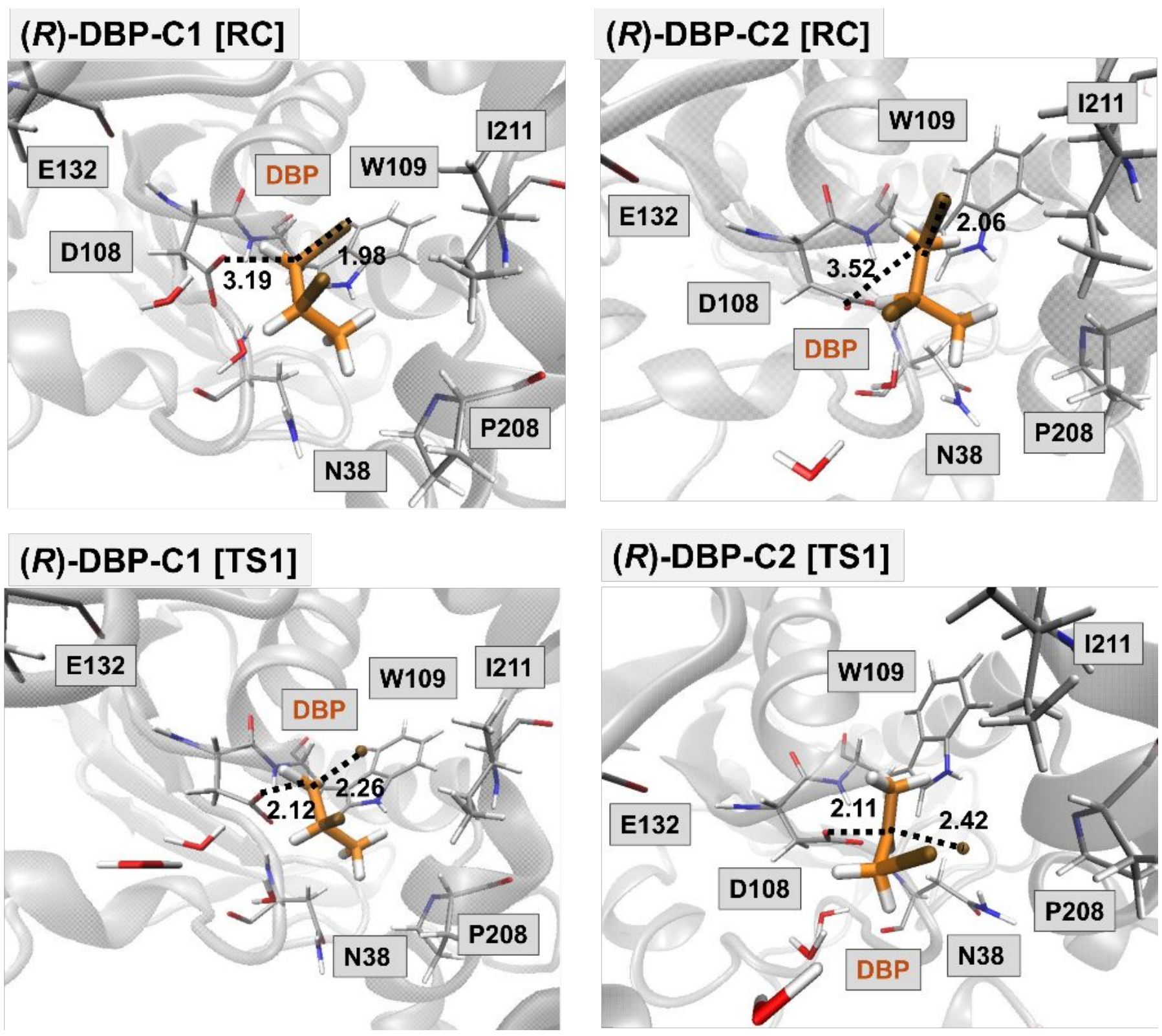

Figure S10. Representative structures of DBP in the (R)-C1 and (R)-C2 reactant complexes [RC] and their corresponding transition states [TS1]. Key distances are given in Å.

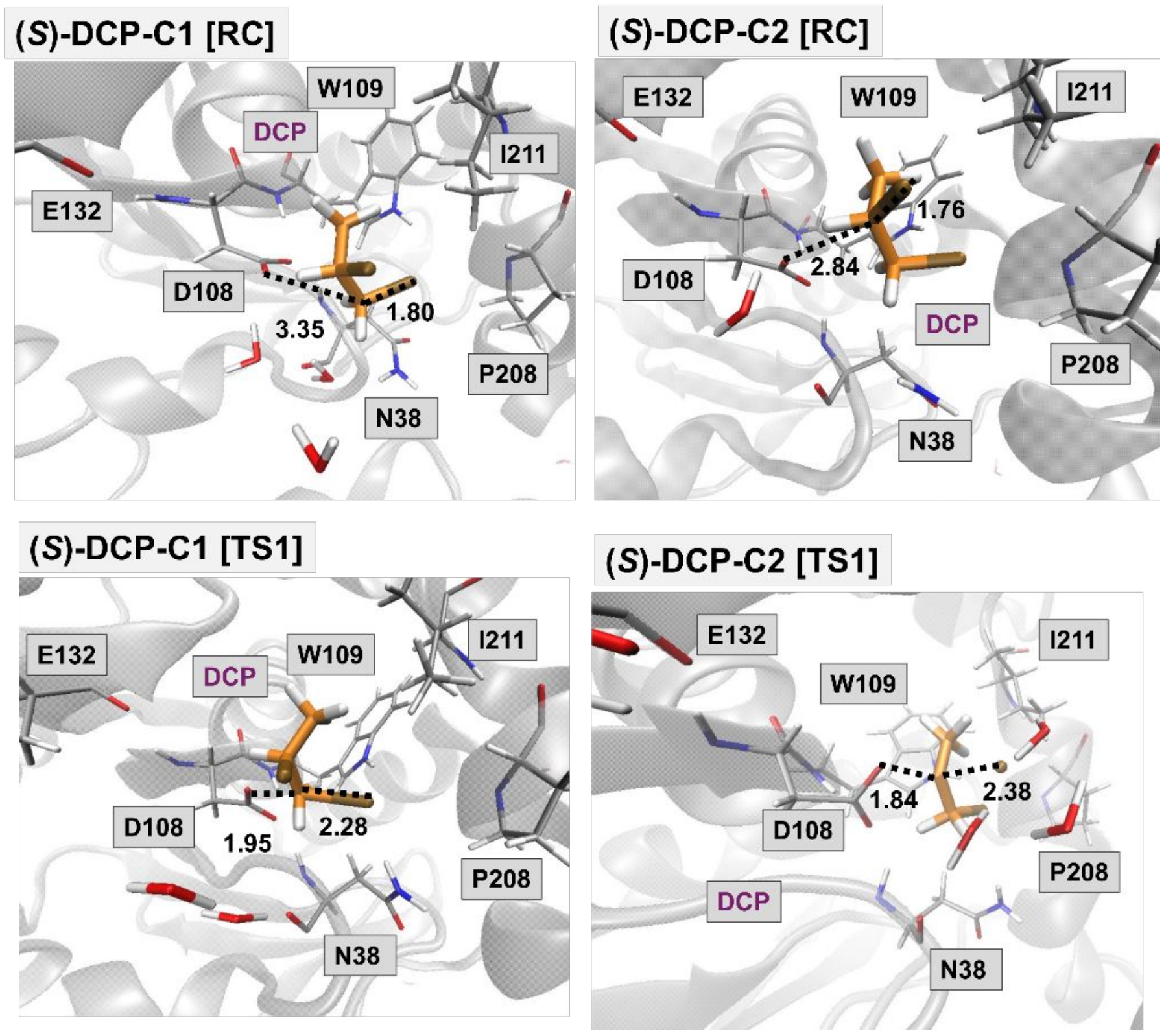

Figure S11. Representative structures of DCP in the (S)-C1 and (S)-C2 reactant complexes [RC] and their corresponding transition states [TS1]. Key distances are given in Å.

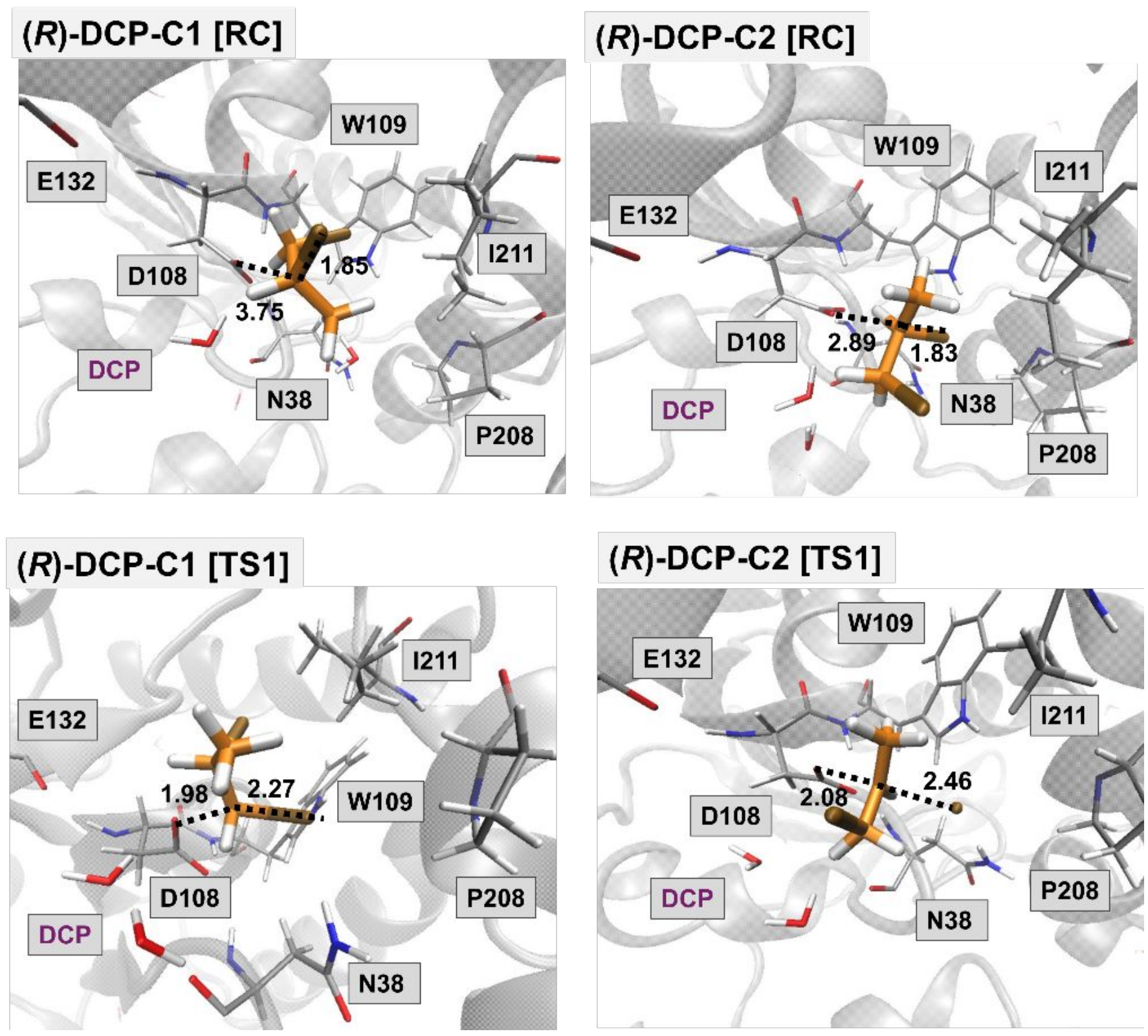

Figure S12. Representative structures of DCP in the (R)-C1 and (R)-C2 reactant complexes [RC] and their corresponding transition states [TS1]. Key distances are given in Å.

Table S12. Mean ligand–residue distances (Å) for representative structures obtained from cluster analysis, reported by ligand configuration and nucleophilic attack position, with standard errors of the mean (SEM).[8]

					L-N38	L-D108	L-P208
DBP	*S*	C1	RC	Mean	4.03	3.19	4.38
				SEM	0.41	0.01	0.50
			TS1	Mean	3.39	2.10	4.10
				SEM	0.08	0.02	0.06
		C2	RC	Mean	6.63	3.44	5.85
				SEM	0.81	0.01	0.66
			TS1	Mean	5.50	2.02	6.04
				SEM	0.28	0.01	0.19
	R	C1	RC	Mean	7.70	3.20	6.58
				SEM	0.24	0.01	0.08
			TS1	Mean	5.90	2.08	5.34
				SEM	2.48	0.04	1.32
		C2	RC	Mean	7.47	3.61	6.54
				SEM	0.47	0.07	0.35
			TS1	Mean	4.87	2.09	5.18
				SEM	0.60	0.01	0.57
DCP	*S*	C1	RC	Mean	4.64	3.32	4.05
				SEM	0.35	0.03	0.04
			TS1	Mean	3.24	1.95	4.45
				SEM	0.00	0.00	0.00
		C2	RC	Mean	5.98	2.83	6.36
				SEM	0.05	0.01	0.12
			TS1	Mean	6.30	1.85	6.89
				SEM	0.24	0.01	0.27
	R	C1	RC	Mean	6.24	2.76	5.99
				SEM	0.36	0.01	0.32
			TS1	Mean	4.31	1.97	4.65
				SEM	0.44	0.01	0.16
		C2	RC	Mean	5.34	2.89	5.14
				SEM	0.38	0.02	0.28
			TS1	Mean	3.23	2.05	4.44
				SEM	0.05	0.02	0.14

[8] *L-N38: a distance between the leaving group of a ligand and ND2 atom of N38 residue;*
L-D108: a distance between the attacked carbon atom of a ligand and nucleophile oxygen atom (OD1) of D108 residue;
L-208: a distance between the leaving group of a ligand and N atom of P208 residue.

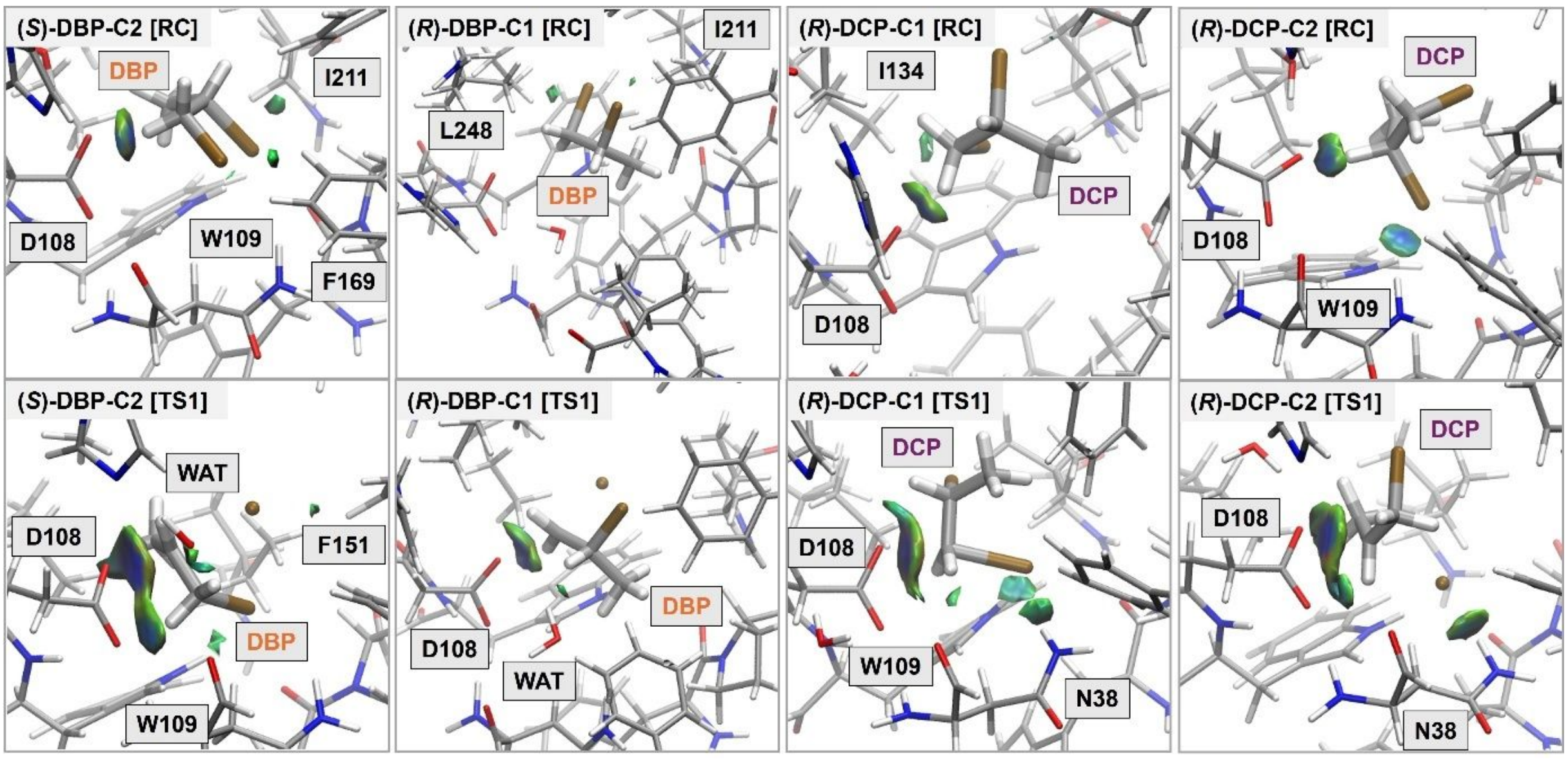

Figure S13. Weak intermolecular interaction maps for the remaining stationary structures in the LinB active site. The surfaces are color-coded according to $\mathrm{sign}(\lambda_2)\rho$ on an RGB scale to distinguish attractive (blue), repulsive (red), and weak dispersive (green) regions. Visualization was performed with an isovalue of 0.01 a.u. and a colour range of -0.05 a.u. $< \mathrm{sign}(\lambda_2)\rho <$ 0.05 a.u.

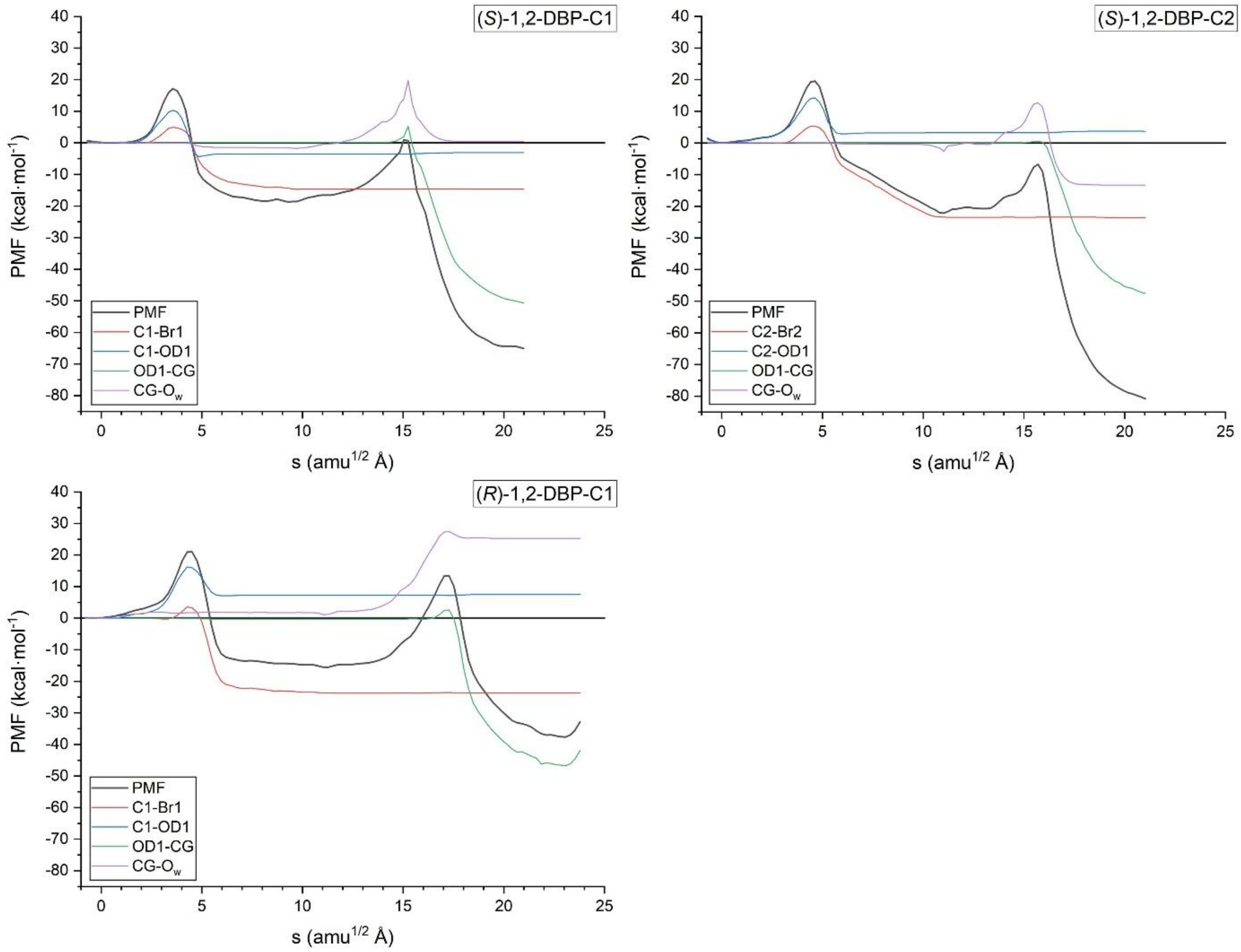

Figure S14. Free energy profiles for each CV as a function of the reaction path for LinB-DBP reactions depending on the ligand configuration (S in the top row, R in the bottom row) and the position of the nucleophilic attack (C1 in the left column, C2 in the right column). Overall PMF profile (dark grey), CVs: distance CX – BrX (red), CX – OD1 (blue), OD1 – CG (green), and CG – O_w (purple). The (S)-1,2-dbp-C2 pathway, reported previously, is included here as a reference to enable direct comparison across all discussed reaction variants, and the graph has been adapted from Sowińska et al. (*Arch. of Biochem. Bioph*. 2023, 743) licensed under CC BY 4.0.[1]

References

(1) Sowińska, A.; Rostkowski, M.; Krzemińska, A.; Englman, T.; Gelman, F.; Dybala-Defratyka, A. Insights into Generalization of the Rate-Limiting Steps of the Dehalogenation by LinB and DhaA: A Computational Approach. *Arch. Biochem. Biophys.* **2023**, *743*. https://doi.org/10.1016/j.abb.2023.109675.
